# Secondary metabolite gene clusters in the entomopathogen fungus *Metarhizium anisopliae*: genome identification and patterns of expression in a cuticle infection model

**DOI:** 10.1186/s12864-016-3067-6

**Published:** 2016-10-25

**Authors:** Nicolau Sbaraini, Rafael Lucas Muniz Guedes, Fábio Carrer Andreis, Ângela Junges, Guilherme Loss de Morais, Marilene Henning Vainstein, Ana Tereza Ribeiro de Vasconcelos, Augusto Schrank

**Affiliations:** 1 Rede Avançada em Biologia Computacional, RABICÓ, Petrópolis, RJ Brazil; 2 Centro de Biotecnologia, Programa de Pós-graduação em Biologia Celular e Molecular, Universidade Federal do Rio Grande do Sul, Porto Alegre, RS Brazil; 3 Laboratório Nacional de Computação Científica, LNCC, Petrópolis, RJ Brazil

**Keywords:** *Metarhizium* spp, Secondary metabolite biosynthetic gene clusters, Infection process, Transcriptome analysis, Biological control, Cattle tick

## Abstract

**Background:**

The described species from the *Metarhizium* genus are cosmopolitan fungi that infect arthropod hosts. Interestingly, while some species infect a wide range of hosts (host-generalists), other species infect only a few arthropods (host-specialists). This singular evolutionary trait permits unique comparisons to determine how pathogens and virulence determinants emerge. Among the several virulence determinants that have been described, secondary metabolites (SMs) are suggested to play essential roles during fungal infection. Despite progress in the study of pathogen-host relationships, the majority of genes related to SM production in *Metarhizium* spp. are uncharacterized, and little is known about their genomic organization, expression and regulation. To better understand how infection conditions may affect SM production in *Metarhizium anisopliae*, we have performed a deep survey and description of SM biosynthetic gene clusters (BGCs) in *M. anisopliae*, analyzed RNA-seq data from fungi grown on cattle-tick cuticles, evaluated the differential expression of BGCs, and assessed conservation among the *Metarhizium* genus. Furthermore, our analysis extended to the construction of a phylogeny for the following three BGCs: a tropolone/citrinin-related compound (MaPKS1), a pseurotin-related compound (MaNRPS-PKS2), and a putative helvolic acid (MaTERP1).

**Results:**

Among 73 BGCs identified in *M. anisopliae*, 20 % were up-regulated during initial tick cuticle infection and presumably possess virulence-related roles. These up-regulated BGCs include known clusters, such as destruxin, NG39x and ferricrocin, together with putative helvolic acid and, pseurotin and tropolone/citrinin-related compound clusters as well as uncharacterized clusters. Furthermore, several previously characterized and putative BGCs were silent or down-regulated in initial infection conditions, indicating minor participation over the course of infection.

Interestingly, several up-regulated BGCs were not conserved in host-specialist species from the *Metarhizium* genus, indicating differences in the metabolic strategies employed by generalist and specialist species to overcome and kill their host. These differences in metabolic potential may have been partially shaped by horizontal gene transfer (HGT) events, as our phylogenetic analysis provided evidence that the putative helvolic acid cluster in *Metarhizium* spp. originated from an HGT event.

**Conclusions:**

Several unknown BGCs are described, and aspects of their organization, regulation and origin are discussed, providing further support for the impact of SM on the *Metarhizium* genus lifestyle and infection process.

**Electronic supplementary material:**

The online version of this article (doi:10.1186/s12864-016-3067-6) contains supplementary material, which is available to authorized users.

## Background

The genus *Metarhizium* comprises entomopathogenic fungi that have been employed for the biological control of crop plagues and vector-borne diseases since these species were first described [1]. The wide range of arthropod hosts infected by *Metarhizium* spp. has resulted in a need to better understand the infection process and to improve its modulation for biocontrol. *Metarhizium* spp. are models for host-pathogen interaction studies and virulence factor discovery [2–4] as well as for the development of potential novel applications [5–7]. Additionally, this genus comprises unique evolutionary traits, harboring well-characterized transitional species with varying degrees of host specificity. Some species are host-specialists (*M. acridum* and *M. album*), some demonstrate an intermediate host range (*M. guizhouense* and *M. majus*) and some are host-generalists (*M. anisopliae*, *M. robertsii*, and *M. brunneum*) (Table 1) [8]. Comparative genomic analyses have suggested that generalists evolved from specialists via transitional species with intermediate host ranges [8].

**Table 1:** Host range of *Metarhizium* species

*Metarhizium* species	Host range	Hosts
*Metarhizium album*	Specialist	Hemiptera;
*Metarhizium acridum*	Specialist	Orthoptera;
*Metarhizium majus*	Intermediate	Coleoptera and Lepidoptera;
*Metarhizium guizhouense*	Intermediate	Coleoptera and Lepidoptera;
*Metarhizium brunneum*	Generalist	More than seven orders of insects, as well as arachnids;
*Metarhizium robertsii*	Generalist	More than seven orders of insects, as well as arachnids;
*Metarhizium anisopliae*	Generalist	More than seven orders of insects, as well as arachnids;

*Metarhizium* spp. infection begins when fungal conidia adhere to the surface of a suitable host. Host cuticle composition and fungal characteristics determine the host specificity [8, 9]. Under appropriate humidity and temperature conditions, conidia germination gives rise to the germ-tube and to a specialized infection structure, the appressorium. This structure assists the fungus in breaching the host cuticle to reach the hemocoel, where host colonization and sepsis commence, ultimately resulting in host death. During infection, several hydrolytic enzymes, such as chitinases, proteases and lipases, act as important virulence determinants [2]. These enzymes not only facilitate nutritional processes but also morphogenesis and autolytic processes in fungal development [10]. In addition to hydrolytic enzymes, secondary metabolites (SMs) are also produced by *Metarhizium* to overcome and kill the host [11].

SMs are small molecules with diverse biological activities and applications. Numerous SMs of interest have been isolated from entomopathogenic fungi in recent years (reviewed by [11]), such as beauvericin from *Beauveria bassiana*, which possesses insecticidal, antifungal, antibacterial and potent cytotoxic activities against human cells [11]. Cordycepin, an SM product from *Cordyceps militaris*, exhibits apoptotic and anti-proliferative activities against cancer cells [12], and hirsutellic acid A from *Hirsutella* spp. demonstrates activity against the malarial parasite *Plasmodium falciparum* [13]. Additionally, many subclasses of destruxins, which exert insecticide, antiviral and cytotoxic effects, have been isolated from *Metarhizium* spp. [11]. The various biotechnological applications of such compounds have aroused great interest in *Metarhizium* spp. as sources of novel control drugs [14, 15].

In fungi, genes for the biosynthesis of SMs are often arranged in clusters and are co-regulated. These biosynthetic gene clusters (BGCs) usually contain backbone genes such as polyketide synthases (PKS), non-ribosomal peptide synthetases (NRPS), hybrids (PKS-NRPS), terpene cyclases (TCs) and prenyltransferases (PTs) as well as adjacent genes that assist in regulation, transport and metabolite trimming [15, 16]. Massive sequence data availability, combined with tools to predict BGCs, have revealed that fungal genomes encode far greater numbers of SMs than previously estimated. This diversity of silent metabolites, which are not accessible under normal laboratory culture conditions, reflects habitat complexity [17] and represents great scientific and commercial opportunities [14]. Furthermore, these BGCs are also evolutionarily interesting. It has been proposed that clustering favors the survival of SM genes, and BGCs partially depend on horizontal gene transfer (HGT) for their dispersal [18]. In fact, several horizontally transferred BGCs have been described. For example, the sterigmatocystin cluster was transferred from *Aspergillus* spp. to *Podospora anserina* [19], and the homologous *ACE1* gene cluster in *Aspergillus clavatus* originated via HGT from a donor related to *Magnaporthe* spp. [20]. HGT events for BGCs have also been linked with the success of emergent pathogens, such as *Mycosphaerella populorum*, which acquired a chaetoglobosin-like cluster from an unknown donor that is potentially involved in poplar tree infection [21].

Although a vast array of SM compounds has been isolated from *Metarhizium* species [11, 22–25], few BGCs have been examined at the gene level employing functional mutants [26–30]. The deletion of five genes, specifically a gene from the serinocyclin BGC (synthesis of cyclic peptides in conidia), a gene from the NG39x BGC (synthesis of mutagenic fusarin-like compounds NG391 and NG393), a gene from metachelin BGC (synthesis of siderophore) and two PKS genes (*MrPKs1* and *MrPKs2*), did not significantly affect virulence. Until now, only the deletion of the destruxin and siderophore ferricrocin synthesis genes has been shown to affect virulence. An *M. robertsii* mutant lacking destruxin demonstrated reduced infection efficiency against *Bombyx mori* and *Locusta migratoria* [28], and an *M. robertsii* mutant lacking the siderophore ferricrocin exhibited reduced virulence in *Spodoptera exigua* [30]. Furthermore, it has been suggested that the retention of the destruxin BGC is evolutionarily related to the host range, given that host-specialists do not possesses a fully functional destruxin synthesis cluster [28]. This was also suggested in reports predicting BGCs using bioinformatics tools, which indicated that host-specialist species of *Metarhizium* have a different set of BGCs than host-generalist species [8, 31]. In general, these results suggest that the presence of a different range of SMs may be related to the narrowed virulence and specialization of host-specialist species.

Therefore, it is reasonable to assume that many BGCs, including clusters that are not expressed under normal laboratory conditions, participate in the *Metarhizium* spp. infection process. However, the activation of silent clusters, and functional gene analysis methods are laborious and time-consuming. Alternatively, to investigate genes related to infection in a genome-wide strategy, in this work we have deepened the existing knowledge of SMs in the genus *Metarhizium*. We have performed an exhaustive survey and description of BGCs in *M. anisopliae* and assessed the conservation of BGCs and related genes within the *Metarhizium* genus. To validate some of these BGCs, we analyzed RNA-seq data from *M. anisopliae* grown on cattle-tick (*Rhipicephalus microplus*) cuticles to evaluate their differential expression. In addition, we selected three up-regulated BGCs (Ma-PKS1, MaNRPS-PKS2, and MaTERP1) and applied phylogeny and comparative genomic analyses to predict their metabolic pathways and evolutionary history.

## Methods

### Genomes and RNA-seq data

All fungal genomes were downloaded from the NCBI Genome Database, and the descriptions and accession numbers are displayed in Additional file 1. For RNA-seq experiments, briefly, cattle tick *R. microplus* cuticles were sterilized and used as the sole carbon source for *M. anisopliae* E6 growth. Spore suspensions (5 × 10^6^ spores per ml) were used to inoculate the cuticles by immersion for 30 s. The inoculated cuticles were dispersed over 1 % water agar plates and maintained for 48 h (48hI) and 144 h (144hI) at 28 °C. As a control, the fungus was cultivated in 100 mL of liquid Cove’s complete medium (MCc) for 48 h (48hC) at 28 °C. The detailed RNA-seq experimental procedure, sequencing and data management have been previously described [32], and sequencing data are available under accession number PRJNA257269.

### Normalization and expression analysis

For expression analysis, we considered RPKM values > = 2 to indicate detectable expression. Genes were considered differentially expressed if the corresponding log2-fold change ratios were > = 1 or = < −1, with a 5 % false discovery rate (FDR < =0.05) [32].

### BGCs and related gene predictions

Putative BGCs in the *M. anisopliae* genome were identified with the antiSMASH 3.0 [33], SMURF [34], and SMIPS [35] algorithms and previous results from the literature were also examined [31, 36, 37]. The borders of each cluster were initially detected based on the antiSMASH 3.0 prediction [33] and subsequently confirmed with CASSIS, which assumes the existence of common regulatory patterns in cluster promoters for cluster delimitation [35]. The conservation of predicted clusters among *Metarhizium* spp. was assessed with MultiGeneBlast [38], based primarily on backbone gene conservation (*e*-value < 1 × 10^−5^, query coverage > 60 % and identity > 60 %). Afterward, BLASTP (non-redundant protein sequences database, recovering the best 500 hits) was used to search and curate orthologous clusters among other filamentous fungi genomes, and to select putative orthologous backbone genes for the phylogenetic analysis of MaPKS1, MaNRPS-PKS2, and MaTERP1 (*e*-value < 1 × 10^−5^, query coverage > 50 and identity > 45 %, ignoring more than one sequence under the same species) [39]. To further confirm that the collected genes were truly orthologous, the backbone genes of MaPKS1, MaNRPS-PKS2, and MaTERP1 were blasted against the MetaPhOrs database [40], and complete genomes were subjected to OrthoMCL curation, a Markov-based algorithm (clustering thresholds: e-value < 1e-05 and identity > = 30 %) (Additional file 2) [41]. Forty species with complete annotated genomes representing each taxon shown in this study were selected for OrthoMCL analysis (Additional file 1). Additionally, several fungal genomes from the Clavicipitaceae family were deposited at NCBI as raw or incomplete assemblies from projects that generally employed whole genome shotgun (WGS) strategies [42–44]. The contents of several unannotated genomes (*Epichlöe festucae*, *Balasia obtecta*, *Epichlöe baconii*, *Pochonia chlamydosporia*, *Periglandula ipomoeae*, *Claviceps fusiformis*, *Aciculosporium take*, *Epichlöe sylvatica*, *Neotyphodium gansuense*, *Hypocrella siamensis* and *Atkinsonella hypoxylon*) were accessed using BLASTN against the WGS database and MultiGeneBlast. The putative orthologous genes were annotated with FGENESH (gene-finding parameters for *Metarhizium* spp. or *Claviceps* spp.) and aligned with the backbone genes from *M. anisopliae* [45]. Genes that satisfied the previously fixed cutoffs were added to the phylogenetic analysis and cluster curation. Moreover, known global regulators that affect SM biosynthesis were also identified in *M. anisopliae* and their expression was evaluated.

### Phylogenetic analysis

A special procedure was adopted for the MaPKS1 phylogeny: given that the phylogeny of PKS genes can be chaotic, particularly for ortholog definition as it is difficult to differentiate orthologous from non-orthologous genes, we generated a tree that included collected entries comprising putative orthologs of MaPKS1, all PKS from *M. anisopliae* E6, and all characterized PKS from MIBiG, a database of characterized biosynthetic gene clusters [46]. An amino acid alignment was built using PRANK, and the evolutionary history was inferred using the Maximum Parsimony method with 1,000 bootstrap replicates and MrBayes [47–49] for 10^7^ generations (sampled every 100 steps), applying an average standard deviation of split frequencies < 0.01 as the convergence criterion. Parameters and trees obtained through the Bayesian approach were summarized by applying a 25 % burn-in (these trees are contained in Additional file 3) [50, 51].

After this confirmation, amino acid (BLAST collected entries for MaPKS1 and MaNRPS-PKS2) and nucleotide alignments (*tef1* gene, detailed below) were built and trimmed with GUIDANCE using PRANK as an MSA algorithm with default parameters [50, 52] to generate the phylogenetic analysis (these alignments are contained in Additional files 4, 5, 6). The best-fit evolutionary model for each alignment was assessed using Prottest 3.4 [53] for proteins and jmodeltest-2.1.9 [54] for nucleotides (Additional file 7). Phylogenetic trees were constructed using PhyML 3.1 [55] with 100 bootstrap replicates, and MrBayes with the same parameters as described above.

To generate the phylogeny of the putative helvolic acid cluster (MaTERP1; possibly obtained via HGT), protein sequences for each orthologous gene (excluding genes involved in fusidic acid biosynthesis; for a detailed explanation see Additional file 8) that belong to this cluster were recovered and processed as described above. An matrix representation parsimony (MRP) supertree was constructed based on the inferred trees with CLANN 3.1.3 with 100 bootstrap replicates [56]. Alternatively, the alignments were concatenated into a supermatrix using SeaView (supermatrix alignment is contained in Additional file 9) [57]. Evolutionary model analysis and phylogenetic inference for this supermatrix followed the procedures described above, and the tree was inferred using PhyML 3.1 (run with 1,000 bootstrap replicates). The cluster trees were rooted at species from the *Aspergillus* genus, and the topology was similar when the trees were unrooted or rooted at the midpoint. Both the supertree and the supermatrix tree were compared with the species tree to highlight possible HGT events implicated in the evolutionary history of this BGC [21, 58]. The species tree was based on the translation elongation factor 1-alpha (*tef1*) barcode and rooted at fungal species that did not belong to the Pezizomycotina class. The *tef1* gene is the current barcode pattern for species delimitation and classification in the *Metarhizium* genus and Clavicipitaceae family [59, 60]. The inferred species tree was analyzed manually for conflicts and incongruities with the current fungi and Clavicipitaceae evolutionary history [61, 62].

## Results

### BGC predictions and boundaries delimitation

The genome survey predicted 73 putative BGCs, comprising to twenty-two PKS, thirteen NRPS, nine terpenes (TERP), seven NRPS-PKS, three indoles (IND), two IND-NRPS, 1 IND-TERP, 1 TERP-PKS, 1 siderophore (SID), and fourteen BGCs, classified by antiSMASH as “OTHER”, a generic class of clusters encoding unusual BGCs (Additional file 10). Our survey found more BGCs than any other survey previously published for *Metarhizium* spp. [8, 31].

To refine the BGC boundaries, the predicted backbone genes were subjected to CASSIS, which assumes the presence of common regulatory patterns among genes from the same cluster. CASSIS was chosen because other tools only predict backbone genes (e.g., SMIPS), ignoring accessory genes, or overrating cluster boundaries (e.g., SMURF and antiSMASH). Based on the CASSIS prediction, 49 BGC boundaries were reassigned when compared to the previous antiSMASH prediction (Additional file 11; BGCs with reassigned boundaries are marked). However, the CASSIS results must be carefully analyzed, because other layers of regulation may be present, and some accessory genes may not exhibit the same regulation patterns found in the rest of the cluster [35].

### Conservation of BGCs in the *Metarhizium* genus and host range

The majority of BGCs (> 83 %) found in *M. anisopliae* are well conserved in host-generalists (*M. robertsii* and *M. brunneum*) and intermediate-host-range species (*M. guizhouense* and *M. majus*); including *M. robertsii* ARSEF23 (69 conserved clusters [cc]), *M. brunneum* ARSEF3297 (70 cc), *M. guizhouense* ARSEF977 (64 cc) and *M. majus* ARSEF297 (61 cc) (Additional file 10). Some SM clusters were also found to be conserved in host-specialist species, such as *M. acridum* CQMa102 (35 cc) and *M. album* ARSEF1941 (30 cc); however, this conservation was present to a lesser degree (Additional file 10).

### Comparative genomic analysis of BGCs and phylogeny

Comparative genomic analysis was employed to clarify the predictable final products of the assigned BGCs, integrating these data with BGCs previously characterized in *Metarhizium* spp. These comparisons revealed certain interesting clusters, which are listed in Additional file 10. MaPKS2 (MANI_004781) was predicted to be responsible for the biosynthesis of aurovertins, which are metabolites that have already been isolated from *Metarhizium* spp. cultures [63] but lack a characterized BGC. MaPKS2 exhibited 42–77 % identity with the BGC responsible for aurovertin biosynthesis in *C. arbuscula* (Additional file 12) [64]. MaTERP2 (MANI_002110) was assigned as a lanosterol cyclase, exhibiting 79 % identity with the partially characterized lanosterol cyclase from *Trichoderma harzianum* [65]. The final product of MaIND-NRPS1 (MANI_029655) was predicted to be an elymoclavine/ergovaline-related compound. This generic classification took into account the conservation between MaIND-NRPS1 and both elymoclavine and ergovaline characterized clusters (Additional file 12). MaIND-NRPS1 (MANI_029655) exhibited 57–77 % identity with a portion of the elymoclavine BGC from *C. fusiformis* [66]. Furthermore, the NRPS gene (MANI_029666) internal to the cluster exhibited 59 % identity with the biosynthetic ergovaline NRPS from *Neotyphodium lolii* [67] (Additional file 12). A generic classification was also applied for MaIND-TERP1 (MANI_011022) and MaNRPS-PKS3 (MANI_023437), which were designated as a terpendole E/lolitrem-related compound, and xenolozoyenone-related compound, respectively. MaIND-TERP1 exhibited 60–75 % identity with the biosynthetic terpendole E BGC characterized in *Chaunopycnis* (*Tolypocladium*) *alba* [68], and 59–77 % identity with the biosynthetic lolitrem BGC characterized in *E. festucae* [69, 70] (Additional file 12). However, MaIND-TERP1 cluster contains additional genes that are not conserved in the terpendole E and lolitrem clusters; these genes could potentially participate in the biosynthesis of the resulting terpendole E/lolitrem-related compound. MaNRPS-PKS3 exhibited 31–50 % identity with the xenolozoyenone BGC characterized in *Glarea lozoyensis* [71] (Additional file 12). Despite the low identity, both clusters were phylogenetically related as determined by Yue and coworkers (2015), further supporting our proposed assignment.

For the MaNRPS-PKS2 (MANI_018878), MaTERP1 (MANI_010527/MANI_010530/MANI_010531/MANI_010532), and MaPKS1 (MANI_014762) clusters, we deepened the comparative genomic analysis by performing a phylogeny. This phylogeny was performed because these three clusters are up-regulated during early infection (48hC x 48hI; following section), have a narrow cluster distribution among fungi (differing from MaTERP2, for example, which is also up-regulated in early infection, but is ubiquitous among Ascomycota), may have originated in *Metarhizium* spp. via HGT events and are located in singular genomic regions.

**Fig. 1:**
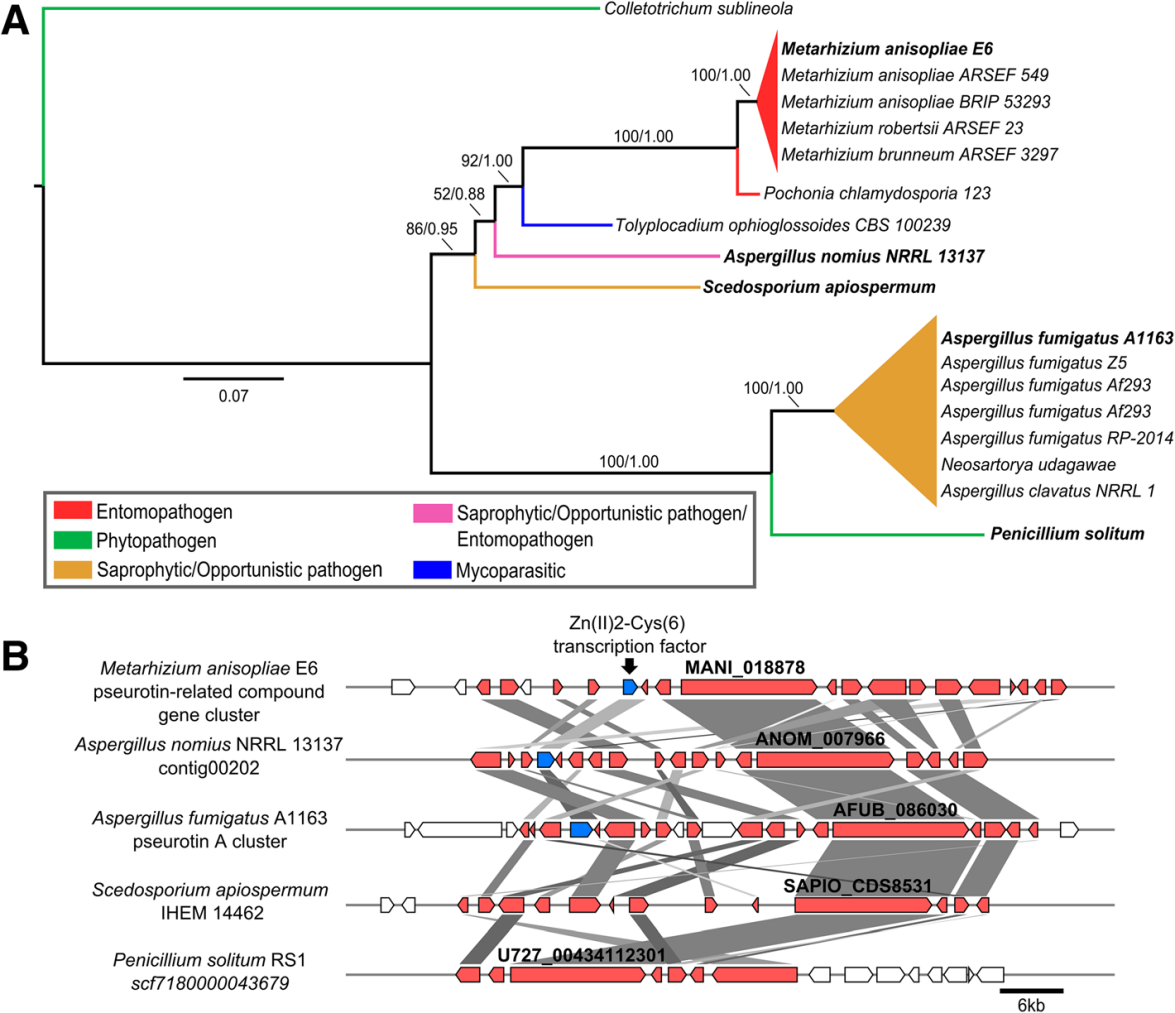
Pseurotin-related compound BGC (MaNRPS-PKS2). **a** Phylogenetic analysis was performed using Maximum-likelihood and Bayesian methods, based on the pseurotin-related backbone gene and orthologous sequences exhibited by several fungi. The orthologous sequences were classified according to fungal lifestyle trait, represented by different colors. The Bayesian tree is displayed, and branch support values (bootstrap proportions and Bayesian posterior probability) are associated with nodes. The Bayesian inference ran for 9,997,000 generations. Species in bold in (**a**) were used for the cluster conservation analysis presented in (**b**). **b** Some genes from *M. anisopliae* MaNRPS-PKS2 BGC resembled the characterized pseurotin BGC from *A. fumigatus* (34–81 % identity) and putative BGCs from *A. nomius* (49–85 % identity), *S. apiospermum* (63–84 % identity) and *P. solitum* (59–81 % identity). The *M. anisopliae* Zn(II) 2-Cys(6) transcription factor resembles the embedded transcription factor found in *A. fumigatus* (34 % identity), and the putative transcription factor from *A. nomius* (49 % identity). Interestingly, *S. apiospermum* and *P. solitum* do not have orthologs for this transcription factor. Orthologous genes were assigned the same color; white boxes represent genes that were not predicted to be part of *M. anisopliae* cluster, and blue boxes represent the conserved Zn(II)2-Cys(6) transcription factor

**Fig. 2:**
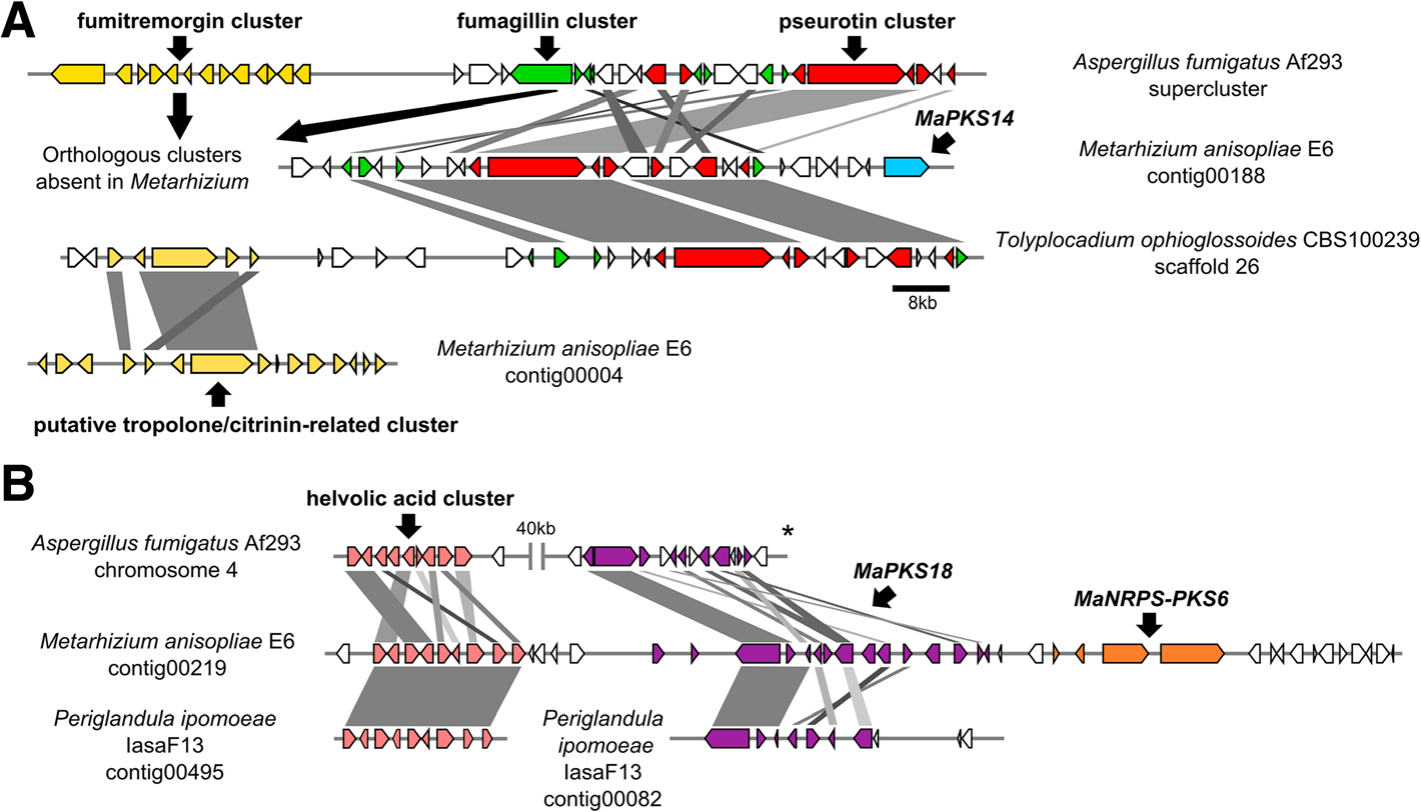
Conservation of supercluster regions in several species. **a** Comparison of the fumagillin/pseurotin supercluster region among *M. anisopliae*, *A. fumigatus* and *T. ophioglossoides*. The backbone gene from fumagillin (green) is absent in *M. anisopliae* and *T. ophioglossoides*, but some accessory genes are present and intertwined with the well-conserved pseurotin BGC (red). These accessory genes appear to participate in metabolite biosynthesis; therefore, the final product of this cluster was speculated to be a pseurotin-related compound. Upstream of the pseurotin-related BGC, the fumitremorgin cluster (yellow) is present in *A. fumigatus*, but absent in *M. anisopliae*, and there is a putative tropolone/citrinin-related BGC at this location in *T. ophioglossoides*. The tropolone/citrinin-related BGC has orthologous sequences (MaPKS1) in *M. anisopliae*, although they are located in a different genomic region. The MaPKS14 (light-blue) BGC is located downstream the pseurotin-related BGC only in *M. anisopliae*. **b** Comparison of a putative supercluster region in *M. anisopliae*, *A. fumigatus*, *A. niger*, and *P. ipomoeae*. Three BGCs (helvolic acid, MaPKS18, and MaNRPS-PKS6) were assigned to this *M. anisopliae* region. The helvolic acid (pink) and MaPKS18 (purple) clusters appear to be co-regulated. Additionally, both are conserved in *A. fumigatus* and *P. ipomoeae*, although the BGCs are distantly located in chromosome 4 in *A. fumigatus*. * This locus was inverted to fit in the figure

MaNRPS-PKS2 (MANI_018878) matched the characterized pseurotin BGC from *A. fumigatus* with considerable identity (63–81 %) (Fig. 1a and b) [72]. Furthermore, the search for orthologs and phylogenetic analysis revealed a restricted cluster distribution among filamentous fungi, with conservation observed only in host-generalist *Metarhizium* spp. (Fig. 1a). In *A. fumigatus*, this BGC is located in a singular genomic region with intertwined biosynthetic genes involved in the formation of fumitremorgin, fumagillin, and pseurotin. This region is under the control of the global regulator LaeA, and fumagillin and pseurotin are co-regulated by a supercluster-embedded regulatory gene [36]. In the genus *Metarhizium*, this BGC appears to have been horizontally acquired from an unknown donor, and fumitremorgin and fumagillin backbone genes are absent, although it is likely that some fumagillin accessory genes were also horizontally acquired (Fig. 2a). These accessory genes are strongly up-regulated, similar to the remaining pseurotin cluster (Table 2), indicating their likely participation in compound biosynthesis and leading us to classify the final product of this cluster as a pseurotin-related compound. Although CASSIS was unable to detect similar regulatory regions in the promoters of the pseurotin-related compound BGC, the embedded Zn(II) 2-Cys(6) transcription factor highlighted in *A. fumigatus* is conserved in *M. anisopliae* (MANI_018928; 34 % identity). This transcription factor can regulate the entire cluster and genes in the vicinity, analogous to the regulation that occurs in *A. fumigatus*. In addition, there is one other up-regulated backbone gene (MaPKS14; MANI_018879; Additional file 11) that is located near the pseurotin-related compound in *Metarhizium*, indicating possible co-regulation (Fig. 2a). Similarly, a pseurotin-related cluster located near an orthologous for MaPKS1 (although MaPKS1 is located in another genomic region in *Metarhizium* spp. genomes) was detected in *Tolypocladium ophioglossoides* (Fig. 2a). These results suggest that pseurotin and pseurotin-related compound clusters can be embedded in superclusters in different vicinities and configurations. Furthermore, these differences in pseurotin cluster location favor the proposed explanation that this cluster is located in highly variable regions in different genomes.

**Table 2:** Expression profiling of the *M. anisopliae* cluster related to the biosynthesis of a pseurotin-related compound

NCBI gene locus ID	Expression (RPKM)			Differential expression (log2-fold change)		Gene product
	48hC	48hI	144hI	48hCx48hI	48hIx144hI	
MANI_029058	0.00	0.34	0.34	NA	NA	Hypothetical protein
MANI_018942	1.75	0.00	5.75	NA	NA	Hypothetical protein
MANI_029071	0.00	209.91	134.67	9.77	ND	Integral membrane protein
MANI_018916	0.55	492.48	212.39	9.64	−1.28	Cytochrome P450
MANI_018955	1.03	37.39	59.92	5.02	ND	Hypothetical protein
MANI_018958	2.21	355.01	165.40	7.30	−1.10	Phytanoyl-CoA dioxygenase
MANI_018941	1.92	422.89	226.54	7.76	ND	Phytanoyl-CoA dioxygenase
MANI_018928	0.00	63.24	30.65	8.67	−1.06	C6 finger transcription factor
MANI_018959	1.84	986.40	323.46	8.90	−1.57	Hypothetical protein
MANI_018934	0.65	268.46	62.03	8.51	−2.11	Alpha/beta hydrolase
MANI_018878	0.44	736.13	182.13	10.78	−1.96	Hybrid NRPS/PKS enzyme
MANI_018952	0.00	1129.61	347.42	12.26	−1.68	Methyltransferase
MANI_029062	0.00	286.33	99.09	11.23	−1.50	Cytochrome P450
MANI_120428	31.50	117.64	50.96	2.02	−1.25	Methionine aminopeptidase
MANI_029068	109.68	117.21	86.21	ND	ND	Methionine aminopeptidase
MANI_018894	0.00	131.20	48.31	10.56	−1.38	Acetate-CoA ligase
MANI_111428	0.98	599.02	205.75	9.29	−1.51	Steroid monooxygenase
MANI_018962	0.00	269.62	67.44	9.55	−1.94	Hypothetical protein
MANI_018945	0.00	15.06	4.25	NA	NA	Methyltransferase
MANI_018943	0.00	296.14	80.09	10.19	−1.81	Glutathione S-transferase
MANI_018919	0.74	889.71	213.01	10.07	−2.08	O-methyltransferase

The relative changes in expression levels were estimated at 48 h for the control condition (hC) and both 48 and 144 h for infection conditions (hI). *NA* Not Available, *ND* No Difference

Given that supercluster arrangements are misleading when performing BGC predictions using search algorithms [36], we identified another putative supercluster in intermediate- and generalist-host-range *Metarhizium* spp. This putative supercluster is located in *M. anisopliae* contig 219, and is comprised of three clusters: MaPKS18 (MANI_010451), MaTERP1 (putatively enrolled in helvolic acid biosynthesis, as detailed below) and MaNRPS-PKS6 (MANI_010456/ MANI_121659) (Fig. 2b). This sequence region misleads the antiSMASH prediction, being the BGCs delimited by CASSIS and previous results from the literature. Furthermore, there is an apparent co-regulation of both the MaPKS18 and putative helvolic acid BGCs, which are up-regulated in early infection (48hC x 48hI) and down-regulated in late infection (48hI x 144hI), supporting the supercluster hypothesis (Table 3; Additional file 11). The putative helvolic acid (MaTERP1) and MaPKS18 BGCs have orthologs in *A. fumigatus* and *P. ipomoeae* (Fig. 2b). In *A. fumigatus* Af293, both clusters are located in the same chromosome and are separated by 40 kb, but the quality of the *P. ipomoeae* IasaF13 genome did not permit a synteny comparison. This set of results reinforces the notion that BGCs are located in rapidly evolving genomic regions and suggests that superclusters can be widespread in multiple fungal genomes.

**Table 3:** Expression profiling of the *M. anisopliae* cluster related to the biosynthesis of a helvolic acid compound

NCBI gene locus ID	Expression (RPKM)			Differential expression (log2-fold change)		Gene product
	48hC	48hI	144hI	48hCx48hI	48hIx144hI	
MANI_010527	0.54	13.59	2.67	4.51	−2.27	Cytochrome P450
MANI_010536	8.44	36.91	1.48	2.22	−4.50	Transferase family protein
MANI_010512	4.59	25.08	0.42	2.56	−5.58	FAD binding domain-containing
MANI_010537	3.03	30.78	4.47	3.42	−2.72	Transferase family protein
MANI_010532	0.00	12.57	0.91	6.73	−3.60	Cytochrome P450
MANI_010594	3.34	25.10	6.37	2.94	−1.90	3-oxoacyl-reductase 1
MANI_010495	9.06	87.20	9.34	3.44	−3.17	Squalene-hopene-cyclase
MANI_010530	11.58	48.40	11.28	2.21	−2.09	Cytochrome P450
MANI_010531	84.51	57.83	17.62	NA	−1.67	Cytochrome P450

The relative changes in expression levels were estimated at 48 h for the control condition (hC) and both 48 and 144 h for infection conditions (hI). *NA* Not Available, *ND* No Difference

**Fig. 3:**
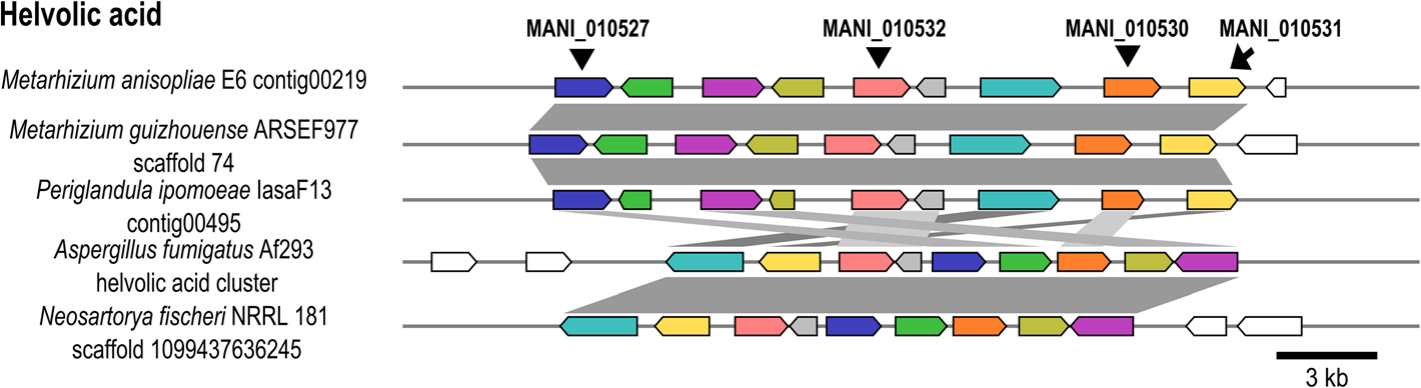
Putative helvolic acid (MaTERP1) conservation and synteny. The MaTERP1 cluster from *M. anisopliae* resembled the characterized helvolic acid cluster from *A. fumigatus* (41–65 % identity), and putative BGCs from *N. fischeri* (41–64 % identity) and *P. ipomoeae* (80–90 % identity). Notably, the BGC found in *P. ipomoeae* exhibits a strong synteny with clusters from the *Metarhizium* genus (e.g., *M. anisopliae* and *M. guizhouense*). The locus tags for the four backbone genes are given

The putative helvolic acid BGC (MaTERP1) showed considerable identity (41–65 %) with the helvolic acid cluster from *A. fumigatus* [37] (Fig. 3; Table 3). The helvolic acid cluster is organized around the prostadienol synthase gene AFU4G14770 in *A. fumigatus*. This cluster is unique, containing four backbone paralogous genes. As already suggested, this cluster evolved by gene duplication and differentiation from an ancestral monooxygenase, a transferase and two dehydrogenases [37]. This configuration is also observed in *M. anisopliae*, with four backbone genes (MANI_010527/MANI_010530/MANI_010531/MANI_010532). Furthermore, in addition to the backbone genes, all accessory genes highlighted in *A. fumigatus* are conserved in *M. anisopliae* (Fig. 3). Additionally, the isolation of helvolic acid from *Metarhizium* cultures supports the suggestion that MaTERP1 is responsible for metabolic biosynthesis [25].

**Fig. 4:**
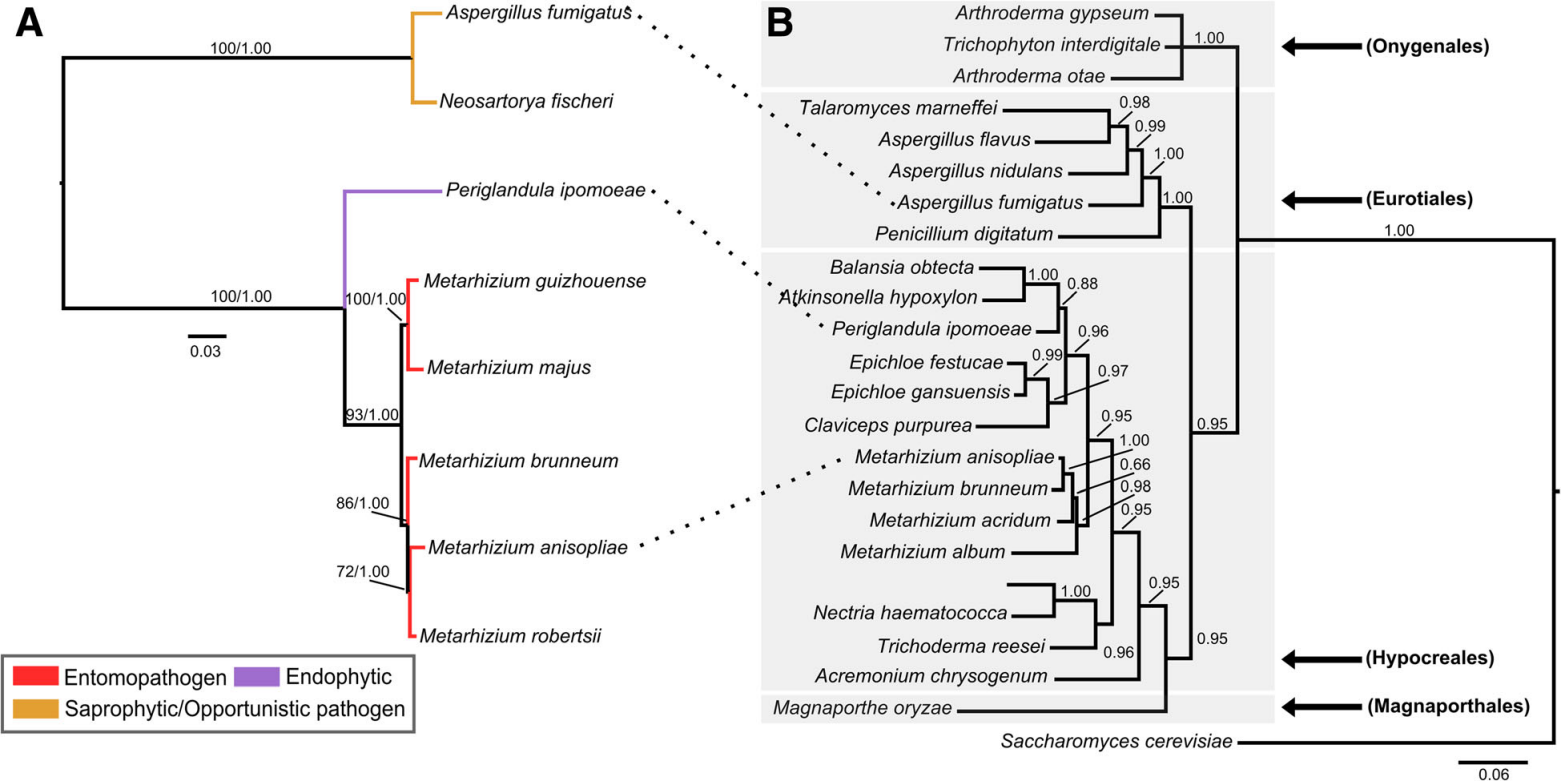
Species and helvolic acid BGC (MaTERP1) phylogeny. **a** Supermatrix tree of nine genes (MANI_010495/ MANI_010512/ MANI_010527/ MANI_010530/ MANI_010531/ MANI_010532/ MANI_010536/ MANI_010537/ MANI_010594) involved in helvolic acid biosynthesis. This supermatrix tree resembles the generated supertree (Additional file 13). The orthologous sequences were classified according to fungal lifestyle trait, represented by different colors. The Bayesian tree is displayed, and branch support values (bootstrap proportions and Bayesian posterior probability) are associated with nodes. The Bayesian inference ran for 1,000,000 generations. The cluster tree was compared with the species tree presented in (**b**). Note that the helvolic acid BGC is present in few Eurotiales and Hypocreales species. **b** The phylogeny of *tef1*, a barcode gene, showing established species relationships. Branch support values (Bayesian posterior probability) are associated with nodes. The Bayesian inference ran for 43,000 generations

Additionally, the phylogenetic analysis revealed a narrow cluster distribution (Fig. 4). This BGC is only found in intermediate- and generalist-host range *Metarhizium* spp., *P. ipomoeae* (Hypocreales order), and species from the *Aspergillus* genus (Eurotiales order), and is absent in *Metarhizium* host-specialist species and other members of the Clavicipitaceae family. The phylogenetic trees presented in this work, the strong gene conservation and uncommon cluster origin/formation, suggesting that this cluster may have been originated in *Metarhizium* species via an HGT event from a donor species closely related to the Eurotiales order (Fig. 4). This hypothesis is supported by the large evolutionary distance between Eurotiomycetes and Sordariomycetes (which diverged approximately 400 million years ago [MyA]) [73] and by the absence of a complete helvolic acid BGC in other species of the Hypocreales order (Fig. 3; Additional file 4).

**Fig. 5:**
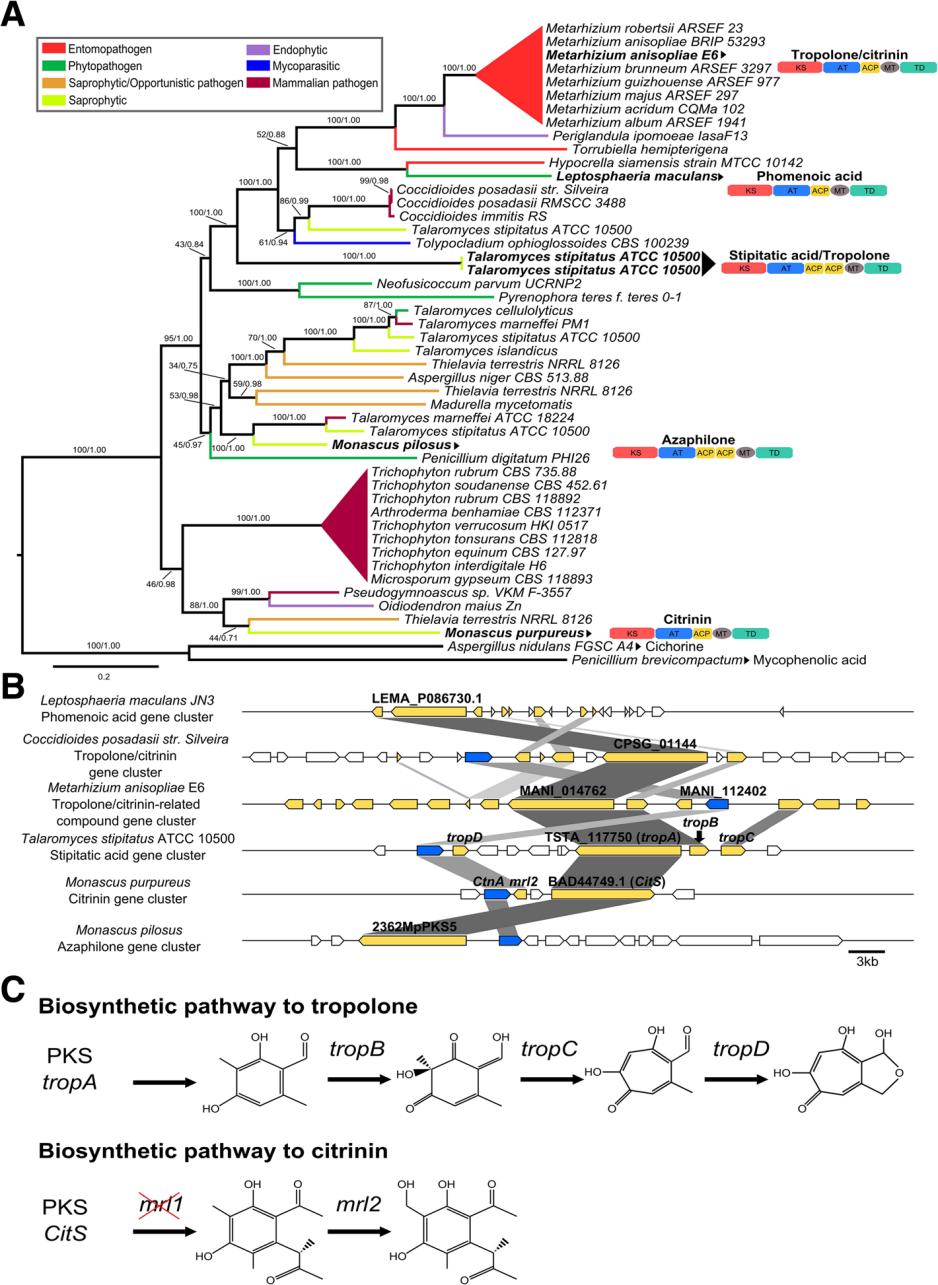
Tropolone/citrinin-related compound BGC (MaPKS1). **a** Phylogenetic analysis was performed using Maximum-likelihood and Bayesian methods, based on the tropolone/citrinin-related backbone gene and orthologous sequences in several fungi. Additionally, two PKS outgroup sequences were added: cichorine (*Aspergillus nidulans* FGSC A4) and mycophenolic acid (*Penicillium brevicompactum*). The orthologous sequences were classified according to fungal lifestyle trait, represented by different colors. The Bayesian tree is displayed, and branch support values (bootstrap proportions and Bayesian posterior probability) are associated with nodes. The Bayesian inference ran for 120,000 generations. Species in bold in (**a**) also have their domain organization shown with abbreviations (KS: Keto-synthase; AT: Acyltransferase; ACP: Acyl carrier protein; MT: Methyltransferase O- or C-; TD: Thioester reductase), and were used for the cluster conservation analysis presented in **b**. These clusters have characterized or partially characterized biosynthetic routes. **b** Some genes from *M. anisopliae* MaPKS1 BGC resembled the characterized stipitatic acid (tropolone) BGC from *T. stipitatus* and the citrinin BGC from *M. purpureus*. These conserved genes are involved in the first steps of the biosynthesis of their compound, as described in **c**. Note that the *mrl1* gene of the citrinin biosynthetic pathway is absent in *M. anisopliae*. Additionally, the gene MANI_112402 resembles the *ctnA* citrinin regulator from *M. purpureus* (59 % identity), and putative transcription factors from *C. posadasii*, *T. stipitatus*, *M. purpureus* and *M. pilosus* as demonstrated in (**b**). Orthologous genes were assigned the same color; white boxes represent genes that are not predicted to be part of *M. anisopliae* cluster; and blue boxes represent the conserved transcription factor

Another contribution of functional genomics coupled with phylogenetic analysis was the partial elucidation of the first steps in the biosynthesis of up-regulated MaPKS1. The *MaPKS1* backbone gene (MANI_014762) is orthologous to several characterized backbone genes: tropolone/stipitatic acid [74], citrinin [75], phomenoic acid [76] and azaphilone [77]. These orthologs suggest that a similar biosynthetic route is partially shared between MaPKS1 and these characterized metabolites (Fig. 5a e b). The conserved genes between MaPKS1 and the tropolone/stipitatic acid route are involved in the first steps of metabolic biosynthesis. The stipitatic acid backbone gene *tropA* exhibits 46 % identity with the *MaPKS1* backbone gene (MANI_014762), *tropB* exhibits 47 % identity with MANI_014847, *tropC* exhibits 60 % identity with MANI_112407 and *tropD* exhibits 64 % identity with MANI_014887 (Fig. 5c). Similarly, two genes conserved between MaPKS1 and the citrinin biosynthetic route are also involved in the first steps of metabolic biosynthesis. The citrinin backbone gene *CitS* exhibits 45 % identity with the *MaPKS1* backbone gene (MANI_014762), and *mrl2* exhibits 37 % identity with MANI_014887, although *mrl1* is absent (Fig. 5c). The potential conservation of these first metabolic steps supports the hypothesis that the final product of this BGC has, at minimum, the same biosynthetic origin and is related to tropolones and citrinins. However, the CASSIS prediction delineates a BGC comprised of 15 genes (several genes are not conserved in the tropolone or citrinin routes), leading us to classify the product of this cluster as a potential and generic tropolone/citrinin-related compound.

Our analysis also suggested that the self-transcription factor (MANI_112402) is involved in MaPKS1 regulation (Table 4). This gene exhibits strong expression (>6-fold difference with *P* < 0.05) and 59 % identity with the transcription factor *ctnA* from the *M. purpureus* citrinin cluster (Fig. 5b). In *M. purpureus*, the deletion of *ctnA* caused a large decrease in citrinin production [78]. Moreover, a similar gene is also conserved in the *Coccidioides posadasii* putative tropolone/citrinin BGC, in the *T. stipitatus* tropolone BGC, and in the *Monascus pilosus* azaphilone BGC, suggesting a similar, widespread regulatory strategy among these BGCs (Fig. 5b; genes marked in blue). It would be simple to characterize this BGC in *Metarhizium* by constructing a knockout strain for this gene.

**Table 4:** Expression profiling of the *M. anisopliae* cluster related to the biosynthesis of a tropolone/citrinin-related compound

NCBI gene locus ID	Expression (RPKM)			Differential expression (log2-fold change)		Gene product
	48hC	48hI	144hI	48hCx48hI	48hIx144hI	
MANI_014850	37.70	8.46	12.14	−1.95	ND	Mercuric reductase
MANI_014940	0.00	13.82	33.93	ND	1.36	Hypothetical protein
MANI_014941	0.00	0.00	1.19	NA	NA	Hypothetical protein
MANI_014846	0.55	3.11	0.00	NA	NA	Major Facilitator Superfamily protein
MANI_014815	1.74	1.05	0.71	NA	NA	Hypothetical protein
MANI_028157	23.92	1.45	0.98	−3.76	ND	Pantothenate transporter
MANI_014957	2.35	70.02	49.98	4.76	ND	YCII-domain protein
MANI_014867	21.19	3.01	4.08	−2.63	ND	Major Facilitator Superfamily protein
MANI_014762	0.56	29.70	34.13	5.77	ND	Polyketide synthase
MANI_014847	2.36	8.57	32.88	ND	1.92	Salicylate 1-monooxygenase
MANI_014887	0.00	86.51	86.40	8.80	ND	Leucoanthocyanidin Dioxygenase
MANI_112402	0.00	9.21	8.88	6.25	ND	Citrinin biosynthesis transcriptional activator CtnR
MANI_112407	0.57	17.13	28.22	4.71	ND	Cytochrome P450
MANI_014818	1.86	23.25	28.56	3.68	ND	Siderophore iron transporter
MANI_014903	0.00	56.20	81.77	8.09	ND	Nucleoside-diphosphate-sugar epimerase

The relative changes in expression levels were estimated at 48 h for the control condition (hC) and both 48 and 144 h for infection conditions (hI). *NA* Not Available, *ND* No Difference

### Expression of BGCs

To validate some of our predictions regarding *M. anisopliae* BGCs, we conducted RNA-seq analysis using a system mimicking host infection. The fungus was cultured in Cove’s Complete Medium (C: Control condition) and in the presence of tick cuticles (I: Infection condition) for 48 or 144 h, as described in the Materials and Methods. Two pairwise comparisons were performed: 48hC x 48hI (early infection conditions) and 48hI x 144hI (late infection conditions). Nearly half of the predicted SM clusters, specifically 49 (36/73), were expressed (RPKM > = 2) under the analyzed culture conditions, and 20 % (15/73) were up-regulated under early infection conditions (48hI x 48hC), highlighting their potential relevance in the initial steps of infection. Conversely, 7 % (5/73) of the predicted BGCs were down-regulated when comparing 48hI versus 48hC. No up-regulated cluster was detected when 48hI was compared with 144hI; however, 14 BGCs were down-regulated. Moreover, of those 14 down-regulated BGCs, 9 were up-regulated under early infection conditions (Additional file 10).

The 15 BGCs up-regulated under early infection conditions included the already-cited tropolone/citrinin-related compound BGC (MaPKS1) (Table 4), the pseurotin-related compound BGC (MaNRPS-PKS2) (Table 2), the lanosterol cyclase BGC (MaTERP1), and the putative helvolic acid BGC (MaTERP2) (Table 3), in addition to the already-characterized destruxin (MaNRPS1) (Table 5), NG39x (MaNRPS-PKS1) (Table 6) and ferricrocin (MaNRPS9) (Additional file 11) clusters; Additionally, there were eight putative clusters of uncharacterized products (MaNRPS7; MaPKS14; MaPKS17; MaPKS18; MaIND1; MaOTHER8; MaOTHER12; MaOTHER13) (Additional file 11).

**Table 5:** Expression profiling of the *M. anisopliae* cluster related to destruxin biosynthesis

NCBI gene locus ID	Expression (RPKM)			Differential expression (log2-fold change)		Gene product
	48hC	48hI	144hI	48hCx48hI	48hIx144hI	
MANI_024443	2.67	138.02	14.72	5.80	−3.14	ABC multidrug transporter
MANI_024450	2.78	88.43	8.54	5.06	−3.34	Hypothetical protein
MANI_131037	0.00	119.36	8.75	9.88	−3.71	Glutamate decarboxylase
MANI_130923	3.06	100.47	14.43	5.11	−2.80	Aldo-keto reductase
MANI_024448	4.58	480.60	52.47	6.81	−3.08	Cytochrome P450
MANI_024437	9.41	261.50	15.65	4.92	−3.96	Destruxin synthetase

The relative changes in expression levels were estimated at 48 h for the control condition (hC) and both 48 and 144 h for infection conditions (hI). *NA* Not Available, *ND* No Difference

**Table 6:** Expression profiling of the *M. anisopliae* cluster related to NG39x biosynthesis

NCBI gene locus ID	Expression (RPKM)			Differential expression (log2-fold change)		Gene product
	48hC	48hI	144hI	48hCx48hI	48hIx144hI	
MANI_020814	6.73	6.92	7.65	ND	ND	Monophenol monooxygenase
MANI_020801	0.48	0.00	0.00	NA	NA	Ankyrin repeat protein
MANI_020903	0.00	1.63	0.00	NA	NA	Eukaryotic aspartyl protease
MANI_020934	0.00	2.15	0.00	NA	NA	eEF-1B gamma subunit-like protein
MANI_020906	0.00	2.23	0.00	NA	NA	Alpha/beta hydrolase
MANI_020791	0.00	14.72	0.37	9.80	−5.22	Hybrid PKS-NRPS protein
MANI_020948	6.83	45.86	3.73	ND	ND	Integral membrane protein
MANI_020870	4.31	19.98	2.65	2.24	−2.81	Major facilitator superfamily protein
MANI_121064	0.00	6.23	0.53	NA	NA	Hypothetical protein
MANI_020865	2.30	13.40	1.88	2.58	−2.72	P450 monooxygenase
MANI_020911	5.66	7.82	5.96	ND	ND	Carboxyl methyltransferase
MANI_121074	3.99	11.79	6.53	1.65	ND	Hypothetical protein

The relative changes in expression levels were estimated at 48 h for the control condition (hC) and both 48 and 144 h for infection conditions (hI). *NA* Not Available, *ND* No Difference

The five down-regulated BGCs under early infection conditions included: the xenolozoyenone-related compound (MaNRPS-PKS3) [71] and four putative clusters of uncharacterized products (MaNRPS8; MaNRPS10; MaOTHER1; MaOTHER11) (Additional file 11). The fourteen down-regulated BGCs under late infection conditions included the destruxin (MaNRPS1), serinocyclin (MaNRPS2), NG39x (MaNRPS-PKS1), helvolic acid (MaTERP1), xenolozoyenone-related compound (MaNRPS-PKS3), and pseurotin-related compound (MaNRPS-PKS2) BGCs, in addition to six putative clusters of uncharacterized products (MaNRPS11; MaPKS10; MaPKS14; MaPKS17; MaPKS18; MaTERP9; MaIND1; MaOTHER12) (Tables 2;3;5;6 and Additional file 11). Together, 30 % (22/73) of the predicted BGCs were differentially expressed in at least one of the pairwise comparisons.

### Expression of global regulators of fungal traits

Many BGCs contain self-transcription factors integrated into the cluster organization [35, 79], but global regulators of fungal traits also influence the expression of BGCs [80, 81]. These global regulators extend from single transcription factors and histone-modifying enzymes (e.g., CreA, PacC, StuA, nscC, AreA, AreB, MeaB, GcnE and hdaA) to protein complexes (e.g., Velvet, and CCAAT-binding complexes) [80–85]. Our RNA-seq analysis detected the expression of all of the aforementioned global regulators at significant levels (RPKM > = 2) (Table 7).

**Table 7:** Expression profiling of the *M. anisopliae* global regulators of fungal traits

Global regulator	Metabolism	NCBI gene locus ID	Expression (RPKM)			Differential expression (log2-fold change)	
			48hC	48hI	144hI	48hCx48hI	48hIx144hI
Velvet Complex							
VeA	Light	MANI_008143	30.53	71.74	45.95	1.32	ND
VelB		MANI_013601	25.91	29.21	18.58	ND	ND
LaeA		MANI_030399	3.64	6.84	4.97	NA	NA
CCAT-binding complex							
HapB	Iron	MANI_001834	38.29	29.90	55.14	ND	ND
HapC		MANI_119196	23.11	27.92	41.43	ND	ND
HapE		MANI_018323	41.24	22.68	30.49	ND	ND
HapX		MANI_009173	30.15	16.19	19.09	ND	ND
PacC	pH	MANI_008549	20.37	69.23	100.18	1.88	ND
CreA	Carbon	MANI_015776	9.31	65.57	70.54	2.91	ND
AreA	Nitrogen	MANI_016951	17.45	34.12	39.49	1.07	ND
AreB		MANI_028387	22.46	15.89	28.44	ND	ND
MeaB		MANI_019405	40.46	54.10	50.67	ND	ND
StuA	Sporulation	MANI_024223	38.24	75.85	92.09	1.02	ND
GcnE	Chromatin remodeling	MANI_000421	13.86	13.51	15.54	ND	ND
hdaE		MANI_025732	17.80	17.52	14.26	ND	ND
nsdC	Asexual development	MANI_013461	9.74	20.40	18.76	1.19	ND

The relative changes in expression levels were estimated at 48 h for the control condition (hC) and both 48 and 144 h for infection conditions (hI). *NA* Not Available, *ND* No Difference

Under early infection condition, global regulators linked to carbon (CreA) and nitrogen (AreA) metabolism, pH (PacC), light stimuli (VeA), asexual development (nsdC) and sporulation (StuA) were up-regulated (Table 7). Conversely, the best characterized global regulator, the LaeA methyltransferase, demonstrated lower expression. Additionally, global regulators linked to iron (HapB; HapC; HapE; HapX) and nitrogen (AreB; MeaB) metabolism, light stimuli (VeB), the chromatic remodeling histone acetyltransferase (GcnE) and deacetylase (hdaA), did not exhibit significant changes in gene expression (Table 7). In addition to demonstrating that many of these global regulators are active (a significant amount remain unexplored in the *Metarhizium* genus), expression analysis provides clues regarding how infection conditions can be regulated and which genes may perform this regulation.

## Discussion

Although a vast number of interesting metabolites have been isolated from *Metarhizium* cultures, little is known regarding how BGCs are organized, expressed and regulated as well as their potential functions during host infection and their evolutionary history. To this end, a deep survey of BGCs was performed in the *M. anisopliae* genome and combined with a transcriptional profile analysis from an infection model in the economically important cattle-tick *R. microplus*, which is responsible for a variety of livestock infections and is a promising candidate for biological control by *Metarhizium* [86–89].

Our survey predicted and delimited 73 BGCs, of which 20 % were up-regulated under early infection conditions (48hC x 48hI), and a subset of these (9 out of 15) were down-regulated under late infection conditions (48hI x 144hI) (Additional file 10). These results point to a drastic change between the metabolic profiles of early and late infection. The *Metarhizium* infection process is dynamic and may end in arthropod or fungal death [90]. Once the fungus adheres to a host, rapid morphological and transcriptional changes occur, including the expression of several virulence factors [91]. Ment and coworkers (2012) showed that after 3 or 4 days of attachment to a suitable host, *Metarhizium* kills the host [90], switching to a saprophytic state, and virulence determinant expression is attenuated [2]. Conversely, in an unsuccessful infection scenario, the fungus will exhaust the endogenous spore nutrient reserves at 3 or 4 days post-cuticle adhesion while attempting to circumvent host defenses, resulting in the demise of the pathogen [90]. We suggest that some of the identified up-regulated BGCs participate in the first scenario (successful infection), given that this hypothesis corroborated with the observed strong expression of the destruxin cluster, a well-known virulence factor [92], and ferricrocin [30]. Additionally, we hypothesize that other BGCs are also expressed during early and late infection, such as BGCs for the production of antifungal and antibacterial compounds that help the fungus to circumvent competition with opportunistic and symbiotic microorganisms. These BGCs may be induced by direct interactions between *M. anisopliae* and other microorganisms, independent of the host interaction. Moreover, our experimental design employing tick cuticles may have blocked the expression of these antibiotic BGCs, as the natural tick gut microbiota was excluded [32]. Indeed, the antibacterial viridicatumtoxin BGC (MaPKS9) proposed by Gibson and coworkers (2014) was silent under our mimicked infection conditions, supporting this notion (Additional file 11) [31].

Some BGCs that were previously characterized in *Metarhizium* were silent or did not alter their expression under at least one of the tested conditions. This was the case for MaNRPS2 BGC (serinoclycin; MANI_020119), which was down-regulated under late infection conditions, and MaNRPS8 (metachelin; MANI_003049), which was down-regulated under early infection conditions. The MaPKS8 (MrPKS2; MANI_028434), already-cited MaPKS9, (viridicatumtoxin; MANI_003768) and MaPKS20 (MrPKS1; MANI_122426) BGCs did not demonstrate detectable expression. The reduced participation of these BGCs in the infection process is expected in accordance with previous literature [26, 29–31].

Similarly, the up-regulation of the destruxin cluster under early infection conditions was in accordance with the described insecticidal effects and phenotypic analysis of destruxin mutants. Similarly, the up-regulation of the ferricrocin cluster highlights the already-described importance of this siderophore in the *Metarhizium* lifestyle and infection process [30]. However, while destruxin metabolites directly affect the host defenses [92], the reduced infection resulting from the absence of ferricrocin is linked to delayed germination and alterations in endogenous fungal iron content [30].

Some putative clusters highlighted by the comparative genomic analysis do not appear to affect the virulence of *Metarhizium* species (Additional files 10 and 11). Such is the case for the putative BGCs for aurovertin (MaPKS2; MANI_004781), elymoclavine/ergovaline-related compound (MaIND-NRPS1; MANI_029655) and terpendole E/lolitrem-related compound (MaIND-TERP1; MANI_011022). Aurovertin metabolites have been isolated from *M. anisopliae*, *P. chlamydosporia*, and *C. arbuscula* [63, 64, 93]. These compounds exhibit potent inhibition of adenosine triphosphate synthase [64], and aurovertin D, which was isolated from *P. chlamydosporia*, induced the death of the free-living nematode *Panagrellus redivivus* [93]. The non-expression (RPKM < 2, under the three conditions) of this cluster is intriguing, particularly in light of the reports regarding *P. chlamydosporia*, a species closely related to the *Metarhizium* genus. Similarly, elymoclavine, ergovaline, terpendole E and lolitrem are ergot alkaloids produced by fungi from the *Claviceps*, *Epichlöe* and *Tolypocladium* genera, which are closely related to the *Metarhizium* genus [66, 67]. Ergot alkaloids are potent toxic alkaloids whose intake can lead to several effects ranging from poor weight gain to gangrene and death [67]. Similar to aurovertin, it could be postulated that these metabolites play a role in infection, although our results suggested the opposite. Certainly, the construction of gene knockouts for these BGCs will help to understand their importance in the *Metarhizium* lifestyle and interactions.

The xenolozoyenone-related compound (MaNRPS-PKS3; MANI_023437) was another cluster that was down-regulated, indicating decreased participation in the infection process. Xenolozoyenone is a pyrrolidinedione-containing compound isolated from *G. lozoyensis*. Although no biological activity or possible function has been linked to this compound, the backbone gene from this cluster (*glpks3*-*glnrps7*) was the first described classical fungal protein-coding operon [71]. The conservation of orthologs of these genes highlights the possibility that protein-coding operons are widespread among fungal genomes and BGCs and represent a new and virtually unexplored level of regulation.

The results of the transcriptomic analysis may also help to redefine the importance of NG39x (MaNRPS-PKS1; MANI_020791) in the infection process. The RNA-seq results suggested that the NG39x BGC might play a role in infection, as demonstrated by the differential expression (> 9.5-fold difference with *P* < 0.05 for the backbone gene MANI_020791) observed under early infection conditions (Table 6). Although NG39x cluster expression has been observed in vivo [27], a previous study performed RT-PCR analysis to evaluate the transcripts of the NG39x cluster while comparing in vitro fungal growth with growth in infected *S. exigua* larvae and did not revealed clear differences between the two conditions. It was postulated that the expression of the NG39x cluster is developmentally regulated, given that there was an increase in BGC expression related to biomass augmentation. Additionally, the knockout mutant for the backbone gene responsible for NG39x biosynthesis did not lead to diminished fungal virulence, indicating minor or no participation in the insect infection process [27]. NG39x compounds were recently reported to exert antiproliferative effects in human cell cultures, via a mechanism that involves impairment of the integrity of nucleic acid biosynthesis. However, it has not been established if these compounds directly interact with DNA or RNA or interact with some protein [94]. Therefore, it is possible that the toxicity of NG39x compounds varies according to the host and may exemplify cases of host specificity, which explains the increased expression of this cluster under early infection condition.

We expanded the comparative genomic analysis by constructing a phylogeny to investigate the metabolic pathways of three interesting up-regulated clusters: a pseurotin-related compound BGC (MaNRPS-PKS2), a putative helvolic acid BGC (MaTERP1) and a tropolone/citrinin-related compound BGC (MaPKS1).

Pseurotins are a group of compounds containing phenylalanine coupled to a polyketide with a spiro ring structure, and have been isolated from *Aspergillus* spp. and *Pseudeurotium ovalis* cultures [95]. Anti-angiogenic activities as well as IgE and chitin synthase inhibitory activities have been reported for pseurotins [96]. Although this metabolite has never been isolated from *Metarhizium* cultures, a recent report revealed a putative pseurotin cluster in *M. robertsii* [36] and served as the starting point for analysis. The expression and comparative genomic analysis suggested that the final product of this cluster is not pseurotin, but a related compound. Wiemann and coworkers (2013) speculate that the product of this BGC could be 12-hydroxy-ovalicin (Mer-f3). Mer-f3 exhibits inhibitory and immunosuppressive activities against certain tumor cell lineages, and ovalicin-related compounds isolated from *M. anisopliae* cultures have been tested for the treatment of atopic dermatitis in mice [97, 98].

The pseurotin-related compound BGC from *M. anisopliae* may also be embedded in a supercluster, analogous to the pseurotin BGC from *Aspergillus* species. This macro regulation exerted by a supercluster is an interesting and virtually unexplored area of research. Comparative genomic analysis and the transcription profile suggested the presence of another supercluster in *M. anisopliae*. In view of the apparent co-regulation of MaPKS18 and the putative helvolic acid BGC (MaTERP1) as well as the fact that the sequence region containing both clusters misled the antiSMASH prediction we suggest that this region is a supercluster. However, more investigation is needed to prove this hypothesis.

The aforementioned helvolic acid is another interesting secondary metabolite. Helvolic acid is a well-known fusadine triterpene antibiotic that is active against Gram-positive bacteria [11]. In *A. fumigatus*, this SM is suggested to play an important role in human pathogenesis, exerting inhibitory effects on macrophages and inducing epithelial damage [99]. This compound also exhibits antifungal activity against phytopathogens and demonstrates antifeedant properties in the armyworm *Mythimna separata* [100]. Our phylogenetic analysis supports the notion that this cluster was horizontally acquired from a species closely related to Eurotiales, given that this BGC is restricted to only a few species. Moreover, not only helvolic acid but its derivative (1,2-dihydrohelvolic acid) have been isolated from *M. anisopliae* cultures. Furthermore, 1,2-dihydrohelvolic acid was isolated from fungus grown in insect-derived material, suggesting that this cluster is active under infection conditions [25]. Conversely, purified helvolic acid from *M. anisopliae* cultures does not appear to be toxic to some insects [101]. Although expression analysis suggested a role in infection and antifeedant properties for this metabolite have been reported in the literature, the true importance of helvolic acid in the *Metarhizium* lifestyle must be assessed.

Our analysis also suggested that the final product of MaPKS1 is a tropolone/citrinin-related compound. Tropolones and citrinins are structurally very similar and are chemically grouped by the local suffering oxidation [74, 75]. Furthermore, MaPKS1 contains several genes that are not conserved in the characterized tropolone/stipitatic acid and citrinin biosynthetic routes. Thus, while some genes in the MaPKS1 BGC are conserved in the tropolone and citrinin routes, and these genes likely perform similar functions in *M. anisopliae*, the final metabolic function of this cluster is still unknown. Better classification of the final product of this BGC will emerge with the functional analysis of this cluster.

Our analysis also uncovered global regulators of fungal development, nutrition and niche adaptation that are up-regulated under early infection conditions and can govern the expression of both up- and down-regulated BGCs, as observed in other fungal species [82]. The up-regulation of PacC under early infection conditions (>1.88-fold difference with *P* < 0.05; MANI_008549) is in agreement with the ability of *M. anisopliae* to modulate pH during the infection process by alkalizing infected cuticles [102, 103]; under alkaline pH conditions, PacC serves as a positive regulator promoting the transcription of alkaline-expressed genes, and it has been suggested that PacC simultaneously represses acid-expressed genes [104]. The importance of PacC in *Metarhizium* infection and fungal growth has already been established via the construction of knockouts [105]. However, it has not been determined how this gene influences secondary metabolism in *Metarhizium* spp. PacC may potentially influence the BGC for destruxin production, as it was verified that destruxin production is favored by an alkaline pH [106]. In accordance with the central role of PacC in secondary metabolism regulation, there have been reports in *Aspergillus* spp., indicating that this gene regulates penicillin and sterigmatocystin BGCs [107, 108]. Another gene linked to secondary metabolism regulation is the carbon catabolite repressor (CreA), which is also up-regulated under early infection conditions (> 2.91-fold difference with *P* < 0.05; MANI_015776). CreA is a global repressor that ensures the utilization of preferred carbon sources, preventing the expression of genes linked to assimilatory traits of non-preferred carbon sources [109]. CreA is known to negatively influence the production of penicillin at high carbon concentrations and was suggested to act in ochratoxin A regulation [82, 110]; however, it still must be determined how this gene influences *Metarhizium* spp. secondary metabolism. Interestingly, in filamentous fungi, carbon and pH metabolisms appear to be related [111]. Recently, Bi and coworkers (2015) showed that many organisms acidify media under conditions of carbon excess, while alkalization occurs under carbon deprivation. Mutants for glutamate dehydrogenase 2 (*gdh2*), which catalyzes the deamination of non-preferred carbon sources, resulting in ammonia production, exhibited reduced virulence and alkalization potential, and *gdh2* expression was negatively correlated with CreA in *Colletotrichum gloeosporioides*, *Penicillium expansum*, *Aspergillus nidulans*, and *Fusarium oxysporum* [111]. This similar regulation in distantly related species (e.g., *Fusarium* spp. and *Aspergillus* spp.) suggests a widespread form of regulation that can be present in *Metarhizium* species. However, our observation of both PacC and CreA up-regulation is contrary to this notion. The positive regulation of these global regulators under early infection conditions may indicate a switch from infection to a saprophytic state, which could lead to the down-regulation of BGCs involved in infection. Notably, the orthologs for the *laeA* gene in *M. anisopliae* exhibited lower expression (difference not available under both early and late infection conditions with *P* < 0.05; MANI_030399) (Table 7). LaeA demonstrates central importance in SM metabolism and virulence in *Aspergillus* spp. and *Fusarium* spp. [112, 113]. In *Aspergillus carbonarius*, the deletion of *laeA* led to a drastic decrease in ochratoxin A production [114], and the deletion of this gene in *A. fumigatus* blocks the expression of sterigmatocystin, penicillin, lovastatin and helvolic acid BGCs [115]. Future studies should be performed to assess the role of *laeA* in the *Metarhizium* lifestyle and virulence.

Finally, the conservation of the BGCs found among *Metarhizium* species is yet another fundamental topic. The absence of the destruxin BGC in the host-specialists *M. acridum* and *M. album* led to the conclusion that this SM could play a pivotal role in the host-generalist lifestyle [28]. The comparative genomic analyses of BGCs in the *Metarhizium* genus indicate that only one half of these BGCs are conserved in host-specialist species. In addition to this, host-specialist species also encode BGCs that not conserved in host-generalist species, which represents an important difference in the metabolic profile and niche adaptation potential of these species. Entomopathogenic fungi may retain some BGCs related to the infection process, and SM acquisition can act as a driving force towards host generalization. Accordingly, our results showed that only 6 out of the 15 up-regulated clusters are conserved in *M. acridum* and *M. album* (Additional file 10). The results suggest that the plethora of SM produced by host-generalist and host-intermediate species exert an underestimated impact in the infection process, and destruxins as well as many other SMs may be essential for fungal adaptation to new hosts.

## Conclusions

Although considerable progress has been made in understanding the *Metarhizium* infection process, unanswered questions remain, in particular those related to the definition of host specificity, which is a central and still not fully understood topic in the study of entomopathogenic fungi. Different fungal species, and even different strains, are able to synthesize different combinations of SM compounds that may result in distinct adaptation strategies [73]. In general, the results point to a fundamental role for SM in initial infection, with a notable difference in the BGCs present in host-specialist versus host-generalist *Metarhizium* species. Transcript analysis of samples obtained under conditions mimicking tick infection, showed the activation of several BGCs, with some up-regulated only during the early steps of infection. The conservation and expression of these genes can actively support *M. anisopliae* as a successful generalist pathogen. The origin and evolution of SM clusters is another interesting topic, and the results suggest that HGT events may have shaped the metabolic potential of generalist species, but this hypothesis still must be explored.

Several BGCs characterized by other groups, which were shown to participate in the infection process only to a minor degree, as well as several putative BGCs highlighted by this work were silent under mimicked conditions, reaffirming the hypothesis regarding their participation in the infection process. In this work, we took the first steps toward the characterization of several previously unexplored BGCs and examined their regulation, focusing on their potential participation in infection. These are important issues to investigate, not only to acquire basic knowledge regarding fungal lifestyles and adaptation, but also to explore for future biological control applications, and the identified genes and global regulators represent valuable targets for further experimental study.

## Additional file**s**

Additional file 1: Fungal genomes used in this work. (PDF 158 kb)
Additional file 2: OrthoMCL clustering results. (PDF 157 kb)
Additional file 3: Phylogenetic trees constructed with putative orthologs of MaPKS1, all PKS from *M. anisopliae* E6, and all characterized PKS from MIBiG. (PDF 600 kb)
Additional file 4: Amino acid alignment for the pseurotin-related backbone gene and related orthologs. (FASTA 67 kb)
Additional file 5: Amino acid alignment for the tropolone/citrinin-related backbone gene and related orthologs. (FASTA 125 kb)
Additional file 6: Nucleotide alignment for the *tef1* gene. (FASTA 40 kb)
Additional file 7: Best-fit evolutionary models predicted with Prottest 3.4 or jmodeltest-2.1.9 for each alignment. (PDF 12 kb)
Additional file 8: The partially conserved cluster found in *A. chrysogenum* and species from the *Arthroderma* genus is putatively linked to fusidic acid biosynthesis. (PDF 307 kb)
Additional file 9: Amino acid alignment for helvolic acid supermatrix. (FASTA 40 kb)
Additional file 10: Overview of the BGCs in *M. anisopliae* E6, their domain structures, and conservation among six *Metarhizium* species. (PDF 256 kb)
Additional file 11: Expression profile of predicted BGCs. (PDF 934 kb)
Additional file 12: Comparative genomic analysis and synteny for the aurovertin, elymoclavine/ergovaline-related compound, terpendole E/lolitrem-related compound, and xenolozoyenone-related compound BGCs. (PDF 161 kb)
Additional file 13: Additional phylogenetic trees. (PDF 463 kb)
