# Supplementary material for: Secondary metabolite gene clusters in the entomopathogen fungus Metarhizium anisopliae: genome identification and patterns of expression in a cuticle infection model
Source: BMC Genomics. 2016 Oct 25;17(Suppl 8):736. doi: 10.1186/s12864-016-3067-6 (PMC5088523; doi:10.1186/s12864-016-3067-6)
Supplement: Additional file 1: — Fungal genomes used in this work. (PDF 158 kb) [file 12864_2016_3067_MOESM1_ESM.pdf]

Additional File 1: Fungal genomes used in this work.

Genome	NCBI's Accession Number
**Genomes from *Metarhizium* species**	
***Metarhizium anisopliae* E6**	PRJNA245858
***Metarhizium robertsii* ARSEF23**	PRJNA38717
***Metarhizium acridum* CQMa102**	PRJNA38715
***Metarhizium majus* ARSEF297**	PRJNA184757
***Metarhizium guizhouense* ARSEF977**	PRJNA184755
***Metarhizium brunneum* ARSEF3297**	PRJNA184756
***Metarhizium album* ARSEF1941**	PRJNA72731
**Unannotated genomes**	
***Epichloë festucae* Fl1**	PRJNA51625
***Epichloë baconii* ATCC 200745**	PRJNA221976
***Balansia obtecta* B249**	PRJNA221345
***Epichloë sylvatica* GR10156**	PRJNA275112
***Neotyphodium gansuense* E7080**	PRJNA67299
***Pochonia chlamydosporia* 123**	PRJNA68669
***Periglandula ipomoeae* IasaF13**	PRJNA67303
***Aciculosporium take* MAFF-241224**	PRJNA67241
***Hypocrella siamensis* MTCC 10142**	PRJNA242986
***Atkinsonella hypoxylon* B4728**	PRJNA221544
***Claviceps fusiformis* PRL 1980**	PRJNA67243
**Genomes used for OrthoMCL clustering**	
***Metarhizium anisopliae* E6**	PRJNA245858
***Metarhizium robertsii* ARSEF23**	PRJNA38717
***Metarhizium acridum* CQMa102**	PRJNA38715
***Metarhizium majus* ARSEF297**	PRJNA184757
***Metarhizium guizhouense* ARSEF977**	PRJNA184755
***Metarhizium brunneum* ARSEF3297**	PRJNA184756
***Metarhizium album* ARSEF1941**	PRJNA72731
***Metarhizium anisopliae* E6**	PRJNA245858
***Acremonium chrysogenum* ATCC 11550**	PRJNA248608
***Aspergillus fumigatus* Af293**	PRJNA131
***Aspergillus nomius* NRRL 13137**	PRJNA246595
Coccidioides posadasii str. Silveira	PRJNA17787
***Leptosphaeria maculans* JN3**	PRJNA171003
***Penicillium digitatum* PHI26**	PRJNA157541
***Scedosporium apiospermum* IHEM 14462**	PRJNA244532
***Talaromyces marneffei* PM1**	PRJNA251717
***Talaromyces stipitatus* ATCC 10500**	PRJNA19557

***Thielavia terrestris* NRRL 8126**	PRJNA32847
***Tolyplocadium ophioglossoides* CBS 100239**	PRJNA91059
***Trichophyton soudanense* CBS 452.61**	PRJNA186831
***Arthroderma benhamiae* CBS 112371**	PRJNA30573
***Aspergillus clavatus* NRRL 1**	PRJNA15664
***Aspergillus fischeri* NRRL 181**	PRJNA15672
***Aspergillus niger* CBS 513.88**	PRJNA19275
***Arthroderma otae* CBS 113480**	PRJNA30939
Aspergillus udagawae	PRJDB3949
***Coccidioides immitis* RS**	PRJNA12883
***Coccidioides posadasii* RMSCC 3488**	PRJNA17783
Colletotrichum sublineola	PRJNA262370
***Microsporum gypseum* CBS 118893**	PRJNA20599
Madurella mycetomatis	PRJNA267680
***Neofusicoccum parvum* UCRNP2**	PRJNA187491
***Oidiodendron maius* Zn**	PRJNA74727
***Pseudogymnoascus sp.* VKM F-3557**	PRJNA216963
***Pyrenophora teres f. teres* 0-1**	PRJNA50389
Talaromyces cellulolyticus	PRJDB3250
Torrubiella hemipterigena	PRJEB7402
***Trichophyton interdigitale* H6**	PRJNA186827
Talaromyces islandicus	PRJEB8788
***Trichophyton rubrum* CBS 118892**	PRJNA38221
***Trichophyton verrucosum* HKI 0517**	PRJNA39693
