# Supplementary material for: Secondary metabolite gene clusters in the entomopathogen fungus Metarhizium anisopliae: genome identification and patterns of expression in a cuticle infection model
Source: BMC Genomics. 2016 Oct 25;17(Suppl 8):736. doi: 10.1186/s12864-016-3067-6 (PMC5088523; doi:10.1186/s12864-016-3067-6)
Supplement: Additional file 2: — OrthoMCL clustering results. (PDF 157 kb) [file 12864_2016_3067_MOESM2_ESM.pdf]

Additional File 2: OrthoMCL clustering results.

**MaPKS1 (MANI_014762) Backbone gene***	Acl|EAW12049.1 Acl|EAW12177.1 Afi|EAW17018.1<br>Afi|EAW19983.1 Afu|EAL84933.1 Afu|EAL86540.1<br>Ani|XP001390084.2 Ani|XP001393501.2<br>Ani|XP001395291.2 Ano|KNG83914.1 Ano|KNG86665.1<br>Ano|KNG91356.1 Aot|XP002843683.1 Aot|XP002846669.1<br>Aot|XP002848394.1 Aud|GAO81768.1 Aud|GAO86048.1<br>Aud|GAO89462.1 Aud|GAO90476.1 Cim|EAS31602.3<br>Cim|EAS32138.2 Cim|EAS33665.3 Cim|EAS34547.3<br>Cpo|EFW13280.1 Cpo|EFW17356.1 Cpo|EFW20973.1<br>Cpo|EFW23245.1 Cps|KMM68116.1 Cps|KMM70187.1<br>Cps|KMM71002.1 Cps|KMM72476.1 Csu|KDN60752.1<br>Csu|KDN67932.1 Csu|KDN68948.1 Csu|KDN72002.1<br>Mac|EFY84249.1 Mac|EFY85737.1 Mac|EFY89365.1<br>MANI_006324 MANI_020781 Mbr|KID60724.1<br>Mbr|KID72947.1 Mbr|KID74158.1 Mgu|KID81974.1<br>Mgu|KID82773.1 Mgu|KID83470.1 Mgu|KID84064.1<br>Mma|KID95125.1 Mma|KID96266.1 Mma|KID99274.1<br>Mmy|KXX79183.1 Mmy|KXX79217.1 Mro|EFY94576.2<br>Mro|EFY96642.1 Mro|EFY96950.1 Oma|KIM92947.1<br>Oma|KIM94086.1 Oma|KIM97742.1 Oma|KIN05207.1<br>Pdi|EKV06857.1 Pdi|EKV16250.1 Pte|EFQ90032.1<br>Pte|EFQ91438.1 Pte|EFQ92321.1 Tce|GAM33949.1<br>Tce|GAM33954.1 Tce|GAM33973.1 Tce|GAM35873.1<br>Tce|GAM41193.1 Tce|GAM41694.1 The|CEJ81468.1<br>The|CEJ90349.1 The|CEJ91591.1 Tis|CRG86486.1<br>Tis|CRG88297.1 Tis|CRG91771.1 Tis|CRG91775.1<br>Tis|CRG92018.1 Tma|KFX42009.1 Tma|KFX41537.1<br>Tma|KFX45541.1 Tma|KFX45542.1 Tma|KFX46688.1<br>Tma|KFX46693.1 Top|KND87339.1 Top|KND88239.1<br>Top|KND93702.1 Top|KND93710.1 Top|KND93709.1<br>Tst|EED12110.1 Tst|EED12124.1 Tst|EED18001.1<br>Tst|EED22580.1 Tst|EED22651.1 Tst|EED22678.1<br>Tst|EED22683.1 Tte|AEO64805.1 Tte|AEO67399.1<br>Tte|AEO68597.1 Tte|AEO67410.1 Abe|EFE29950.1<br>Ach|KFH44362.1 Ani|XP001393524.2 Lma|XP003842913.1<br>Mal|KHN98032.1 Mgy|EFR04739.1 Npa|EOD53036.1<br>Psp|KFX98702.1 Sap|KEZ43375.1 Tin|EZF36181.1<br>Tru|EGD89109.2 Tso|EZF74361.1 Tve|EFE42799.1<br>Ano|KNG83518.1 Lma|XP003842914.1 Psp|KFX99638.1
**MaNRPS-PKS2 (MANI_018878) Backbone gene***	Abe|EFE30182.1 Abe|EFE31625.1 Abe|EFE33181.1<br>Abe|EFE33214.1 Abe|EFE33589.1 Abe|EFE34630.1<br>Abe|EFE36394.1 Ach|KFH40504.1 Ach|KFH40639.1<br>Ach|KFH40913.1 Ach|KFH41335.1 Ach|KFH42117.1<br>Ach|KFH43187.1 Ach|KFH43887.1 Ach|KFH43961.1<br>Ach|KFH44143.1 Ach|KFH44396.1 Ach|KFH44962.1

	Ach|KFH45102.1 Ach|KFH45393.1 Ach|KFH45702.1<br>Ach|KFH46614.1 Ach|KFH47519.1 Ach|KFH44074.1<br>Ach|KFH42871.1 Ach|KFH45364.1 Ach|KFH40930.1<br>Ach|KFH44047.1 Ach|KFH44869.1 Acl|EAW07064.1<br>Acl|EAW07624.1 Acl|EAW08895.1 Acl|EAW09019.1<br>Acl|EAW09117.1 Acl|EAW11760.1 Acl|EAW12170.1<br>Acl|EAW12336.1 Acl|EAW12416.1 Acl|EAW13625.1<br>Acl|EAW14856.1 Acl|EAW08950.1 Acl|EAW10019.1<br>Acl|EAW13520.1 Acl|EAW13531.1 Acl|EAW15062.1<br>Afi|EAW16387.1 Afi|EAW16886.1 Afi|EAW17052.1<br>Afi|EAW17483.1 Afi|EAW19759.1 Afi|EAW21094.1<br>Afi|EAW23638.1 Afi|EAW23793.1 Afi|EAW16804.1<br>Afi|EAW20415.1 Afu|EAL85113.2 Afu|EAL86424.2<br>Afu|EAL89230.2 Afu|EAL91103.2 Afu|EAL85129.1<br>Afu|EAL87227.2 Afu|EAL92117.1 Afu|EAL87813.1<br>Ani|XP001388555.2 Ani|XP001389118.2 Ani|XP001390513.2<br>Ani|XP001391193.2 Ani|XP001393447.2<br>Ani|XP001393508.2 Ani|XP001394029.2<br>Ani|XP001394423.2 Ani|XP001394543.2 Ani|XP001395346.2<br>Ani|XP001395352.1 Ani|XP001397040.2 Ani|XP001398521.2<br>Ani|XP001400073.2 Ani|XP001389116.2 Ani|XP001390395.2<br>Ani|XP001394387.1 Ani|XP001394579.1 Ani|XP001396949.2<br>Ani|XP001392496.2 Ani|XP001394581.2 Ani|XP001396381.2<br>Ani|XP001396752.1 Ani|XP001397313.2 Ani|XP001401800.2<br>Ani|XP001402359.2 Ani|XP001393844.2 Ani|XP003188554.1<br>Ani|XP001394625.2 Ani|XP001396431.2 Ani|XP001399961.2<br>Ani|XP001400817.2 Ani|XP003188850.1 Ani|XP001395761.2<br>Ano|KNG81069.1 Ano|KNG81092.1 Ano|KNG81162.1<br>Ano|KNG82131.1 Ano|KNG83279.1 Ano|KNG83443.1<br>Ano|KNG83684.1 Ano|KNG84539.1 Ano|KNG85249.1<br>Ano|KNG87291.1 Ano|KNG88285.1 Ano|KNG88668.1<br>Ano|KNG88685.1 Ano|KNG90368.1 Ano|KNG90587.1<br>Ano|KNG90606.1 Ano|KNG90940.1 Ano|KNG91513.1<br>Ano|KNG89430.1 Ano|KNG91625.1 Ano|KNG84699.1<br>Ano|KNG85583.1 Ano|KNG90546.1 Ano|KNG91681.1<br>Aot|XP002842735.1<br>Aot|XP002843368.1 Aot|XP002843704.1 Aot|XP002844003.1<br>Aot|XP002846667.1 Aot|XP002846808.1 Aot|XP002847329.1<br>Aot|XP002847685.1 Aot|XP002850361.1 Aot|XP002842777.1<br>Aot|XP002843932.1 Aot|XP002847170.1 Aud|GAO81200.1<br>Aud|GAO81231.1 Aud|GAO81330.1 Aud|GAO81771.1<br>Aud|GAO81964.1 Aud|GAO82999.1 Aud|GAO83635.1<br>Aud|GAO85953.1 Aud|GAO85988.1 Aud|GAO86083.1<br>Aud|GAO86285.1 Aud|GAO87672.1 Aud|GAO87681.1<br>Aud|GAO88286.1 Aud|GAO90373.1 Aud|GAO90494.1<br>Aud|GAO90565.1 Aud|GAO81540.1 Aud|GAO86271.1<br>Cim|EAS31150.3 Cim|EAS34545.3 Cim|EAS31990.3

	Cim|EAS37044.3 Cpo|EFW14129.1 Cpo|EFW17358.1 Cpo|EFW16137.1 Cpo|EFW22349.1 Cps|KMM64104.1 Cps|KMM71000.1 Cps|KMM67662.1 Cps|KMM72316.1 Csu|KDN60538.1 Csu|KDN64005.1 Csu|KDN68160.1 Csu|KDN68596.1 Csu|KDN71930.1 Csu|KDN72402.1 Csu|KDN61564.1 Csu|KDN62563.1 Csu|KDN65093.1 Csu|KDN68946.1 Csu|KDN65693.1 Csu|KDN67074.1 Csu|KDN70595.1 Csu|KDN72279.1 Csu|KDN68224.1 Csu|KDN70032.1 Csu|KDN66389.1 Csu|KDN70949.1 Csu|KDN71389.1 Csu|KDN66474.1 Csu|KDN67432.1 Csu|KDN68757.1 Csu|KDN69972.1 Csu|KDN70187.1 Csu|KDN70914.1 Csu|KDN71859.1 Csu|KDN72352.1 Csu|KDN72099.1 Csu|KDN67744.1 Csu|KDN70961.1 Lma|XP003834096.1 Lma|XP003842471.1 Lma|XP003843013.1 Lma|XP003845353.1 Lma|XP003834103.1 Lma|XP003838273.1 Lma|XP003844080.1 Lma|XP003844958.1 Lma|XP003844774.1 Mac|EFY84462.1 Mac|EFY85749.1 Mac|EFY88029.1 Mac|EFY85592.1 Mac|EFY92873.1 Mal|KHN93792.1 Mal|KHN96044.1 Mal|KHO00577.1 Mal|KHN94261.1 Mal|KHN94311.1 Mal|KHN94379.1 MANI_001950 MANI_020791 MANI_004781 MANI_006158 MANI_006250 MANI_006783 MANI_110051 MANI_121659 MANI_023437 MANI_110978 MANI_012054 MANI_020125 MANI_021529 MANI_022470 MANI_025650 MANI_029455 Mbr|KID59724.1 Mbr|KID61511.1 Mbr|KID64201.1 Mbr|KID72760.1 Mbr|KID73874.1 Mbr|KID74205.1 Mbr|KID80235.1 Mbr|KID59833.1 Mbr|KID61230.1 Mbr|KID66135.1 Mbr|KID67785.1 Mbr|KID62105.1 Mbr|KID65387.1 Mbr|KID74161.1 Mbr|KID79685.1 Mbr|KID62944.1 Mbr|KID74173.1 Mbr|KID75387.1 Mbr|KID71992.1 Mgu|KID81493.1 Mgu|KID84050.1 Mgu|KID89863.1 Mgu|KID81569.1 Mgu|KID82712.1 Mgu|KID84197.1 Mgu|KID84859.1 Mgu|KID81791.1 Mgu|KID82218.1 Mgu|KID82736.1 Mgu|KID83952.1 Mgu|KID84093.1 Mgu|KID86635.1 Mgu|KID91268.1 Mgu|KID84061.1 Mgu|KID87248.1 Mgu|KID91408.1 Mgu|KID86000.1 Mgu|KID86016.1 Mgy|EFQ96791.1 Mgy|EFQ96831.1 Mgy|EFQ96902.1 Mgy|EFQ96996.1 Mgy|EFQ97313.1 Mgy|EFQ98465.1 Mgy|EFR01005.1 Mgy|EFR01671.1 Mgy|EFQ99795.1 Mgy|EFR00806.1 Mgy|EFR04190.1 Mgy|EFR04320.1 Mgy|EFR04828.1 Mma|KID98764.1 Mma|KID97497.1 Mma|KID90834.1 Mma|KID94120.1 Mma|KID94121.1 Mma|KID91313.1 Mma|KID96263.1 Mma|KID96316.1 Mma|KID97531.1 Mma|KID98721.1 Mma|KID99909.1

	Mma|KID93355.1	Mma|KID93652.1	Mma|KID94822.1
	Mma|KID93461.1	Mma|KIE01334.1	Mmy|KXX72797.1
	Mmy|KXX74576.1	Mmy|KXX79732.1	Mmy|KXX75271.1
	Mmy|KXX75424.1	Mmy|KXX75968.1	Mmy|KXX76480.1
	Mmy|KXX76491.1	Mmy|KXX77494.1	Mmy|KXX79710.1
	Mmy|KXX82443.1	Mmy|KXX74770.1	Mmy|KXX77535.1
	Mmy|KXX82904.1	Mmy|KXX75016.1	Mmy|KXX76983.1
	Mmy|KXX79394.1	Mmy|KXX80750.1	Mmy|KXX81008.1
	Mmy|KXX82176.1	Mmy|KXX75870.1	Mmy|KXX83260.1
	Mmy|KXX82669.1	Mmy|KXX80977.1	Mro|EFY94315.2
	Mro|EFY94437.2	Mro|EFY94573.1	Mro|EFY95891.1
	Mro|EFY96026.1	Mro|EFY96062.1	Mro|EFY96790.1
	Mro|EFY98483.1	Mro|EFZ00678.1	Mro|EFZ03715.1
	Mro|EFY94872.2	Mro|EFY96172.2	Mro|EFY96241.2
	Mro|EFZ02044.1	Mro|EFZ03354.1	Mro|EFZ04272.1
	Mro|EFY94490.2	Mro|EFY97775.2	Mro|EFZ02119.2
	Mro|KHO10995.1	Mro|KHO10996.1	Npa|EOD43154.1
	Npa|EOD51988.1	Npa|EOD43327.1	Npa|EOD46651.1
	Npa|EOD50138.1	Npa|EOD43341.1	Npa|EOD43393.1
	Npa|EOD43397.1	Npa|EOD45881.1	Npa|EOD49628.1
	Npa|EOD51113.1	Npa|EOD53030.1	Npa|EOD48494.1
	Npa|EOD47371.1	Npa|EOD51041.1	Oma|KIM92741.1
	Oma|KIM92842.1	Oma|KIM93266.1	Oma|KIM93865.1
	Oma|KIM94019.1	Oma|KIM94092.1	Oma|KIM94705.1
	Oma|KIM94801.1	Oma|KIM95579.1	Oma|KIM95626.1
	Oma|KIM97074.1	Oma|KIM97314.1	Oma|KIM98589.1
	Oma|KIM99715.1	Oma|KIM99782.1	Oma|KIN00612.1
	Oma|KIN05352.1	Oma|KIN05356.1	Oma|KIN05364.1
	Oma|KIN06913.1	Oma|KIN09038.1	Oma|KIM92883.1
	Oma|KIM94486.1	Oma|KIM97138.1	Oma|KIM97381.1
	Oma|KIM99830.1	Oma|KIN02399.1	Pdi|EKV04424.1
	Pdi|EKV05514.1	Pdi|EKV06858.1	Pdi|EKV11465.1
	Pdi|EKV12048.1	Pdi|EKV13466.1	Pdi|EKV17385.1
	Pdi|EKV10561.1	Pdi|EKV19716.1	Pdi|EKV06097.1
	Pdi|EKV13495.1	Pdi|EKV13692.1	Pdi|EKV18177.1
	Psp|KFX86927.1	Psp|KFX96803.1	Psp|KFX98712.1
	Psp|KFY03516.1	Psp|KFX98753.1	Psp|KFY01188.1
	Pte|EFQ85105.1	Pte|EFQ85729.1	Pte|EFQ86427.1
	Pte|EFQ87243.1	Pte|EFQ88351.1	Pte|EFQ91289.1
	Pte|EFQ92322.1	Pte|EFQ92956.1	Pte|EFQ93721.1
	Pte|EFQ94415.1	Pte|EFQ85730.1	Pte|EFQ90137.1
	Pte|EFQ90138.1	Pte|EFQ87497.1	Pte|EFQ88531.1
	Pte|EFQ89733.1	Pte|EFQ92739.1	Pte|EFQ95051.1
	Sap|KEZ39405.1	Sap|KEZ40049.1	Sap|KEZ40612.1
	Sap|KEZ41293.1	Sap|KEZ45498.1	Sap|KEZ45627.1
	Sap|KEZ46536.1	Sap|KEZ40559.1	Sap|KEZ40819.1
	Sap|KEZ42792.1	Sap|KEZ43366.1	Tce|GAM33530.1

	Tce|GAM35872.1	Tce|GAM36828.1	Tce|GAM37528.1
	Tce|GAM37666.1	Tce|GAM41922.1	Tce|GAM42808.1
	Tce|GAM44116.1	Tce|GAM35366.1	Tce|GAM41187.1
	Tce|GAM42162.1	Tce|GAM42968.1	Tce|GAM38546.1
	Tce|GAM41899.1	Tce|GAM41924.1	Tce|GAM42258.1
	Tce|GAM42393.1	Tce|GAM40954.1	The|CEJ80659.1
	The|CEJ86142.1	The|CEJ86637.1	The|CEJ86639.1
	The|CEJ91944.1	The|CEJ92557.1	The|CEJ81082.1
	The|CEJ90334.1	The|CEJ94083.1	The|CEJ81590.1
	The|CEJ86573.1	The|CEJ82961.1	The|CEJ82962.1
	The|CEJ91945.1	Tin|EZF33319.1	Tin|EZF33403.1
	Tin|EZF33994.1	Tin|EZF35978.1	Tin|EZF36261.1
	Tis|CRG82770.1	Tis|CRG86478.1	Tis|CRG86497.1
	Tis|CRG86530.1	Tis|CRG87308.1	Tis|CRG87892.1
	Tis|CRG89167.1	Tis|CRG89192.1	Tis|CRG90947.1
	Tis|CRG90972.1	Tis|CRG92461.1	Tis|CRG92673.1
	Tis|CRG92715.1	Tis|CRG83422.1	Tis|CRG83700.1
	Tis|CRG83761.1	Tis|CRG86029.1	Tis|CRG87956.1
	Tis|CRG90802.1	Tis|CRG90967.1	Tis|CRG91166.1
	Tis|CRG91965.1	Tis|CRG92280.1	Tis|CRG92723.1
	Tis|CRG85198.1	Tis|CRG90265.1	Tma|KFX41168.1
	Tma|KFX41169.1	Tma|KFX44029.1	Tma|KFX41560.1
	Tma|KFX41251.1	Tma|KFX41252.1	Tma|KFX41383.1
	Tma|KFX46785.1	Tma|KFX51797.1	Tma|KFX52865.1
	Tma|KFX43763.1	Tma|KFX43764.1	Tma|KFX44733.1
	Tma|KFX48303.1	Tma|KFX52867.1	Top|KND86883.1
	Top|KND86893.1	Top|KND87119.1	Top|KND87310.1
	Top|KND87695.1	Top|KND88257.1	Top|KND89917.1
	Top|KND92549.1	Top|KND94632.1	Top|KND87975.1
	Tru|EGD84902.1	Tru|EGD85602.1	Tru|EGD86527.2
	Tru|EGD86562.1	Tru|EGD91141.1	Tso|EZF68845.1
	Tso|EZF71267.1	Tso|EZF72769.1	Tso|EZF72812.1
	Tso|EZF77964.1	Tst|EED11515.1	Tst|EED11953.1
	Tst|EED12350.1	Tst|EED13058.1	Tst|EED13571.1
	Tst|EED13647.1	Tst|EED14251.1	Tst|EED14366.1
	Tst|EED14393.1	Tst|EED14463.1	Tst|EED15402.1
	Tst|EED15547.1	Tst|EED15709.1	Tst|EED15802.1
	Tst|EED16637.1	Tst|EED18128.1	Tst|EED18841.1
	Tst|EED21572.1	Tst|EED24614.1	Tst|EED16635.1
	Tst|EED19830.1	Tst|EED21266.1	Tte|AEO62906.1
	Tte|AEO67408.1	Tte|AEO63908.1	Tte|AEO67303.1
	Tte|AEO68365.1	Tte|AEO64476.1	Tte|AEO67427.1
	Tte|AEO68274.1	Tve|EFE38442.1	Tve|EFE41038.1
	Tve|EFE44178.1	Tve|EFE40994.1	Tve|EFE41543.1
	Tve|EFE44717.1	Tve|EFE41155.1	Tin|EZF33402.1
	Cim|KJF61407.1	Cpo|EFW19798.1	Cps|KMM72254.1
	Cps|KMM72255.1	Mma|KID90835.1	Tru|KFL60619.1

	Tso|EZF68429.1 Tso|EZF68430.1 Mgy|EFQ96956.1 Mgy|EFQ96957.1 Mgy|EFQ96958.1 Sap|KEZ40857.1 Tin|EZF34624.1 Tru|EGD86700.2 Tso|EZF72989.1 The|CEJ93554.1 Mac|EFY92579.1 Oma|KIM92884.1 Psp|KFY00590.1 Mac|EFY84245.1 Mma|KID96522.1 Mbr|KID61232.1 Tce|GAM41182.1 Mma|KID96523.1 Top|KND87976.1 MANI_001951 Aud|GAO90371.1 Aot|XP002847311.1 Afu|EAL86536.1 Ani|XP003188799.1 Ani|XP003188800.1 Afi|EAW19771.1 Csu|KDN72130.1 Afi|EAW24573.1 Afu|EAL86636.1 Ani|XP003188601.1 Ani|XP003188851.1 Ano|KNG83926.1 Aot|XP002845180.1 Npa|EOD45514.1 Mac|EFY88214.1 Lma|XP003841875.1 Npa|EOD51963.1 Mac|EFY88213.1 Pdi|EKV13493.1 Mac|EFY91835.1 Mma|KID95486.1 Tst|EED13941.1 MANI_0115518 Mro|KHO10625.1 Mbr|KID73295.1 Mbr|KID75468.1 Mro|EFY94354.1 Pdi|EKV14022.1 Pdi|EKV18176.1 Tce|GAM36740.1
**MaTERP1 (MANI_010532) Backbone gene***	Ach|KFH43011.1 Afi|EAW24654.1 Afu|EAL89316.1 Aot|XP002843072.1 Aud|GAO81617.1 Mbr|KID65418.1 Mgu|KID82793.1 Mgy|EFQ96702.1 Mma|KID93904.1 Mro|EFY97806.1
**MaTERP1 (MANI_010531) Backbone gene***	Ach|KFH43021.1 Afi|EAW24653.1 Afu|EAL89317.1 Aot|XP002843067.1 Aud|GAO81618.1 Mbr|KID65414.1 Mgu|KID82789.1 Mgy|EFQ96706.1 Mma|KID93897.1 Mro|EFY97802.2
MaTERP1* (MANI_010536)	Ach|KFH40562.1 Afi|EAW24657.1 Afu|EAL89313.1 Aud|GAO81614.1 Mbr|KID65421.1 Mgu|KID82796.1 Mma|KID93907.1 Mro|EFY97809.1
**MaTERP1 (MANI_010527) Backbone gene***	Ach|KFH40555.1 Afi|EAW24656.1 Afu|EAL89314.2 Aud|GAO81615.1 Mbr|KID65422.1 Mgu|KID82797.1 Mma|KID93908.1 Mro|EFY97810.1
**MaTERP1 (MANI_010537)***	Ach|KFH43032.1 Afi|EAW24659.1 Afu|EAL89311.1 Aot|XP002843073.1 Aud|GAO81612.1 Mbr|KID65419.1 Mgu|KID82794.1 Mgy|EFQ96701.1 Mma|KID93905.1 Mro|EFY97807.1
**MaTERP1 (MANI_010530) Backbone gene***	Ach|KFH43043.1 Afi|EAW24658.1 Afu|EAL89312.1 Aot|XP002843068.1 Aud|GAO81613.1 Mbr|KID65415.1 Mgu|KID82790.1 Mgy|EFQ96705.1 Mma|KID93898.1 Mro|EFY97803.1
**MaTERP1 (MANI_010495)***	Ach|KFH43040.1 Afi|EAW24652.1 Afu|EAL89318.1 Aud|GAO81619.1 Mbr|KID65416.1 Mgu|KID82791.1 Mgy|EFQ96704.1 Mma|KID93899.1 Mro|EFY97804.1
**MaTERP1 (MANI_010512)***	MANI_025019 Mbr|KID63604.1 Mbr|KID65420.1 Mgu|KID82795.1 Mgu|KID85770.1 Mro|EFY95587.1 Mro|EFY97808.1 Abe|EFE36095.1 Ach|KFH43984.1 Afi|EAW24660.1 Afu|EAL89310.1 Aot|XP002846965.1

	Aud|GAO81611.1 Mac|EFY86899.1 Mal|KHN94024.1 Mgy|EFQ99030.1 Mma|KID93906.1 Npa|EOD52809.1 Oma|KIM93336.1 The|CEJ93288.1 Tin|EZF34275.1 Top|KND92088.1 Tso|EZF72233.1 Tve|EFE43788.1 Aud|GAO90117.1 Oma|KIM93571.1 Mma|KID80879.1 Top|KND92083.
**MaTERP1 (MANI_010594)***	Ach|KFH43025.1 Afi|EAW24655.1 Afu|EAL89315.1 Aot|XP002843071.1 Aud|GAO81616.1 Mbr|KID65417.1 Mgu|KID82792.1 Mgy|EFQ96703.1 Mma|KID93903.1 Mro|EFY97805.1
**Transcription factor embedded in MaPKS1 cluster (MANI_112402)**	Tma|KFX45543.1 Tma|KFX45544.1 Tma|KFX45545.1 Tma|KFX45546.1 Tma|KFX45547.1 Ani|XP001395290.1 Ach|KFH44401.1 Acl|EAW15012.1 Ano|KNG91357.1 Cim|EAS31607.3 Cpo|EFW23250.1 Cps|KMM68122.1 Lma|XP003843017.1 Mal|KHN98029.1 Mma|KID99277.1 Mmy|KXX77532.1 Npa|EOD53046.1 Oma|KIM94093.1 Psp|KFY01609.1 Tce|GAM33907.1 Tis|CRG91749.1 Top|KND87343.1 Tst|EED13248.1 Tte|AEO67302.1 Ani|XP001395307.2 Mac|EFY89368.1 Mbr|KID72944.1 Mgu|KID83467.1 Mro|EFY96639.1 Pdi|EKV19054.1 Pte|EFQ92323.1 The|CEJ92460.1 Tma|KFX46639.1 Top|KND88237.1 Ani|XP001393502.1 Ano|KNG85002.1 Aud|GAO81772.1 The|CEJ91586.1 Tst|EED12116.1 Tce|GAM33972.1 Tst|EED22652.1
**Transcription factor embedded in MaNRPS-PKS2 cluster (MANI_018928)**	Afi|EAW19778.1 Afu|EAL85124.1 Aud|GAO86281.1 Ano|KNG84526.1 Mbr|KID73871.1 Mro|EFY96238.2 Top|KND88287.1 Tte|AEO64233.1

Table 1: Abe| *Arthroderma benhamiae* CBS 112371, Ach| *Acremonium chrysogenum* ATCC 11550, Acl| *Aspergillus clavatus* NRRL 1, Afi| *Aspergillus fischeri* NRRL 181, Afu| *Aspergillus fumigatus* Af293, Ani| *Aspergillus niger* CBS 513.88, Ano| *Aspergillus nomius* NRRL 13137, Aot| *Arthroderma otae* CBS 113480, Aud| *Aspergillus udagawae*, Cim| *Coccidioides immitis* RS, Cpo| *Coccidioides posadasii str. Silveira*, Cps| *Coccidioides posadasii* RMSCC 3488, Csu| *Colletotrichum sublineola*, Lma| *Leptosphaeria maculans* JN3, Mac| *Metarhizium acridum* CQMa 102, Mal| *Metarhizium album* ARSEF 1941, MANI *Metarhizium anisopliae* E6, Mbr| *Metarhizium brunneum* ARSEF 3297, Mgu| *Metarhizium guizhouense* ARSEF 977, Mgy| *Microsporum gypseum* CBS 118893, Mma| *Metarhizium majus* ARSEF 297, Mmy| *Madurella mycetomatis*, Mro| *Metarhizium robertsii* ARSEF 23, Npa| *Neofusicoccum parvum* UCRNP2, Oma| *Oidiodendron maius* Zn, Pdi| *Penicillium digitatum* PHI26, Psp| *Pseudogymnoascus sp*. VKM F-3557, Pte| Pyrenophora teres f. teres 0-1, Sap| *Scedosporium*

apiospermum, Tce| *Talaromyces cellulolyticus*, The| *Torrubiella hemipterigena*, Tin| *Trichophyton interdigitale* H6, Tis| *Talaromyces islandicus*, Tma| *Talaromyces marneffei* PM1, Top| *Tolypocladium ophioglossoides* CBS 100239, Tru| *Trichophyton rubrum* CBS 118892, Tso| *Trichophyton soudanense* CBS 452.61, Tst| *Talaromyces stipitatus* ATCC 10500, Tte| *Thielavia terrestris* NRRL 8126, Tve| *Trichophyton verrucosum* HKI 0517. *Only genes with ≥ 45 % identity were used for phylogeny;
