# Supplementary material for: Secondary metabolite gene clusters in the entomopathogen fungus Metarhizium anisopliae: genome identification and patterns of expression in a cuticle infection model
Source: BMC Genomics. 2016 Oct 25;17(Suppl 8):736. doi: 10.1186/s12864-016-3067-6 (PMC5088523; doi:10.1186/s12864-016-3067-6)
Supplement: Additional file 3: — Phylogenetic trees constructed with putative orthologs of MaPKS1, all PKS from M. anisopliae E6, and all characterized PKS from MIBiG. (PDF 600 kb) [file 12864_2016_3067_MOESM3_ESM.pdf]

Additional File 3: Phylogenetic trees constructed with putative orthologs of MaPKS1, all PKS from *M. anisopliae* E6, and all characterized PKS from MIBiG**.**

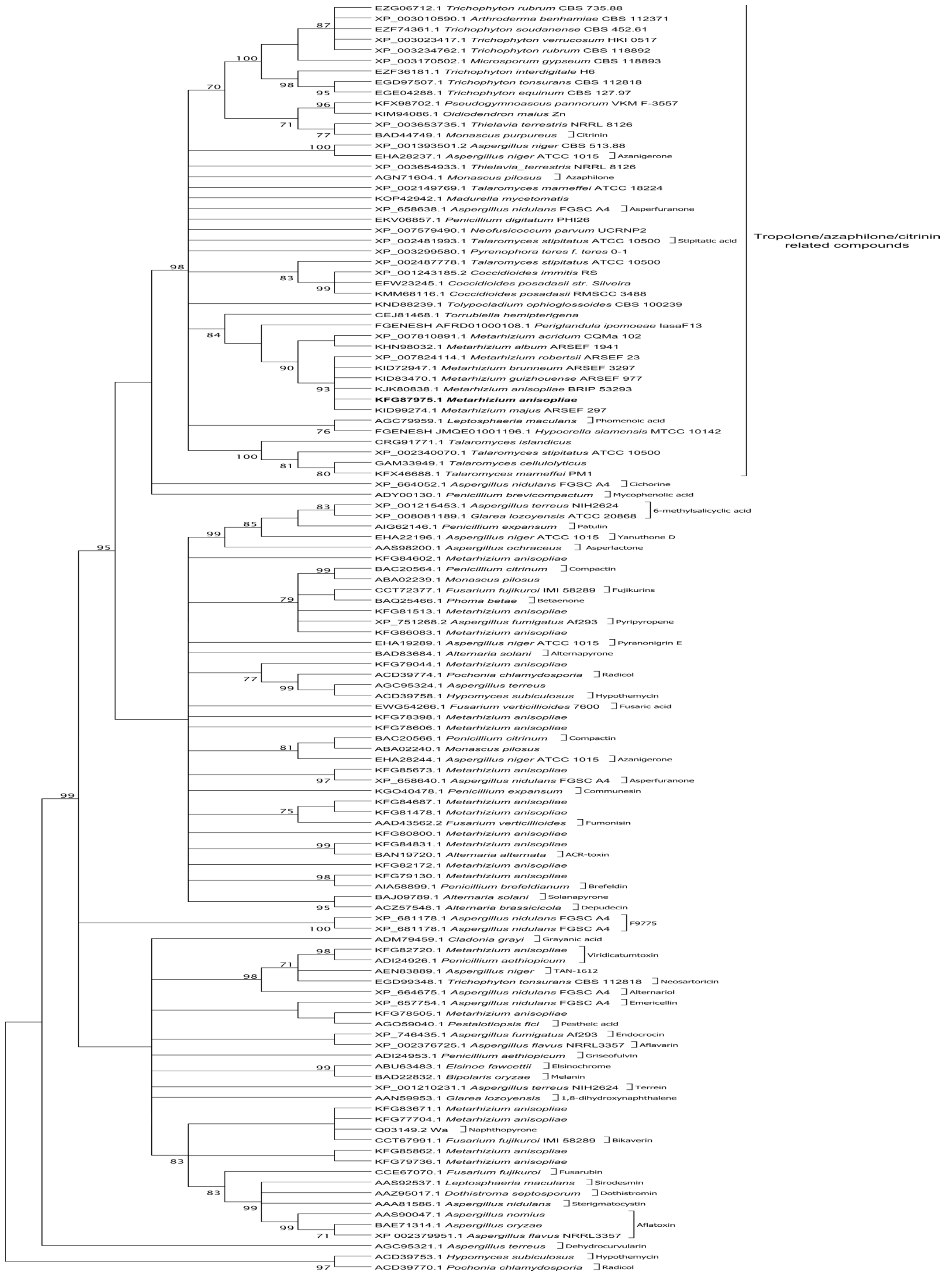

Figure 1: Maximum Parsimony analysis of taxa. The evolutionary history was inferred using the Maximum Parsimony method. The most parsimonious tree with length = 3580 is shown. The consistency index is (0.311236), the retention index is (0.658923), and the composite index is 0.207616 (0.205081) for all sites and parsimony-informative sites. The percentage of replicate trees in which the associated taxa clustered together in the bootstrap test (1000 replicates) are shown next to the branches. The Maximum Parsimony tree was obtained using the Subtree-Pruning-Regrafting (SPR) algorithm with search level 1 in which the initial trees were obtained by the random addition of sequences (10 replicates). The analysis involved 120 amino acid sequences. All positions containing gaps and missing data were eliminated. There were a total of 168 positions in the final dataset. Evolutionary analyses were conducted in MEGA6.

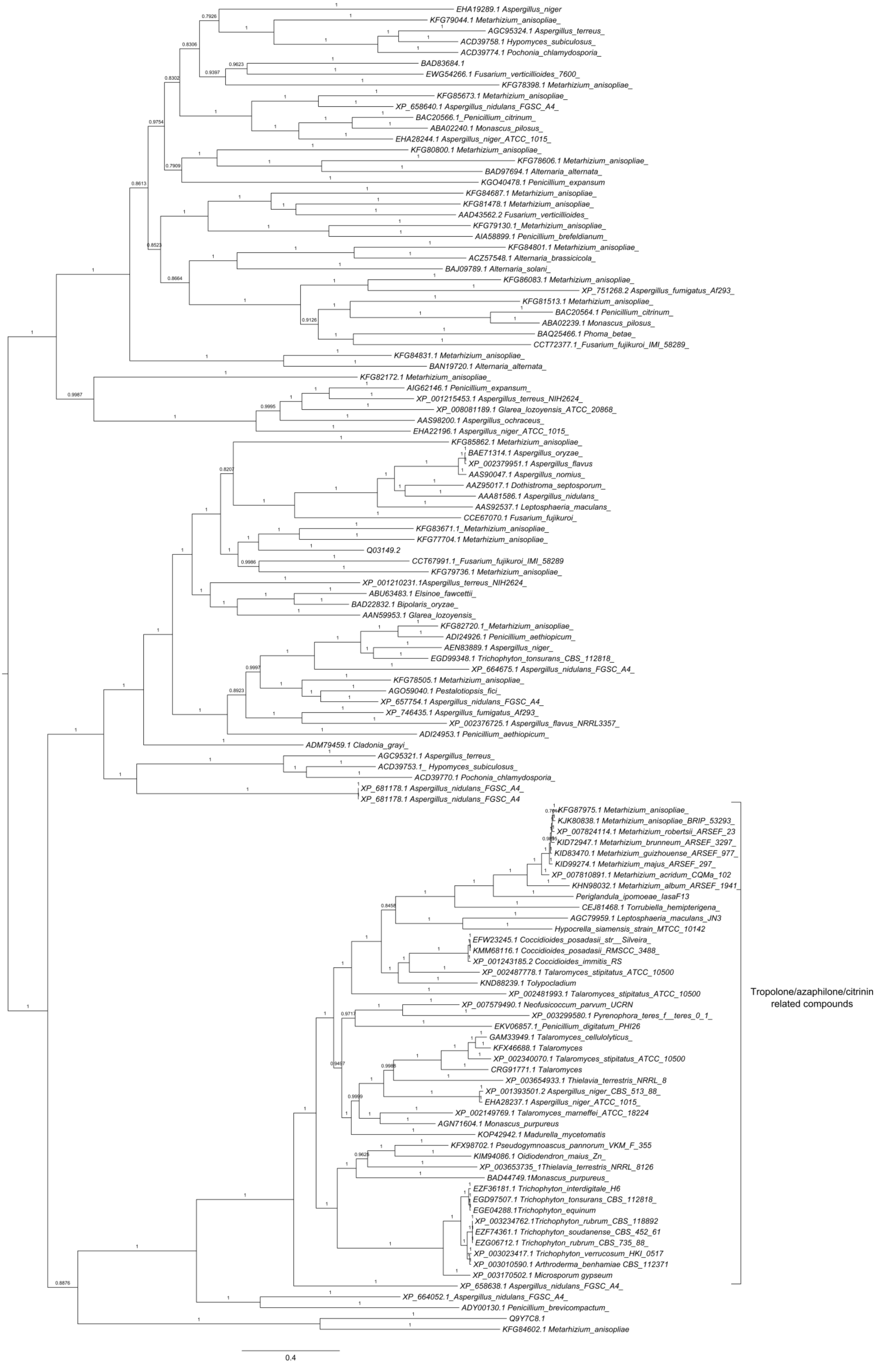
EHA19289.1 Aspergillus_niger
KFG79044.1 Metarhizium_anisopliae_
AGC95324.1 Aspergillus_terreus_
ACD39758.1 Hypomyces_subiculosus_
ACD39774.1 Pochonia_chlamydosporia_
BAD83684.1
EWG54266.1 Fusarium_verticillioides_7600_
KFG78398.1 Metarhizium_anisopliae_
KFG85673.1 Metarhizium_anisopliae_
XP_658640.1 Aspergillus_nidulans_FGSC_A4_
BAC20566.1_Penicillium_citrinum_
ABA02240.1 Monascus_pilosus_
EHA28244.1 Aspergillus_niger_ATCC_1015_
KFG80800.1 Metarhizium_anisopliae_
KFG78606.1 Metarhizium_anisopliae_
BAD97694.1 Alternaria_alternata_
KGO40478.1 Penicillium_expansum
KFG84687.1 Metarhizium_anisopliae_
KFG81478.1 Metarhizium_anisopliae_
AAD43562.2 Fusarium_verticillioides_
KFG79130.1_Metarhizium_anisopliae_
AIA58899.1 Penicillium_brefeldianum_
KFG84801.1 Metarhizium_anisopliae_
ACZ57548.1 Alternaria_brassicicola_
BAJ09789.1 Alternaria_solani_
KFG86083.1 Metarhizium_anisopliae_
XP_751268.2 Aspergillus_fumigatus_Af293_
KFG81513.1 Metarhizium_anisopliae_
BAC20564.1 Penicillium_citrinum_
ABA02239.1 Monascus_pilosus_
BAQ25466.1 Phoma_betae_
CCT72377.1_Fusarium_fujikuroi_IMI_58289_
KFG84831.1 Metarhizium_anisopliae_
BAN19720.1 Alternaria_alternata_
KFG82172.1 Metarhizium_anisopliae_
AIG62146.1 Penicillium_expansum_
XP_001215453.1 Aspergillus_terreus_NIH2624_
XP_008081189.1 Glarea_lozoyensis_ATCC_20868_
AAS98200.1 Aspergillus_ochraceus_
EHA22196.1 Aspergillus_niger_ATCC_1015_
KFG85862.1 Metarhizium_anisopliae_
BAE71314.1 Aspergillus_oryzae_
XP_002379951.1 Aspergillus_flavus
AAS90047.1 Aspergillus_nomius_
AAZ95017.1 Dothistroma_septosporum_
AAA81586.1 Aspergillus_nidulans_
AAS92537.1 Leptosphaeria_maculans_
CCE67070.1 Fusarium_fujikuroi_
KFG83671.1_Metarhizium_anisopliae_
KFG77704.1 Metarhizium_anisopliae_
Q03149.2
CCT67991.1_Fusarium_fujikuroi_IMI_58289
KFG79736.1 Metarhizium_anisopliae_
XP_001210231.1Aspergillus_terreus_NIH2624_
ABU63483.1 Elsinoe_fawcettii_
BAD22832.1 Bipolaris_oryzae_
AAN59953.1 Glarea_lozoyensis_
KFG82720.1_Metarhizium_anisopliae_
ADI24926.1 Penicillium_aethiopicum_
AEN83889.1 Aspergillus_niger_
EGD99348.1 Trichophyton_tonsurans_CBS_112818_
XP_664675.1 Aspergillus_nidulans_FGSC_A4_
KFG78505.1 Metarhizium_anisopliae_
AGO59040.1 Pestalotiopsis_fici_
XP_657754.1 Aspergillus_nidulans_FGSC_A4_
XP_746435.1 Aspergillus_fumigatus_Af293_
XP_002376725.1 Aspergillus_flavus_NRRL3357_
ADI24953.1 Penicillium_aethiopicum_
ADM79459.1 Cladonia_grayi_
AGC95321.1 Aspergillus_terreus_
ACD39753.1_ Hypomyces_subiculosus_
ACD39770.1 Pochonia_chlamydosporia_
XP_681178.1 Aspergillus_nidulans_FGSC_A4_
XP_681178.1 Aspergillus_nidulans_FGSC_A4
KFG87975.1 Metarhizium_anisopliae_
KJK80838.1 Metarhizium_anisopliae_BRIP_53293_
XP_007824114.1 Metarhizium_robertsii_ARSEF_23
KID72947.1 Metarhizium_brunneum_ARSEF_3297_
KID83470.1 Metarhizium_guizhouense_ARSEF_977_
KID99274.1 Metarhizium_majus_ARSEF_297_
XP_007810891.1 Metarhizium_acridum_CQMa_102
KHN98032.1 Metarhizium_album_ARSEF_1941_
Periglandula_ipomoeae_IasaF13
CEJ81468.1 Torrubiella_hemipterigena_
AGC79959.1 Leptosphaeria_maculans_JN3
Hypocrella_siamensis_strain_MTCC_10142
EFW23245.1 Coccidioides_posadasii_str__Silveira_
KMM68116.1 Coccidioides_posadasii_RMSCC_3488_
XP_001243185.2 Coccidioides_immitis_RS
XP_002487778.1 Talaromyces_stipitatus_ATCC_10500
KND88239.1 Tolypocladium
XP_002481993.1 Talaromyces_stipitatus_ATCC_10500
XP_007579490.1 Neofusicoccum_parvum_UCRN
XP_003299580.1 Pyrenophora_teres_f__teres_0_1_
EKV06857.1_Penicillium_digitatum_PHI26
GAM33949.1 Talaromyces_cellulolyticus_
KFX46688.1 Talaromyces
XP_002340070.1 Talaromyces_stipitatus_ATCC_10500
CRG91771.1 Talaromyces
XP_003654933.1 Thielavia_terrestris_NRRL_8
XP_001393501.2 Aspergillus_niger_CBS_513_88_
EHA28237.1 Aspergillus_niger_ATCC_1015_
XP_002149769.1 Talaromyces_marneffei_ATCC_18224
AGN71604.1 Monascus_purpureus
KOP42942.1 Madurella_mycetomatis
KFX98702.1 Pseudogymnoascus_pannorum_VKM_F_355
KIM94086.1 Oidiodendron_maius_Zn_
XP_003653735_1Thielavia_terrestris_NRRL_8126
BAD44749.1Monascus_purpureus_
EZF36181.1 Trichophyton_interdigitale_H6
EGD97507.1 Trichophyton_tonsurans_CBS_112818_
EGE04288.1Trichophyton_equinum
XP_003234762.1Trichophyton_rubrum_CBS_118892
EZF74361.1 Trichophyton_soudanense_CBS_452_61
EZG06712.1 Trichophyton_rubrum_CBS_735_88_
XP_003023417.1 Trichophyton_verrucosum_HKI_0517
XP_003010590.1 Arthroderma_benhamiae CBS_112371
XP_003170502.1 Microsporum_gypseum
XP_658638.1 Aspergillus_nidulans_FGSC_A4_
XP_664052.1_Aspergillus_nidulans_FGSC_A4_
ADY00130.1 Penicillium_brevicompactum_
Q9Y7C8.1
KFG84602.1 Metarhizium_anisopliae
Tropolone/azaphilone/citrinin
related compounds
0.4

Figure 2: Bayesian analysis of taxa. Branches support values (Bayesian posterior probability) are associated with nodes. The Bayesian inference ran for 3,125,600 generations.
