# Supplementary material for: Secondary metabolite gene clusters in the entomopathogen fungus Metarhizium anisopliae: genome identification and patterns of expression in a cuticle infection model
Source: BMC Genomics. 2016 Oct 25;17(Suppl 8):736. doi: 10.1186/s12864-016-3067-6 (PMC5088523; doi:10.1186/s12864-016-3067-6)
Supplement: Additional file 7: — Best-fit evolutionary models predicted with Prottest 3.4 or jmodeltest-2.1.9 for each alignment. (PDF 12 kb) [file 12864_2016_3067_MOESM7_ESM.pdf]

Additional File 7: Best-fit evolutionary models predicted with Prottest 3.4 or jmodeltest-2.1.9 for each alignment.

Locus tag/Gene/Tree	Model
MaNRPS-PKS2 (MANI_018878)	JTT+I+G+F
MaPKS1 (MANI_014762)	LG+I+G+F
MaTERP1 (MANI_010532)	LG+G
MaTERP1 (MANI_010531)	LG+I+G+F
MaTERP1 (MANI_010536)	LG+I+G+F
MaTERP1 (MANI_010537)	JTT+G+F
MaTERP1 (MANI_010495)	LG+I+G+F
MaTERP1 (MANI_010530)	LG+I+G+F
MaTERP1 (MANI_010594)	LG+G+F
MaTERP1 (MANI_010512)	LG+G+F
MaTERP1 (MANI_010527)	LG+G+F
tef1	GTR+I+G
supermatrix	JTT+G
All PKS tree	LG+I+G+F
