# Supplementary material for: Secondary metabolite gene clusters in the entomopathogen fungus Metarhizium anisopliae: genome identification and patterns of expression in a cuticle infection model
Source: BMC Genomics. 2016 Oct 25;17(Suppl 8):736. doi: 10.1186/s12864-016-3067-6 (PMC5088523; doi:10.1186/s12864-016-3067-6)
Supplement: Additional file 8: — The partially conserved cluster found in A. chrysogenum and species from the Arthroderma genus is putatively linked to fusidic acid biosynthesis. (PDF 307 kb) [file 12864_2016_3067_MOESM8_ESM.pdf]

Additional File 8: The partially conserved cluster found in *A. chrysogenum* and species from the *Arthroderma* genus is putatively linked to fusidic acid biosynthesis.

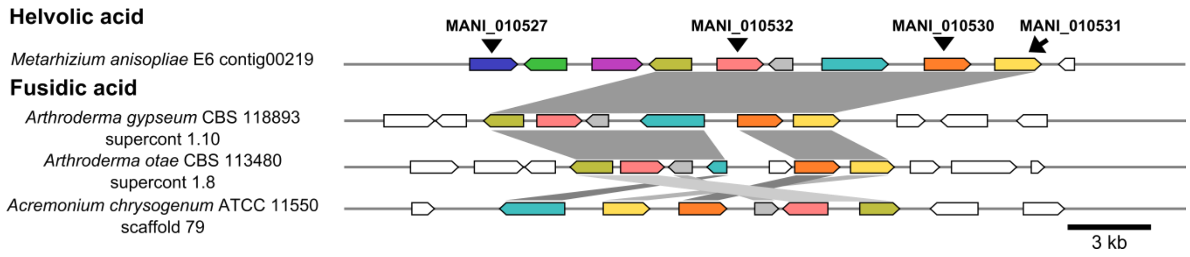

Figure 1: The search for orthologous of MaTERP1 (helvolic acid cluster) has found that this cluster is partially conserved in species from the *Arthroderma* genus and *Acremonium chrysogenum* (Hypocreales: Hypocreaceae). However, this partially conserved cluster was suggested to produce fusidic acid, a molecule similar to helvolic acid, this is supported by isolation of fusidic acid-like compounds from *Acremonium* spp. [1, 2].

1. Evans L, Hedger JN, Brayford D, Stavri M, Smith E, O'Donnell G, Gray AI, Griffith GW, Gibbons S: **An antibacterial hydroxy fusidic acid analogue from Acremonium crotocinigenum**. *Phytochemistry* 2006, **67**(19):2110-2114.
2. Lodeiro S, Xiong QB, Wilson WK, Ivanova Y, Smith ML, May GS, Matsuda SPT: **Protostadienol Biosynthesis and Metabolism in the Pathogenic Fungus Aspergillus fumigatus**. *Organic Letters* 2009, **11**(6):1241-1244.
