# Supplementary material for: Secondary metabolite gene clusters in the entomopathogen fungus Metarhizium anisopliae: genome identification and patterns of expression in a cuticle infection model
Source: BMC Genomics. 2016 Oct 25;17(Suppl 8):736. doi: 10.1186/s12864-016-3067-6 (PMC5088523; doi:10.1186/s12864-016-3067-6)
Supplement: Additional file 10: — Overview of the BGCs in M. anisopliae E6, their domain structures, and conservation among six Metarhizium species. (PDF 256 kb) [file 12864_2016_3067_MOESM10_ESM.pdf]

Additional File 10: Overview of the BGCs in *M. anisopliae* E6, their domain structures, and conservation among six *Metarhizium* species.

**Cluster/ Backbone gene**	*MAR*	*MAC*	*MAM*	*MAJ*	*MGU*	*MBR*	**Product/Alt. names**	**Locus tag**	**48hCx48hI**	**48hIx144hI**	**Backbone gene domains**
MaNRPS1							Destruxin	MANI_024437	**Up**	**Down**	A | C A | E C A | C A | C A M | C A M | C
MaNRPS2							Serinocyclin	MANI_020119	**ND**	**Down**	A | C C A | C A | C A | C A | E C A | C A | E C A | C
MaNRPS3								MANI_011572	NA	NA	| C A | E C A |
MaNRPS4								MANI_020971	NA	NA	A | C A | E C
MaNRPS5								MANI_004659	NA	NA	| C A | C
MaNRPS6								MANI_004647	**ND**	**ND**	A | C A | C A | E C A | C A E C A | C A | E C A | C
MaNRPS7								MANI_010631	**Up**	**ND**	C A | C
MaNRPS8							Metachelin	MANI_003049	**Down**	NA	A | C | C
MaNRPS9							Ferricrocin	MANI_117325	**Up**	**ND**	C A C | C C
MaNRPS10								MANI_003902	NA	NA	A | C A | E C | C
MaNRPS11								MANI_006947	**ND**	**Down**	A | C A | C A | E C A | C A | E C A E C A | C A | C |
MaNRPS12								MANI_006155	NA	NA	A | E C A | C A | C A | E C
MaNRPS13								MANI_008843	NA	NA	A | C A | C A | C A |
MaPKS1							Tropolone /citrinin-related compound	MANI_014762	**Up**	**ND**	KS AT AC MT TD
MaPKS2							Aurovertin	MANI_004781	NA	NA	KS AT DH MT KR AC
MaPKS3								MANI_011575	NA	NA	KS AT DH AC TE
MaPKS4								MANI_110051	NA	NA	KS AT DH MT ER KR
*MaPKS5**								MANI_027199	NA	NA	KS DH AT DH
*MaPKS6**								MANI_010100	NA	NA	KS AT DH
MaPKS7								MANI_020125	NA	NA	KS AT DH MT ER KR AC
MaPKS8							MrPKS2	MANI_028434	NA	NA	KS AT DH AC AC TE
MaPKS9							Viridicatumtoxin	MANI_003768	NA	NA	KS AT AC
MaPKS10								MANI_019155	**Down**	**Down**	A AC KS AT KR AC TD
MaPKS11								MANI_006783	NA	NA	KS AT DH MT KR AC
MaPKS12								MANI_110978	NA	NA	KS AT DH MT ER KR
MaPKS13								MANI_006250	**ND**	**ND**	KS AT DH MT ER KR AC
MaPKS14								MANI_018879	**Up**	**Down**	KS AT DH AC

MaPKS15								MANI_021529	NA	NA	KS AT DH ER KR AC
MaPKS16								MANI_029455	NA	NA	KS AT DH ER KR AC
MaPKS17								MANI_025650	**Up**	**Down**	KS AT DH ER KR
MaPKS18								MANI_010451	**Up**	**Down**	KS AT AC
MaPKS19								MANI_012054	**ND**	**ND**	KS AT DH ER KR AC
MaPKS20							MrPKS1	MANI_122426	NA	NA	KS AT AC AC TE
MaPKS21								MANI_006397/<br>MANI_006388	NA	NA	ER KR AC DH ER
MaPKS22								MANI_017297	**ND**	**ND**	AT KS AC
MaNRPS-PKS1							NG39x	MANI_020791	**Up**	**Down**	KS AT DH MT KR AC C A TD
MaNRPS-PKS2							Pseurotin-related compound	MANI_018878	**Up**	**Down**	KS AT DH KR C A TD
MaNRPS-PKS3							Xenolozoyenone -related compound	MANI_023437	**Down**	**Down**	KS AT DH ER KR C A TD
MaNRPS-PKS4								MANI_022470/<br>MANI_022473	**ND**	**ND**	KS AT DH MT ER KR AC C A C
MaNRPS-PKS5								MANI_006158	NA	NA	KS AT DH MT KR C A
MaNRPS-PKS6								MANI_010456/<br>MANI_121659	NA	NA	A C A KS AT DH MT ER
MaNRPS-PKS7								MANI_001951/<br>MANI_001950	NA	NA	AT DH MT KR C A TD
MaTERP1							Helvolic acid	MANI_010532/<br>MANI_010527/<br>MANI_010530/<br>MANI_010531	**Up**	**Down**	
MaTERP2							Lanosterol cyclase	MANI_002110	**Up**	**ND**	
MaTERP3								MANI_027216	NA	NA	
MaTERP4								MANI_116096	**ND**	**ND**	
MaTERP5							GGPP synthase	MANI_024139	**ND**	**ND**	
MATERP6								MANI_020049	**ND**	**ND**	
MaTERP7								MANI_020253	NA	NA	
MaTERP8								MANI_027397	NA	NA	
MaTERP9								MANI_022959	**ND**	**Down**	
MaIND1								MANI_023817	**Up**	**Down**	
MaIND2								MANI_004342	NA	NA	
MaIND3								MANI_024963	NA	NA	
MaSID1								MANI_004303	NA	NA	
MaIND-TERP1							Terpendole E/ Lolitrem-related	MANI_011022	NA	NA	

							compound				
MaIND-NRPS1							Elymoclavine/ Ergovaline-related compound	MANI_029655	NA	NA	A | C TD
MaIND-NRPS2								MANI_122478	NA	NA	| C A | C
MaTERP-PKS1								MANI_006324	NA	NA	KS AT MT
MaOTHER1								MANI_007147	**Down**	**ND**	
MaOTHER2								MANI_109737	**ND**	**ND**	
MaOTHER3								MANI_004951	**ND**	**ND**	
MaOTHER4								MANI_019946	**ND**	**ND**	
MaOTHER5								MANI_018009	NA	NA	
MaOTHER6								MANI_117972	**ND**	**ND**	
MaOTHER7								MANI_000865	NA	NA	
MaOTHER8								MANI_000852	**Up**	**ND**	
MaOTHER9								MANI_024071	NA	NA	
MaOTHER10								MANI_011653	NA	NA	
MaOTHER11								MANI_026624	**Down**	**ND**	
MaOTHER12								MANI_010482	**Up**	**Down**	
MaOTHER13								MANI_025168	**Up**	**ND**	
MaOTHER14								MANI_122490	NA	NA	
**TOTAL= 73**	**69**	**35**	**30**	**61**	**64**	**70**					

A Adenylation	| Peptidyl carrier protein	AT Acyltransferase	ER Enoylreductase	TD Thioester reductase
C Condensation	M N-Methylation	AC Acyl carrier protein	MT Methyltransferase O- or C-	KR Ketoreductase
E Epimerization	KS Keto-synthase	DH Dehydratase	TE Thioesterase	

M. robertsii ARSEF23 (MAR), *M. acridum* CQMa102 (MAC), *M. album* ARSEF1941 (MAM), *M. majus* ARSEF297 (MAJ), *M. guizhouense* ARSEF977 (MGU) and *M. brunneum* ARSEF3297 (MBR). Known products as well as alternative names and locus tags of the backbone genes are also listed. Relative changes in expression levels were estimated after 48 hours for the control condition (hC) and both 48 and 144 hours for infection conditions (hI). NA: Not Available. ND: No Difference. * The backbone gene appears to be truncated.
