# Supplementary material for: Secondary metabolite gene clusters in the entomopathogen fungus Metarhizium anisopliae: genome identification and patterns of expression in a cuticle infection model
Source: BMC Genomics. 2016 Oct 25;17(Suppl 8):736. doi: 10.1186/s12864-016-3067-6 (PMC5088523; doi:10.1186/s12864-016-3067-6)
Supplement: Additional file 11: — Expression profile of predicted BGCs. (PDF 934 kb) [file 12864_2016_3067_MOESM11_ESM.pdf]

Additional File 11: Expression profile of predicted BGCs.

Table 1: Expression profiling of the MaNRPS2 cluster (Serinocyclin).

NCBI's gene locus ID	Expression (RPKM)			Differential expression (log2-fold change)	
	48hC	48hI	144hI	48hCx48hI	48hIx144hI
**MANI_020146**	39.27	20.86	16.60	NA	NA
**MANI_020153**	3.28	0.00	0.00	NA	NA
**MANI_020163**	83.85	2.50	13.55	-4.78	2.46
**MANI_020161**	0.00	0.90	2.74	NA	NA
**MANI_020136**	5.00	11.18	2.27	1.25	-2.21
**MANI_020119**	14.72	10.71	4.77	NA	-1.09
**MANI_115536**	0.00	7.42	0.00	NA	NA
**MANI_020154**	0.00	1.66	0.00	NA	NA
**MANI_020156**	0.88	0.00	0.00	NA	NA
**MANI_020147**	1.69	0.91	0.00	NA	NA
**MANI_020164**	0.00	1.92	0.00	NA	NA
**MANI_020155**	9.37	37.12	2.56	2.09	-3.75
**MANI_020139**	33.07	36.39	33.99	NA	NA
**MANI_020162**	24.84	15.84	8.47	NA	NA
**MANI_020151**	32.58	91.15	163.66	1.58	NA
**MANI_029315**	5.81	15.61	92.76	1.54	2.52

Table 2: Expression profiling of the MaOTHER1 cluster.

NCBI's gene locus ID	Expression (RPKM)			Differential expression (log2-fold change)	
	48hC	48hI	144hI	48hCx48hI	48hIx144hI
**MANI_007159**	17.87	34.95	64.73	NA	NA
**MANI_007149**	39.73	20.00	28.13	NA	NA
**MANI_026611**	96.44	23.48	18.35	-1.90	NA

**MANI_007147**	24.95	5.81	4.43	-1.99	NA
**MANI_026610**	9.99	10.45	10.62	NA	NA

The BGC boundaries were delimited by CASSIS.

Table 3: Expression profiling of the MaOTHER2 cluster.

NCBI's gene locus ID	Expression (RPKM)			Differential expression (log2-fold change)	
	48hC	48hI	144hI	48hCx48hI	48hIx144hI
**MANI_109737**	0.00	4.81	0.98	NA	NA
**MANI_007750**	0.00	0.00	0.84	NA	NA
**MANI_131937**	0.00	0.00	1.71	NA	NA
**MANI_007754**	136.01	214.67	220.69	NA	NA
**MANI_007753**	285.59	207.31	336.46	NA	NA

Table 4: Expression profiling of the MaNRPS-PKS3 cluster (Xenolozoyenone-related compound).

NCBI's gene locus ID	Expression (RPKM)			Differential expression (log2-fold change)	
	48hC	48hI	144hI	48hCx48hI	48hIx144hI
**MANI_023523**	40.29	10.67	4.07	-1.74	-1.35
**MANI_023437**	11.23	1.59	0.62	-2.68	-1.30
**MANI_030051**	6.43	6.62	4.10	NA	NA
**MANI_030058**	0.69	0.00	0.00	NA	NA
**MANI_023546**	4.74	3.82	1.67	NA	NA
**MANI_023568**	3.27	1.32	1.34	NA	NA
**MANI_023497**	0.00	0.00	0.00	NA	NA
**MANI_023527**	2.24	2.25	6.42	NA	1.48

The BGC boundaries were delimited by CASSIS.

Table 5: Expression profiling of the MaIND1 cluster.

NCBI's gene locus ID	Expression (RPKM)			Differential expression (log2-fold change)	
	48hC	48hI	144hI	48hCx48hI	48hIx144hI
**MANI_023817**	0.74	13.71	6.06	4.04	-1.13
**MANI_030140**	4.11	3.49	3.90	NA	NA
**MANI_113953**	39.08	0.95	1.45	-5.03	NA
**MANI_023814**	1.07	0.29	0.00	NA	NA

The BGC boundaries were delimited by CASSIS.

Table 6: Expression profiling of the MaPKS2 cluster (Aurovertin).

NCBI's gene locus ID	Expression (RPKM)			Differential expression (log2-fold change)	
	48hC	48hI	144hI	48hCx48hI	48hIx144hI
**MANI_004840**	0.00	0.00	0.00	NA	NA
**MANI_004781**	1.66	0.19	0.00	NA	NA
**MANI_114236**	0.00	0.00	0.00	NA	NA
**MANI_004795**	0.54	0.44	0.00	NA	NA
**MANI_004789**	2.46	3.30	2.01	NA	NA
**MANI_114238**	0.00	0.00	0.00	NA	NA
**MANI_124919**	0.00	0.00	0.00	NA	NA
**MANI_004822**	0.00	5.32	0.68	NA	NA
**MANI_004785**	0.00	0.00	0.00	NA	NA
**MANI_004787**	0.00	0.00	0.00	NA	NA
**MANI_026081**	9.41	1.17	0.30	-2.79	NA
**MANI_004841**	96.96	44.17	50.18	NA	NA
**MANI_004827**	3.27	3.30	6.04	NA	NA
**MANI_124927**	1.83	1.47	0.00	NA	NA
**MANI_004821**	1.52	0.00	0.62	NA	NA
**MANI_114246**	0.00	0.00	0.00	NA	NA
**MANI_004807**	68.73	28.78	12.16	-1.12	-1.21
**MANI_004844**	22.42	61.91	34.12	1.59	NA

**MANI_004776**	53.60	30.98	20.79	NA	NA
**MANI_004777**	22.95	6.88	8.48	-1.57	NA
**MANI_004778**	133.95	80.25	79.03	NA	NA

The BGC boundaries were delimited by CASSIS.

Table 7: Expression profiling of the MaNRPS3 cluster.

**NCBI's gene locus ID**	**Expression (RPKM)**			**Differential expression (log2-fold change)**	
	**48hC**	**48hI**	**144hI**	**48hCx48hI**	**48hIx144hI**
**MANI_027530**	0.00	0.00	0.00	NA	NA
**MANI_027532**	0.00	0.00	0.00	NA	NA
**MANI_011622**	0.00	0.00	0.00	NA	NA
**MANI_011572**	0.00	0.37	0.00	NA	NA
**MANI_027526**	0.00	1.09	0.19	NA	NA
**MANI_011609**	3.60	18.83	1.47	2.46	-3.51
**MANI_114418**	0.00	0.00	0.00	NA	NA
**MANI_011644**	0.00	0.00	0.00	NA	NA
**MANI_011593**	0.00	0.00	0.00	NA	NA

Table 8: Expression profiling of the MaPKS3 cluster.

**NCBI's gene locus ID**	**Expression (RPKM)**			**Differential expression (log2-fold change)**	
	**48hC**	**48hI**	**144hI**	**48hCx48hI**	**48hIx144hI**
**MANI_027534**	0.59	39.58	9.20	5.86	-2.05
**MANI_027538**	0.00	132.34	210.52	8.68	NA
**MANI_011630**	0.00	64.38	38.76	6.69	NA
**MANI_027536**	0.00	6.93	0.00	NA	NA
**MANI_011575**	0.68	0.22	0.22	NA	NA
**MANI_011615**	7.99	747.98	415.37	6.65	NA
**MANI_011585**	0.00	0.00	0.00	NA	NA

**MANI_011587**	0.00	0.00	0.00	NA	NA
**MANI_011645**	0.00	0.00	0.00	NA	NA
**MANI_011623**	0.98	0.79	0.00	NA	NA

Table 9: Expression profiling of the MaNRPS4 cluster.

NCBI's gene locus ID	Expression (RPKM)			Differential expression (log2-fold change)	
	**48hC**	**48hI**	**144hI**	**48hCx48hI**	**48hIx144hI**
**MANI_029521**	0.00	0.00	0.00	NA	NA
**MANI_021152**	2.00	130.76	239.85	5.82	0.97
**MANI_021040**	1.51	10.53	0.00	2.81	-6.68
**MANI_021047**	0.00	0.49	3.01	NA	NA
**MANI_021015**	0.83	2.33	4.40	NA	NA
**MANI_021096**	6.12	4.93	0.84	NA	NA
**MANI_029504**	8.13	14.32	5.41	NA	-1.39
**MANI_029514**	0.00	0.00	0.00	NA	NA
**MANI_021147**	3.57	0.00	0.00	NA	NA
**MANI_021114**	0.00	0.00	0.00	NA	NA
**MANI_021108**	0.00	0.00	0.00	NA	NA
**MANI_021072**	0.00	0.00	0.00	NA	NA
**MANI_021085**	1.73	0.70	0.00	NA	NA
**MANI_021143**	0.00	0.00	0.00	NA	NA
**MANI_021102**	0.00	0.00	0.00	NA	NA
**MANI_021077**	0.00	0.00	0.00	NA	NA
**MANI_020971**	0.51	0.17	0.00	NA	NA

The BGC boundaries were delimited by CASSIS.

Table 10: Expression profiling of the MaNRPS5 cluster.

NCBI's gene locus ID	Expression (RPKM)			Differential expression (log2-fold change)	

	48hC	48hI	144hI	48hCx48hI	48hIx144hI
**MANI_026060**	5.57	1.34	10.47	-1.90	2.83
**MANI_110048**	44.93	29.69	12.25	NA	-1.20
**MANI_026063**	0.00	0.00	0.00	NA	NA
**MANI_004685**	0.00	0.00	0.00	NA	NA
**MANI_004679**	12.37	0.00	0.00	NA	NA
**MANI_026064**	5.69	1.53	1.55	NA	NA
**MANI_004691**	6.25	0.00	1.02	NA	NA
**MANI_004698**	4.93	14.57	0.00	NA	NA
**MANI_004659**	0.87	0.14	0.28	NA	NA
**MANI_004676**	2.14	17.24	1.17	NA	NA
**MANI_004665**	0.00	0.00	0.00	NA	NA
**MANI_026062**	0.00	0.00	0.00	NA	NA
**MANI_004683**	0.00	0.00	0.00	NA	NA
**MANI_004696**	0.00	0.00	0.00	NA	NA
**MANI_004669**	0.00	0.00	0.00	NA	NA

The BGC boundaries were delimited by CASSIS.

Table 11: Expression profiling of the MaNRPS6 cluster.

NCBI's gene locus ID	Expression (RPKM)			Differential expression (log2-fold change)	
	48hC	48hI	144hI	48hCx48hI	48hIx144hI
**MANI_004647**	9.24	4.92	2.81	NA	NA
**MANI_026065**	28.51	8.83	1.79	NA	NA

The BGC boundaries were delimited by CASSIS.

Table 12: Expression profiling of the MaPKS4 cluster.

NCBI's gene locus ID	Expression (RPKM)			Differential expression (log2-fold change)	

	48hC	48hI	144hI	48hCx48hI	48hIx144hI
**MANI_029451**	0.00	0.00	0.00	NA	NA
**MANI_020788**	0.00	0.00	0.00	NA	NA
**MANI_020787**	0.00	1.14	0.00	NA	NA
**MANI_020781**	12.56	16.04	14.53	NA	NA
**MANI_020786**	0.00	0.00	0.00	NA	NA
**MANI_020789**	1.84	2.96	0.00	NA	NA
**MANI_110051**	0.23	0.09	0.19	NA	NA
**MANI_029463**	0.00	0.41	0.00	NA	NA
**MANI_020891**	0.00	0.00	0.00	NA	NA
**MANI_020915**	0.00	2.12	0.72	NA	NA
**MANI_020808**	2.70	8.25	9.71	NA	NA
**MANI_020807**	6.01	5.25	15.16	NA	1.51

Table 13: Expression profiling of the MaOTHER3 cluster.

NCBI's gene locus ID	Expression (RPKM)			Differential expression (log2-fold change)	
	48hC	48hI	144hI	48hCx48hI	48hIx144hI
**MANI_005013**	0.81	0.00	0.00	NA	NA
**MANI_005019**	6.30	4.44	0.00	NA	NA
**MANI_004943**	0.00	0.00	0.00	NA	NA
**MANI_005067**	21.84	0.00	0.00	NA	NA
**MANI_004951**	5.08	0.77	2.08	NA	NA
**MANI_026138**	0.71	0.00	0.00	NA	NA
**MANI_005008**	0.00	0.00	0.00	NA	NA
**MANI_004982**	0.00	0.00	0.00	NA	NA
**MANI_005015**	0.00	0.00	0.00	NA	NA
**MANI_005038**	0.00	0.00	0.00	NA	NA
**MANI_005061**	0.00	0.00	0.00	NA	NA

Table 14: Expression profiling of the MaTERP2 cluster (Lanosterol cyclase).

NCBI's gene locus ID	Expression (RPKM)			Differential expression (log2-fold change)	
	48hC	48hI	144hI	48hCx48hI	48hIx144hI
**MANI_025532**	6.98	5.62	18.85	NA	1.71
**MANI_002110**	27.78	65.85	123.06	1.37	NA
**MANI_002183**	28.69	22.06	16.01	NA	NA
**MANI_002164**	4.64	14.96	4.27	1.80	-1.73
**MANI_002157**	86.77	55.48	61.95	NA	NA
**MANI_002152**	62.42	20.46	20.34	-1.46	NA

The BGC boundaries were delimited by CASSIS.

Table 15: Expression profiling of the MaNRPS-PKS4 cluster.

NCBI's gene locus ID	Expression (RPKM)			Differential expression (log2-fold change)	
	48hC	48hI	144hI	48hCx48hI	48hIx144hI
**MANI_022500**	0.00	0.00	0.00	NA	NA
**MANI_029817**	8.86	0.59	5.44	NA	NA
**MANI_022499**	0.00	0.00	1.06	NA	NA
**MANI_022470**	7.87	4.92	1.25	NA	-1.95

The BGC boundaries were delimited by CASSIS.

Table 16: Expression profiling of the MaPKS5 cluster.

NCBI's gene locus ID	Expression (RPKM)			Differential expression (log2-fold change)	
	48hC	48hI	144hI	48hCx48hI	48hIx144hI
**MANI_010111**	0.00	0.00	0.00	NA	NA
**MANI_010152**	0.00	0.00	0.00	NA	NA
**MANI_010132**	2.88	2.32	0.79	NA	NA
**MANI_010133**	0.00	0.00	0.00	NA	NA

**MANI_010116**	0.00	0.00	0.71	NA	NA
**MANI_010145**	0.00	0.00	0.00	NA	NA
**MANI_010135**	0.00	0.00	2.31	NA	NA
**MANI_010096**	0.85	16.35	18.69	4.12	NA
**MANI_010114**	14.06	16.99	18.53	NA	NA
**MANI_027208**	0.28	0.23	0.00	NA	NA
**MANI_027199**	0.00	0.24	0.00	NA	NA
**MANI_115496**	0.00	0.00	0.00	NA	NA
**MANI_010167**	0.00	0.00	0.00	NA	NA
**MANI_010121**	0.00	0.00	0.00	NA	NA
**MANI_010120**	0.00	0.69	2.09	NA	NA
**MANI_010092**	0.00	0.00	0.00	NA	NA
**MANI_010168**	0.00	0.00	0.00	NA	NA
**MANI_010063**	0.74	0.00	0.00	NA	NA
**MANI_010140**	0.00	0.00	0.00	NA	NA
**MANI_027224**	0.00	1.25	0.00	NA	NA

Table 17: Expression profiling of the MaTERP3 cluster.

**NCBI's gene locus ID**	**Expression (RPKM)**			**Differential expression (log2-fold change)**	
	**48hC**	**48hI**	**144hI**	**48hCx48hI**	**48hIx144hI**
**MANI_027210**	0.00	0.00	0.00	NA	NA
**MANI_027216**	0.00	0.00	0.00	NA	NA
**MANI_010143**	3.46	1.86	10.37	NA	NA
**MANI_125771**	0.00	0.00	1.37	NA	NA
**MANI_115504**	0.00	0.00	0.00	NA	NA

Table 18: Expression profiling of the MaPKS6 cluster.

**NCBI's gene locus ID**	**Expression (RPKM)**			**Differential expression (log2-fold change)**	
	**48hC**	**48hI**	**144hI**	**48hCx48hI**	**48hIx144hI**

**MANI_115504**	0.00	0.00	0.00	NA	NA
**MANI_010070**	0.00	0.31	0.00	NA	NA
**MANI_010106**	0.00	0.00	0.00	NA	NA
**MANI_010080**	0.00	0.00	0.52	NA	NA
**MANI_027206**	0.00	0.00	0.00	NA	NA
**MANI_010100**	0.00	0.00	0.00	NA	NA
**MANI_115518**	0.00	0.00	0.00	NA	NA
**MANI_010062**	0.00	0.00	0.00	NA	NA
**MANI_027223**	0.00	0.00	0.00	NA	NA

Table 19: Expression profiling of the MaPKS7 cluster.

**NCBI's gene locus ID**	**Expression (RPKM)**			**Differential expression (log2-fold change)**	
	**48hC**	**48hI**	**144hI**	**48hCx48hI**	**48hIx144hI**
**MANI_020133**	1.32	12.52	51.84	3.33	2.00
**MANI_115550**	0.00	0.00	1.21	NA	NA
**MANI_020132**	0.00	0.00	0.00	NA	NA
**MANI_020152**	0.00	0.00	0.00	NA	NA
**MANI_020166**	0.00	0.00	0.00	NA	NA
**MANI_020125**	0.12	0.00	0.00	NA	NA
**MANI_020170**	0.00	0.00	0.00	NA	NA
**MANI_029300**	1.96	2.37	0.00	NA	NA
**MANI_020096**	0.00	0.00	0.00	NA	NA
**MANI_115552**	1.09	1.53	9.11	NA	2.57
**MANI_020099**	4.06	11.77	118.42	NA	3.35
**MANI_020114**	0.00	1.84	0.00	NA	NA
**MANI_029303**	0.00	0.00	0.00	NA	NA

The BGC boundaries were delimited by CASSIS.

Table 20: Expression profiling of the MaOTHER4 cluster.

NCBI's gene locus ID	Expression (RPKM)			Differential expression (log2-fold change)	
	48hC	48hI	144hI	48hCx48hI	48hIx144hI
**MANI_019978**	130.30	139.93	1338.01	NA	3.22
**MANI_019961**	14.31	18.73	10.98	NA	NA
**MANI_019967**	3.47	2.24	10.80	NA	2.22
**MANI_019956**	228.68	81.62	415.42	-1.35	2.26
**MANI_029277**	15.97	6.27	6.70	-1.23	NA
**MANI_029279**	5.81	1.25	0.32	NA	NA
**MANI_019946**	59.12	52.64	43.27	NA	NA
**MANI_019963**	66.63	32.66	58.30	NA	NA
**MANI_019984**	31.07	0.00	0.00	NA	NA
**MANI_019966**	0.80	0.00	0.00	NA	NA
**MANI_019955**	19.35	26.54	9.84	NA	-1.43
**MANI_029283**	0.00	0.00	0.00	NA	NA

Table 21: Expression profiling of the MaNRPS7 cluster.

NCBI's gene locus ID	Expression (RPKM)			Differential expression (log2-fold change)	
	48hC	48hI	144hI	48hCx48hI	48hIx144hI
**MANI_027353**	8.11	6.53	0.00	NA	NA
**MANI_010645**	17.79	17.30	12.08	NA	NA
**MANI_010631**	6.41	20.32	12.50	1.79	NA
**MANI_115821**	50.55	62.04	55.14	NA	NA
**MANI_010634**	33.25	36.79	55.35	NA	NA
**MANI_010656**	12.92	13.68	18.73	NA	NA

The BGC boundaries were delimited by CASSIS.

Table 22: Expression profiling of the MaTERP4 cluster.

NCBI's gene locus ID	Expression (RPKM)			Differential expression (log2-fold change)	

	48hC	48hI	144hI	48hCx48hI	48hIx144hI
**MANI_021634**	25.52	20.56	12.53	NA	NA
**MANI_021633**	5.19	7.32	13.81	NA	NA
**MANI_021625**	7.97	27.80	13.03	1.93	-1.05
**MANI_116096**	32.26	46.37	47.62	NA	NA
**MANI_021624**	2.34	4.71	0.96	NA	NA
**MANI_021637**	7.16	1.92	0.00	NA	NA

Table 23: Expression profiling of the MaPKS8 cluster (MrPKS2).

NCBI's gene locus ID	Expression (RPKM)			Differential expression (log2-fold change)	
	48hC	48hI	144hI	48hCx48hI	48hIx144hI
**MANI_028483**	0.00	0.00	0.00	NA	NA
**MANI_016007**	0.00	0.00	0.00	NA	NA
**MANI_116665**	0.00	1.72	0.00	NA	NA
**MANI_016082**	0.00	0.00	0.00	NA	NA
**MANI_028434**	1.24	1.22	0.22	NA	NA
**MANI_016169**	0.00	2.67	0.00	NA	NA
**MANI_016181**	1.59	6.41	0.00	NA	NA
**MANI_016065**	2.32	0.00	0.00	NA	NA
**MANI_110628**	0.00	2.70	0.00	NA	NA
**MANI_016229**	0.00	0.00	0.00	NA	NA
**MANI_016226**	0.00	0.00	0.00	NA	NA
**MANI_016231**	0.00	0.00	0.00	NA	NA

The BGC boundaries were delimited by CASSIS.

Table 24: Expression profiling of the MaOTHER5 cluster.

NCBI's gene locus ID	Expression (RPKM)			Differential expression (log2-fold change)	
	48hC	48hI	144hI	48hCx48hI	48hIx144hI

**MANI_116947**	236.85	15.37	9.15	-3.82	NA
**MANI_018049**	74.38	45.22	64.43	NA	NA
**MANI_028870**	132.40	21.86	20.18	-2.45	NA
**MANI_018046**	1.25	35.29	13.83	4.82	-1.34
**MANI_018106**	2.62	3.17	1.07	NA	NA
**MANI_028863**	16.63	10.42	1.51	NA	-2.67
**MANI_018104**	37.83	10.60	22.88	-1.66	1.13
**MANI_018111**	105.18	36.08	28.68	-1.37	NA
**MANI_018053**	44.82	12.58	9.44	-1.71	NA
**MANI_018009**	52.57	17.96	34.76	-1.42	1.01
**MANI_110662**	12.83	12.72	19.38	NA	NA
**MANI_017975**	15.21	8.31	11.11	NA	NA
**MANI_028845**	14.53	27.80	16.35	NA	NA
**MANI_028842**	97.33	60.97	71.89	NA	NA
**MANI_017970**	13.37	13.08	35.16	NA	1.48
**MANI_017982**	21.66	7.75	23.63	NA	1.58
**MANI_017985**	24.41	23.08	24.31	NA	NA

Table 25: Expression profiling of the MaNRPS8 cluster (Metachelin).

**NCBI's gene locus ID**	**Expression (RPKM)**			**Differential expression (log2-fold change)**	
	**48hC**	**48hI**	**144hI**	**48hCx48hI**	**48hIx144hI**
**MANI_003049**	5.59	1.72	0.54	-1.55	NA
**MANI_003143**	14.88	6.20	2.52	-1.16	NA
**MANI_025764**	3.83	0.00	0.00	NA	NA
**MANI_003177**	22.45	9.61	4.59	-1.10	-1.06
**MANI_003060**	5.47	5.11	3.94	NA	NA
**MANI_003059**	28.13	17.17	17.20	NA	NA
**MANI_003078**	12.29	16.20	15.08	NA	NA
**MANI_003226**	11.23	2.26	18.40	NA	NA
**MANI_003103**	12.66	13.78	11.22	NA	NA
**MANI_003099**	16.46	31.92	34.02	1.07	NA

The BGC boundaries were delimited by CASSIS.

Table 26: Expression profiling of the MaNRPS9 cluster (Ferricrocin).

NCBI's gene locus ID	Expression (RPKM)			Differential expression (log2-fold change)	
	48hC	48hI	144hI	48hCx48hI	48hIx144hI
**MANI_008252**	5.97	1.92	0.00	NA	NA
**MANI_127150**	15.49	22.86	18.33	NA	NA
**MANI_008246**	39.97	36.56	15.34	NA	-1.22
**MANI_117323**	17.81	44.82	39.16	1.42	NA
**MANI_117325**	10.64	28.26	21.38	1.51	NA
**MANI_008248**	2.57	6.21	3.36	NA	NA

The BGC boundaries were delimited by CASSIS.

Table 27: Expression profiling of the MaPKS9 cluster (Viridicatumtoxin).

NCBI's gene locus ID	Expression (RPKM)			Differential expression (log2-fold change)	
	48hC	48hI	144hI	48hCx48hI	48hIx144hI
**MANI_003872**	0.00	0.00	1.05	NA	NA
**MANI_003860**	0.00	0.00	0.00	NA	NA
**MANI_003846**	3.52	3.41	2.88	NA	NA
**MANI_003816**	7.62	6.14	3.12	NA	NA
**MANI_003811**	1.21	0.00	0.33	NA	NA
**MANI_003842**	0.00	0.00	0.00	NA	NA
**MANI_003768**	0.66	0.00	0.00	NA	NA
**MANI_003787**	3.60	1.45	0.00	NA	NA
**MANI_025884**	2.33	4.37	3.80	NA	NA
**MANI_003838**	23.66	1.03	1.05	NA	NA

The BGC boundaries were delimited by CASSIS.

Table 28: Expression profiling of the MaNRPS10 cluster.

NCBI's gene locus ID	Expression (RPKM)			Differential expression (log2-fold change)	
	48hC	48hI	144hI	48hCx48hI	48hIx144hI
**MANI_003979**	1.00	4.01	0.00	NA	NA
**MANI_003960**	7.40	2.98	0.61	NA	NA
**MANI_003945**	0.00	0.45	0.00	NA	NA
**MANI_003902**	0.17	0.87	0.34	NA	NA
**MANI_004003**	0.00	0.00	0.00	NA	NA
**MANI_003995**	0.00	1.43	0.00	NA	NA
**MANI_003990**	0.00	52.01	5.98	7.65	-3.02
**MANI_003985**	0.00	0.00	0.00	NA	NA
**MANI_003934**	0.70	0.57	0.00	NA	NA

The BGC boundaries were delimited by CASSIS.

Table 29: Expression profiling of the MaPKS10 cluster.

NCBI's gene locus ID	Expression (RPKM)			Differential expression (log2-fold change)	
	48hC	48hI	144hI	48hCx48hI	48hIx144hI
**MANI_019165**	299.73	28.05	6.75	-3.31	-1.98
**MANI_019170**	231.24	21.04	8.71	-3.34	-1.24
**MANI_019155**	262.76	22.90	8.86	-3.52	-1.34
**MANI_019168**	430.71	96.96	32.83	-2.12	-1.49

The BGC boundaries were delimited by CASSIS.

Table 30: Expression profiling of the MaOTHER6 cluster.

NCBI's gene locus ID	Expression (RPKM)	Differential expression (log2-fold change)

	48hC	48hI	144hI	48hCx48hI	48hIx144hI
**MANI_127795**	0.00	32.13	143.38	NA	2.15
**MANI_006107**	47.10	38.84	70.65	NA	NA
**MANI_006104**	3.96	1.60	0.81	NA	NA
**MANI_006102**	8.04	0.00	1.20	NA	NA
**MANI_110861**	2.65	3.56	1.45	NA	NA
**MANI_117972**	9.17	8.72	8.86	NA	NA
**MANI_006066**	2.22	12.50	9.98	NA	NA
**MANI_006043**	55.72	11.33	6.91	-2.17	NA
**MANI_006072**	10.62	62.02	11.95	2.62	-2.31
**MANI_127783**	5.46	5.49	4.47	NA	NA
**MANI_026371**	7.27	6.51	3.97	NA	NA
**MANI_006080**	0.00	0.00	0.00	NA	NA
**MANI_026382**	372.43	218.58	42.29	NA	-2.40
**MANI_026375**	6.64	14.97	9.23	1.26	NA

Table 31: Expression profiling of the MaOTHER7 cluster.

NCBI's gene locus ID	Expression (RPKM)			Differential expression (log2-fold change)	
	48hC	48hI	144hI	48hCx48hI	48hIx144hI
**MANI_000880**	0.00	0.00	0.00	NA	NA
**MANI_118212**	0.00	0.00	0.00	NA	NA
**MANI_000883**	7.91	4.25	3.59	NA	NA
**MANI_025256**	9.30	2.50	5.07	NA	NA
**MANI_000863**	5.37	1.44	0.73	NA	NA
**MANI_000865**	0.32	0.00	0.00	NA	NA

The BGC boundaries were delimited by CASSIS.

Table 32: Expression profiling of the MaOTHER8 cluster.

NCBI's gene locus ID	Expression (RPKM)	Differential expression (log2-fold

				Differential expression (log2-fold change)	
	48hC	48hI	144hI	48hCx48hI	48hIx144hI
**MANI_000878**	1012.15	626.02	73.40	NA	-3.14
**MANI_110901**	11.64	136.93	74.57	3.63	NA
**MANI_000858**	12.25	54.27	24.50	2.26	-1.13
**MANI_000852**	0.00	207.98	129.69	10.09	NA
**MANI_118224**	9.55	204.61	60.95	4.50	-1.78
**MANI_000859**	8.82	278.69	146.05	5.09	-1.00
**MANI_000861**	6.11	180.78	190.77	4.96	NA

The BGC boundaries were delimited by CASSIS.

Table 33: Expression profiling of the MaTERP-PKS1 cluster.

NCBI's gene locus ID	Expression (RPKM)			Differential expression (log2-fold change)	
	48hC	48hI	144hI	48hCx48hI	48hIx144hI
**MANI_118227**	0.00	0.00	0.00	NA	NA
**MANI_026441**	1.02	23.73	0.83	NA	NA
**MANI_006343**	0.00	0.00	0.00	NA	NA
**MANI_127977**	0.00	0.00	0.00	NA	NA
**MANI_006355**	0.00	0.00	1.46	NA	NA
**MANI_006367**	1.21	0.00	0.00	NA	NA
**MANI_006324**	0.00	0.22	0.00	NA	NA
**MANI_006356**	0.00	0.00	0.00	NA	NA
**MANI_006353**	0.00	0.00	0.00	NA	NA

The BGC boundaries were delimited by CASSIS.

Table 34: Expression profiling of the MaPKS11 cluster.

NCBI's gene locus ID	Expression (RPKM)	Differential expression (log2-fold change)

	48hC	48hI	144hI	48hCx48hI	48hIx144hI
**MANI_006805**	**13.56**	**6.95**	**7.07**	NA	NA
**MANI_006801**	17.98	12.35	9.08	NA	NA
**MANI_006859**	15.96	18.56	26.11	NA	NA
**MANI_006835**	32.66	26.31	18.96	NA	NA
**MANI_006858**	43.49	28.29	67.06	NA	1.26
**MANI_026542**	0.00	0.00	0.00	NA	NA
**MANI_006811**	0.00	0.55	0.00	NA	NA
**MANI_006808**	0.00	0.00	0.00	NA	NA
**MANI_006856**	0.00	0.00	0.00	NA	NA
**MANI_006827**	0.00	0.00	0.00	NA	NA
**MANI_006783**	0.93	0.37	0.00	NA	NA
**MANI_006831**	0.00	0.00	0.00	NA	NA
**MANI_006795**	0.79	0.63	0.00	NA	NA
**MANI_006836**	0.00	0.00	0.00	NA	NA
**MANI_118628**	1.60	1.72	1.31	NA	NA
**MANI_006798**	1.75	4.69	4.77	NA	NA

The BGC boundaries were delimited by CASSIS.

Table 35: Expression profiling of the MaPKS12 cluster.

NCBI's gene locus ID	Expression (RPKM)			Differential expression (log2-fold change)	
	48hC	48hI	144hI	48hCx48hI	48hIx144hI
**MANI_002711**	0.00	0.51	0.00	NA	NA
**MANI_002718**	1.15	0.00	0.00	NA	NA
**MANI_002715**	0.91	0.73	0.00	NA	NA
**MANI_002710**	1.58	0.85	2.16	NA	NA
**MANI_110978**	0.12	0.00	0.10	NA	NA

The BGC boundaries were delimited by CASSIS.

Table 36: Expression profiling of the MaTERP5 cluster (GGPP synthase).

NCBI's gene locus ID	Expression (RPKM)			Differential expression (log2-fold change)	
	48hC	48hI	144hI	48hCx48hI	48hIx144hI
**MANI_024154**	56.33	19.06	14.72	-1.42	NA
**MANI_024125**	30.14	45.84	60.37	NA	NA
**MANI_024126**	45.84	32.23	36.97	NA	NA
**MANI_024161**	42.10	33.90	31.26	NA	NA
**MANI_030188**	3.93	3.54	5.12	NA	NA
**MANI_030184**	0.48	0.39	1.57	NA	NA
**MANI_118863**	13.35	13.35	15.50	NA	NA
**MANI_024131**	19.90	29.06	41.22	NA	NA
**MANI_024139**	12.34	9.94	19.06	NA	NA
**MANI_024162**	6.06	1.63	0.00	NA	NA

The BGC boundaries were delimited by CASSIS.

Table 37: Expression profiling of the MaOTHER9 cluster.

NCBI's gene locus ID	Expression (RPKM)			Differential expression (log2-fold change)	
	48hC	48hI	144hI	48hCx48hI	48hIx144hI
**MANI_024160**	0.00	0.00	0.00	NA	NA
**MANI_024071**	0.00	0.22	0.00	NA	NA
**MANI_024095**	5.57	0.37	0.38	NA	NA
**MANI_024163**	19.00	13.70	4.09	NA	NA

The BGC boundaries were delimited by CASSIS.

Table 38: Expression profiling of the MaNRPS11 cluster.

NCBI's gene locus ID	Expression (RPKM)			Differential expression (log2-fold change)	

	48hC	48hI	144hI	48hCx48hI	48hIx144hI
**MANI_006972**	0.00	3.22	0.00	NA	NA
**MANI_006947**	10.67	7.60	1.95	NA	-1.97
**MANI_006962**	13.24	19.43	23.60	NA	NA
**MANI_006985**	14.84	15.21	32.00	NA	1.08
**MANI_026573**	6.23	4.02	0.00	NA	NA
**MANI_006959**	60.29	41.06	51.73	NA	NA
**MANI_006969**	11.71	3.14	1.60	NA	NA
**MANI_118897**	8.95	5.90	14.01	NA	1.31

The BGC boundaries were delimited by CASSIS.

Table 39: Expression profiling of the MaNRPS-PKS5 cluster.

NCBI's gene locus ID	Expression (RPKM)			Differential expression (log2-fold change)	
	48hC	48hI	144hI	48hCx48hI	48hIx144hI
**MANI_006242**	2.06	0.00	0.00	NA	NA
**MANI_128792**	0.00	0.00	0.00	NA	NA
**MANI_006238**	4.25	0.00	0.00	NA	NA
**MANI_006214**	2.36	0.95	0.00	NA	NA
**MANI_026405**	6.57	4.91	1.54	NA	NA
**MANI_006237**	9.55	0.00	0.00	NA	NA
**MANI_006185**	1.88	1.21	0.31	NA	NA
**MANI_006236**	5.57	0.00	0.00	NA	NA
**MANI_006158**	0.16	0.00	0.00	NA	NA
**MANI_006235**	0.00	0.00	0.00	NA	NA
**MANI_006219**	3.16	0.00	0.00	NA	NA
**MANI_026410**	0.00	0.00	0.00	NA	NA
**MANI_026395**	0.12	0.00	0.00	NA	NA

Table 40: Expression profiling of the MaNRPS12 cluster.

NCBI's gene locus ID	Expression (RPKM)			Differential expression (log2-fold change)	
	48hC	48hI	144hI	48hCx48hI	48hIx144hI
**MANI_006235**	0.00	0.00	0.00	NA	NA
**MANI_006219**	3.14	0.00	0.00	NA	NA
**MANI_026410**	0.00	0.00	0.00	NA	NA
**MANI_026395**	0.12	0.00	0.00	NA	NA
**MANI_006234**	0.00	0.00	0.00	NA	NA
**MANI_026399**	0.40	0.16	0.33	NA	NA
**MANI_006155**	0.38	0.04	0.04	NA	NA
**MANI_006174**	19.31	31.10	33.75	NA	NA
**MANI_006224**	2.22	2.98	2.42	NA	NA
**MANI_006172**	0.37	0.00	0.00	NA	NA
**MANI_119390**	0.00	0.46	0.00	NA	NA
**MANI_006208**	11.80	0.79	0.00	NA	NA

The BGC boundaries were delimited by CASSIS.

Table 41: Expression profiling of the MaPKS13 cluster.

NCBI's gene locus ID	Expression (RPKM)			Differential expression (log2-fold change)	
	48hC	48hI	144hI	48hCx48hI	48hIx144hI
**MANI_006263**	0.00	0.00	0.00	NA	NA
**MANI_006262**	21.65	6.60	4.31	-1.56	NA
**MANI_006250**	17.29	13.39	8.52	NA	NA
**MANI_006260**	15.58	14.53	6.04	NA	-1.22
**MANI_006261**	77.75	3.89	1.80	-4.20	NA
**MANI_111163**	68.91	73.35	79.32	NA	NA

The BGC boundaries were delimited by CASSIS.

Table 42: Expression profiling of the MaIND-TERP1 cluster (Terpendole E/lolitrem-related compound).

NCBI's gene locus ID	Expression (RPKM)			Differential expression (log2-fold change)	
	48hC	48hI	144hI	48hCx48hI	48hIx144hI
**MANI_027395**	1.49	6.01	8.54	NA	NA
**MANI_027395**	1.49	6.01	8.54	NA	NA
**MANI_111286**	0.00	7.49	1.38	NA	NA
**MANI_027412**	0.00	0.00	0.00	NA	NA
**MANI_027413**	0.00	0.00	0.00	NA	NA
**MANI_119883**	1.37	2.21	13.49	NA	2.60
**MANI_011022**	0.00	0.00	0.00	NA	NA
**MANI_011010**	0.00	0.00	0.74	NA	NA
**MANI_011011**	0.00	0.00	0.00	NA	NA
**MANI_011024**	0.87	0.00	0.00	NA	NA
**MANI_119895**	0.00	0.00	0.00	NA	NA
**MANI_011015**	0.00	0.00	0.56	NA	NA
**MANI_027409**	0.00	0.00	0.00	NA	NA
**MANI_011023**	0.00	1.23	0.62	NA	NA

Table 43: Expression profiling of the MaNRPS13 cluster.

NCBI's gene locus ID	Expression (RPKM)			Differential expression (log2-fold change)	
	48hC	48hI	144hI	48hCx48hI	48hIx144hI
**MANI_008887**	87.51	40.93	72.24	NA	NA
**MANI_008916**	34.75	12.44	14.76	-1.29	NA
**MANI_008932**	50.81	57.28	47.19	NA	NA
**MANI_008883**	3.06	3.95	5.01	NA	NA
**MANI_026956**	23.71	37.62	34.69	NA	NA
**MANI_026948**	0.37	1.20	1.22	NA	NA
**MANI_008854**	0.00	4.49	0.00	NA	NA
**MANI_008852**	0.86	0.00	0.00	NA	NA
**MANI_008843**	0.15	0.00	0.00	NA	NA
**MANI_008835**	0.00	0.00	0.00	NA	NA

**MANI_008820**	3.25	5.57	1.00	NA	NA

The BGC boundaries were delimited by CASSIS.

Table 44: Expression profiling of the MaPKS14 cluster.

**NCBI's gene locus ID**	**Expression (RPKM)**			**Differential expression (log2-fold change)**	
	**48hC**	**48hI**	**144hI**	**48hCx48hI**	**48hIx144hI**
**MANI_018961**	0.00	6.48	0.00	NA	NA
**MANI_029060**	0.92	0.74	0.75	NA	NA
**MANI_018925**	0.00	1.95	0.49	NA	NA
**MANI_018949**	0.99	7.15	1.61	NA	NA
**MANI_018915**	0.51	7.81	7.93	NA	NA
**MANI_018966**	2.15	6.91	3.51	NA	NA
**MANI_018879**	2.82	6.81	2.58	1.42	-1.37

Table 45: Expression profiling of the MaIND-NRPS1 cluster (Elymoclavine/ergovaline-related compound).

**NCBI's gene locus ID**	**Expression (RPKM)**			**Differential expression (log2-fold change)**	
	**48hC**	**48hI**	**144hI**	**48hCx48hI**	**48hIx144hI**
**MANI_021859**	0.00	0.00	0.00	NA	NA
**MANI_029655**	0.00	0.00	0.00	NA	NA
**MANI_021853**	0.00	0.00	0.00	NA	NA
**MANI_029666**	0.00	0.00	0.00	NA	NA
**MANI_029677**	0.00	0.00	0.00	NA	NA
**MANI_021832**	2.48	0.50	0.51	NA	NA
**MANI_021880**	0.00	0.00	0.00	NA	NA
**MANI_120630**	0.39	1.26	0.00	NA	NA

The BGC boundaries were delimited by CASSIS.

Table 46: Expression profiling of the MaOTHER10 cluster.

NCBI's gene locus ID	Expression (RPKM)			Differential expression (log2-fold change)	
	48hC	48hI	144hI	48hCx48hI	48hIx144hI
**MANI_011655**	7.77	6.57	10.81	NA	NA
**MANI_011669**	10.08	13.19	5.15	NA	-1.33
**MANI_011674**	6.85	1.38	0.70	NA	NA
**MANI_011690**	0.00	112.28	107.00	8.53	NA
**MANI_011679**	1.18	1.43	19.78	NA	3.68
**MANI_011680**	5.09	8.72	59.38	NA	2.75
**MANI_011687**	14.26	58.09	22.64	2.14	-1.32
**MANI_011653**	0.00	0.00	0.00	NA	NA

Table 47: Expression profiling of the MaPKS15 cluster.

NCBI's gene locus ID	Expression (RPKM)			Differential expression (log2-fold change)	
	48hC	48hI	144hI	48hCx48hI	48hIx144hI
**MANI_021560**	6.78	4.96	19.70	NA	1.93
**MANI_021544**	0.00	3.49	0.00	NA	NA
**MANI_021563**	16.13	1022.48	19.79	6.09	-5.64
**MANI_021550**	6.02	16.57	44.34	1.58	1.42
**MANI_021547**	2.17	10.06	12.00	2.27	NA
**MANI_021569**	1.26	1.01	1.03	NA	NA
**MANI_021559**	0.60	2.92	0.00	NA	NA
**MANI_029610**	0.73	0.00	1.80	NA	NA
**MANI_021580**	0.00	0.00	0.00	NA	NA
**MANI_021529**	0.98	0.79	0.20	NA	NA

The BGC boundaries were delimited by CASSIS.

Table 48: Expression profiling of the MaPKS16 cluster.

NCBI's gene locus ID	Expression (RPKM)	Differential expression (log2-fold change)

	48hC	48hI	144hI	48hCx48hI	48hIx144hI
**MANI_020896**	0.00	0.00	0.00	NA	NA
**MANI_029455**	0.24	0.19	0.00	NA	NA
**MANI_020910**	0.00	0.00	0.00	NA	NA
**MANI_020904**	0.00	0.00	0.00	NA	NA
**MANI_121029**	0.00	0.57	0.58	NA	NA
**MANI_020797**	0.00	0.00	0.00	NA	NA

The BGC boundaries were delimited by CASSIS.

Table 49: Expression profiling of the MaOTHER11 cluster.

NCBI's gene locus ID	Expression (RPKM)			Differential expression (log2-fold change)	
	48hC	48hI	144hI	48hCx48hI	48hIx144hI
**MANI_121318**	32.80	17.12	10.18	NA	NA
**MANI_007223**	10.35	18.63	17.43	NA	NA
**MANI_007317**	19.62	6.70	16.05	-1.36	1.25
**MANI_121326**	60.49	25.00	29.63	-1.15	NA
**MANI_026683**	157.67	74.92	48.26	NA	NA
**MANI_111648**	86.50	52.01	42.86	NA	NA
**MANI_026624**	12.59	3.59	3.49	-1.68	NA
**MANI_007232**	41.21	26.04	26.71	NA	NA
**MANI_121338**	46.45	14.76	12.15	-1.53	NA
**MANI_121345**	22.65	16.91	46.09	NA	1.49
**MANI_136963**	22.73	12.20	12.42	NA	NA
**MANI_007164**	17.88	26.56	25.27	NA	NA
**MANI_130057**	39.08	15.38	12.52	-1.24	NA

Table 50: Expression profiling of the MaNRPS-PKS7 cluster.

NCBI's gene locus ID	Expression (RPKM)	Differential expression (log2-fold change)

	48hC	48hI	144hI	48hCx48hI	48hIx144hI
**MANI_002005**	0.00	0.00	0.00	NA	NA
**MANI_001951**	0.22	0.17	0.71	NA	NA
**MANI_001950**	0.00	0.16	0.00	NA	NA
**MANI_121458**	0.00	0.00	0.00	NA	NA

The BGC boundaries were delimited by CASSIS.

Table 51: Expression profiling of the MaPKS17 cluster.

NCBI's gene locus ID	Expression (RPKM)			Differential expression (log2-fold change)	
	48hC	48hI	144hI	48hCx48hI	48hIx144hI
**MANI_002833**	0.00	0.00	0.00	NA	NA
**MANI_002790**	0.00	0.00	0.00	NA	NA
**MANI_002789**	13.10	5.63	10.00	NA	NA
**MANI_002808**	0.00	3.00	1.52	NA	NA
**MANI_025650**	1.54	5.05	1.58	1.75	-1.55
**MANI_002819**	0.80	13.61	2.63	NA	NA
**MANI_002768**	1.37	23.87	9.65	4.22	-1.32
**MANI_002813**	0.84	0.67	0.00	NA	NA
**MANI_002850**	2.43	1.95	0.00	NA	NA
**MANI_002828**	0.00	0.00	0.00	NA	NA

The BGC boundaries were delimited by CASSIS.

Table 52: Expression profiling of the MaOTHER12 cluster.

NCBI's gene locus ID	Expression (RPKM)			Differential expression (log2-fold change)	
	48hC	48hI	144hI	48hCx48hI	48hIx144hI
**MANI_010565**	0.00	0.00	0.00	NA	NA
**MANI_010519**	83.27	258.33	140.03	1.75	NA

**MANI_010516**	0.00	11.80	1.89	NA	NA
**MANI_010482**	0.59	8.37	2.43	3.81	-1.74
**MANI_010462**	1.05	16.85	3.42	NA	NA
**MANI_010460**	0.00	2.70	0.00	NA	NA
**MANI_010583**	1.91	65.38	35.94	5.09	NA
**MANI_027314**	0.54	6.06	3.08	NA	NA
**MANI_010548**	0.78	69.21	56.88	6.31	NA
**MANI_027320**	0.00	21.77	7.04	7.38	-1.66

The BGC boundaries were delimited by CASSIS.

Table 53: Expression profiling of the MaPKS18 cluster.

NCBI's gene locus ID	Expression (RPKM)			Differential expression (log2-fold change)	
	**48hC**	**48hI**	**144hI**	**48hCx48hI**	**48hIx144hI**
**MANI_027325**	279.12	15.82	12.19	-4.04	NA
**MANI_010559**	0.00	0.00	0.00	NA	NA
**MANI_010451**	2.14	296.21	8.90	7.21	-5.01
**MANI_010561**	7.70	427.29	28.00	5.90	-3.98
**MANI_027340**	1.85	44.71	0.00	4.43	-6.89
**MANI_010595**	5.59	243.85	4.57	5.52	-5.65
**MANI_010577**	0.91	237.72	14.91	7.86	-3.95
**MANI_010494**	5.23	394.22	2.44	6.35	-7.27
**MANI_010469**	0.00	35.23	1.38	8.20	-4.52
**MANI_027305**	0.00	60.56	6.04	8.75	-3.32
**MANI_121654**	0.83	105.53	15.61	6.85	-2.78
**MANI_010508**	2.57	456.62	2.10	7.54	-7.67
**MANI_010524**	0.65	216.07	1.59	8.20	-6.92
**MANI_010603**	7.89	1844.05	101.19	7.94	-4.13
**MANI_121656**	0.00	179.64	3.23	8.75	-5.62

The BGC boundaries were delimited by CASSIS.

Table 54: Expression profiling of the MaNRPS-PKS6 cluster.

NCBI's gene locus ID	Expression (RPKM)			Differential expression (log2-fold change)	
	48hC	48hI	144hI	48hCx48hI	48hIx144hI
**MANI_010576**	0.00	0.72	0.00	NA	NA
**MANI_010456**	0.00	0.00	0.00	NA	NA
**MANI_121659**	0.00	0.00	0.00	NA	NA

The BGC boundaries were delimited by CASSIS.

Table 55: Expression profiling of the MaPKS19 cluster.

NCBI's gene locus ID	Expression (RPKM)			Differential expression (log2-fold change)	
	48hC	48hI	144hI	48hCx48hI	48hIx144hI
**MANI_012057**	32.23	35.24	28.70	NA	NA
**MANI_012116**	35.65	16.36	21.78	NA	NA
**MANI_012094**	24.60	6.60	26.09	-1.74	2.01
**MANI_012096**	25.33	16.58	66.06	NA	1.85
**MANI_012206**	2.03	4.08	5.81	NA	NA
**MANI_121697**	10.57	57.48	12.98	2.50	-2.05
**MANI_130428**	0.00	0.82	0.00	NA	NA
**MANI_012054**	2.89	4.95	7.29	NA	NA
**MANI_012122**	34.25	11.69	14.72	-1.46	NA

The BGC boundaries were delimited by CASSIS.

Table 56: Expression profiling of the MATERP6 cluster.

NCBI's gene locus ID	Expression (RPKM)			Differential expression (log2-fold change)	
	48hC	48hI	144hI	48hCx48hI	48hIx144hI

**MANI_020045**	4.98	9.12	10.37	NA	NA
**MANI_020046**	9.79	8.35	11.31	NA	NA
**MANI_020049**	1.81	3.65	8.89	NA	NA
**MANI_020053**	58.05	21.51	35.15	-1.30	NA

The BGC boundaries were delimited by CASSIS.

Table 57: Expression profiling of the MaOTHER13 cluster.

**NCBI's gene locus ID**	**Expression (RPKM)**			**Differential expression (log2-fold change)**	
	**48hC**	**48hI**	**144hI**	**48hCx48hI**	**48hIx144hI**
**MANI_000542****	23.17	48.52	50.97	1.14	NA
**MANI_025166****	2.95	3.57	0.60	NA	NA
**MANI_000548****	0.00	0.00	0.00	NA	NA
**MANI_000544****	0.00	8.10	0.00	NA	NA
**gene fragmented between two contigs**	X	X	X		
**MANI_000554*****	4.34	23.00	31.48	2.51	NA
**MANI_025168*****	0.18	7.82	10.94	5.23	NA
**MANI_000583******	8.03	9.70	1.64	NA	-2.43
**MANI_000620******	16.39	7.92	10.73	NA	NA
**MANI_000601******	17.74	6.90	19.52	-1.24	1.47
**MANI_000573******	3.17	2.98	8.20	NA	1.48
**MANI_000623******	0.00	0.00	0.00	NA	NA
**MANI_000569******	0.00	0.00	0.00	NA	NA

**contig00046 ***contig00234 ****contig00235

The BGC boundaries were delimited by comparative genomic.

Table 58: Expression profiling of the MaIND2 cluster.

NCBI's gene locus ID	Expression (RPKM)			Differential expression (log2-fold change)	
	48hC	48hI	144hI	48hCx48hI	48hIx144hI
**MANI_004366**	23.11	1503.88	224.45	6.15	-2.69
**MANI_004339**	8.07	6.99	2.03	NA	NA
**MANI_004371**	4.99	18.77	23.19	NA	NA
**MANI_111866**	27.97	55.51	49.94	1.11	NA
**MANI_004296**	603.43	29.95	15.52	-4.23	NA
**MANI_004325**	6.92	4.71	2.18	NA	NA
**MANI_004330**	62.93	283.97	119.47	2.30	-1.23
**MANI_004315**	149.01	74.86	43.86	NA	NA
**MANI_004356**	17.98	86.01	27.68	2.38	-1.66
**MANI_004327**	1.15	26.91	25.45	4.51	NA
**MANI_004359**	0.00	0.00	0.00	NA	NA
**MANI_004342**	0.00	2.15	0.00	NA	NA
**MANI_004337**	2.88	2.32	1.88	NA	NA
**MANI_025992**	3.17	0.85	0.00	NA	NA
**MANI_122213**	6.38	7.19	2.09	NA	NA
**MANI_025981**	4.80	3.48	2.36	NA	NA

The BGC boundaries were delimited by CASSIS.

Table 59: Expression profiling of the MaSID1 cluster.

NCBI's gene locus ID	Expression (RPKM)			Differential expression (log2-fold change)	
	48hC	48hI	144hI	48hCx48hI	48hIx144hI
**MANI_004337**	2.88	2.32	1.88	NA	NA
**MANI_025992**	3.17	0.85	0.00	NA	NA
**MANI_122213**	6.38	7.19	2.09	NA	NA
**MANI_025981**	4.80	3.48	2.36	NA	NA
**MANI_025985**	5.19	2.09	6.38	NA	NA
**MANI_122223**	3.64	0.00	0.00	NA	NA
**MANI_004345**	13.90	16.23	7.97	NA	NA

**MANI_025995**	34.27	23.91	9.36	NA	NA
**MANI_004301**	0.00	0.00	0.00	NA	NA
**MANI_004302**	0.00	0.00	0.00	NA	NA
**MANI_004303**	0.00	0.00	0.00	NA	NA
**MANI_025984**	0.00	0.00	0.00	NA	NA

The BGC boundaries were delimited by CASSIS.

Table 60: Expression profiling of the MaTERP7 cluster.

**NCBI's gene locus ID**	**Expression (RPKM)**			**Differential expression (log2-fold change)**	
	**48hC**	**48hI**	**144hI**	**48hCx48hI**	**48hIx144hI**
**MANI_020268**	7.86	0.00	1.07	**NA**	**NA**
**MANI_020207**	0.59	0.48	0.00	**NA**	**NA**
**MANI_020272**	0.00	0.00	0.00	NA	NA
**MANI_020253**	0.00	0.00	0.00	NA	NA
**MANI_020226**	3.26	1.97	1.33	NA	NA
**MANI_130866**	1.39	0.00	0.00	NA	NA
**MANI_020201**	3.17	22.04	15.93	2.86	NA

The BGC boundaries were delimited by CASSIS.

Table 61: Expression profiling of the MaPKS20 cluster (MrPKS1).

**NCBI's gene locus ID**	**Expression (RPKM)**			**Differential expression (log2-fold change)**	
	**48hC**	**48hI**	**144hI**	**48hCx48hI**	**48hIx144hI**
**MANI_122426**	0.43	0.11	0.00	NA	NA
**MANI_030118**	0.00	0.00	0.00	NA	NA
**MANI_122440**	0.00	0.00	0.00	NA	NA
**MANI_024459**	0.00	0.00	0.00	NA	NA

The BGC boundaries were delimited by CASSIS.

Table 62: Expression profiling of the MaPKS20 cluster.

NCBI's gene locus ID	Expression (RPKM)			Differential expression (log2-fold change)	
	48hC	48hI	144hI	48hCx48hI	48hIx144hI
**MANI_009961**	0.00	0.00	0.00	NA	NA
**MANI_009911**	0.00	0.00	0.00	NA	NA
**MANI_009948**	0.00	0.00	0.00	NA	NA
**MANI_009931**	0.00	0.00	0.00	NA	NA
**MANI_122478**	0.33	0.00	0.00	NA	NA

The BGC boundaries were delimited by CASSIS.

Table 63: Expression profiling of the MaOTHER14 cluster.

NCBI's gene locus ID	Expression (RPKM)			Differential expression (log2-fold change)	
	48hC	48hI	144hI	48hCx48hI	48hIx144hI
**MANI_009929**	8.46	2.62	0.00	NA	NA
**MANI_027167**	0.00	0.00	0.00	NA	NA
**MANI_009917**	0.00	0.00	0.00	NA	NA
**MANI_122490**	0.60	0.00	0.00	NA	NA
**MANI_027170**	0.62	0.00	1.01	NA	NA
**MANI_009926**	1.56	0.00	0.00	NA	NA
**MANI_009964**	0.00	0.00	0.00	NA	NA

The BGC boundaries were delimited by CASSIS.

Table 64: Expression profiling of the MaTERP8 cluster.

NCBI's gene locus ID	Expression (RPKM)	Differential expression (log2-fold

				change)	
	48hC	48hI	144hI	48hCx48hI	48hIx144hI
**MANI_010996**	0.00	142.69	29.99	8.44	-2.19
**MANI_027397**	0.00	0.00	0.00	NA	NA
**MANI_010966**	12.38	17.56	15.94	NA	NA
**MANI_010962**	1.85	1.46	3.03	NA	NA
**MANI_010993**	0.00	0.00	0.00	NA	NA
**MANI_010967**	0.00	0.00	0.54	NA	NA
**MANI_027387**	10.14	11.92	11.13	NA	NA
**MANI_027402**	3.12	6.71	6.83	NA	NA
**MANI_119837**	1.88	0.00	1.54	NA	NA

The BGC boundaries were delimited by CASSIS.

Table 65: Expression profiling of the MaPKS21 cluster.

NCBI's gene locus ID	Expression (RPKM)			Differential expression (log2-fold change)	
	48hC	48hI	144hI	48hCx48hI	48hIx144hI
**MANI_110367**	0.00	0.33	0.00	NA	NA
**MANI_006393**	0.00	0.00	0.00	NA	NA
**MANI_006390**	0.00	2.05	0.00	NA	NA
**MANI_006399**	0.70	0.00	0.00	NA	NA
**MANI_006397**	0.00	0.42	0.00	NA	NA
**MANI_006388**	1.13	0.61	0.00	NA	NA
**MANI_006380**	4.53	0.37	0.93	-3.34	NA
**MANI_025247**	13.04	7.88	8.01	NA	NA
**MANI_110374**	16.10	4.82	1.23	-1.61	NA
**MANI_025248**	0.94	0.75	1.53	NA	NA
**MANI_000835**	10.20	58.41	32.51	2.63	NA

The BGC boundaries were delimited by CASSIS.

Table 66: Expression profiling of the MaPKS22 cluster.

NCBI's gene locus ID	Expression (RPKM)			Differential expression (log2-fold change)	
	48hC	48hI	144hI	48hCx48hI	48hIx144hI
**MANI_017305**	11.98	4.70	2.27	-1.21	NA
**MANI_028697**	32.25	19.25	11.85	NA	NA
**MANI_017308**	44.68	42.33	54.92	NA	NA
**MANI_028694**	9.95	7.16	1.17	NA	NA
**MANI_017329**	5.44	0.00	0.64	NA	NA
**MANI_017326**	14.64	33.41	15.00	1.34	-1.17
**MANI_028690**	100.76	59.02	47.20	NA	NA
**MANI_017297**	76.21	53.97	58.30	NA	NA
**MANI_028699**	16.10	7.98	15.22	NA	1.01
**MANI_017319**	28.36	15.67	19.14	NA	NA
**MANI_014258**	0.00	0.00	0.00	NA	NA

The BGC boundaries were delimited by CASSIS.

Table 67: Expression profiling of the MaTERP9 cluster.

NCBI's gene locus ID	Expression (RPKM)			Differential expression (log2-fold change)	
	48hC	48hI	144hI	48hCx48hI	48hIx144hI
**MANI_022954**	5.42	0.55	1.67	NA	NA
**MANI_022919**	19.28	6.79	2.57	-1.38	-1.39
**MANI_029935**	19.16	23.51	9.72	NA	-1.20
**MANI_022959**	11.49	12.03	5.18	NA	-1.15
**MANI_022941**	2.43	18.77	6.76	3.04	-1.44
**MANI_022963**	4.64	9.60	6.51	NA	NA
**MANI_022973**	0.00	4.29	1.45	NA	NA

The BGC boundaries were delimited by CASSIS.

Table 68: Expression profiling of the MaIND3 cluster.

NCBI's gene locus ID	Expression (RPKM)			Differential expression (log2-fold change)	
	48hC	48hI	144hI	48hCx48hI	48hIx144hI
**MANI_024998**	1.92	0.77	2.36	NA	NA
**MANI_030435**	0.00	0.00	0.00	NA	NA
**MANI_024979**	0.00	0.00	0.00	NA	NA
**MANI_024963**	2.58	0.26	2.90	NA	NA
**MANI_030430**	3.77	0.00	1.93	NA	NA
**MANI_024985**	1.68	0.90	3.22	NA	NA
**MANI_112059**	25.12	17.53	18.52	NA	NA
**MANI_024957**	2.32	22.89	6.89	3.42	-1.69

For all tables, forty-eight hours for control condition (hC) and both 48 and 144 hours for infection condition (hI) were used to estimate the relative changes in expression levels. NA: Not Available.
