# Supplementary material for: Secondary metabolite gene clusters in the entomopathogen fungus Metarhizium anisopliae: genome identification and patterns of expression in a cuticle infection model
Source: BMC Genomics. 2016 Oct 25;17(Suppl 8):736. doi: 10.1186/s12864-016-3067-6 (PMC5088523; doi:10.1186/s12864-016-3067-6)
Supplement: Additional file 12: — Comparative genomic analysis and synteny for the aurovertin, elymoclavine/ergovaline-related compound, terpendole E/lolitrem-related compound, and xenolozoyenone-related compound BGCs. (PDF 161 kb) [file 12864_2016_3067_MOESM12_ESM.pdf]

Additional File 12: Comparative genomic analysis and synteny for aurovertin, elymoclavine/ergovaline related compound, terpendole E/lolitrem related compound, and xenolozoyenone related compound BGCs.

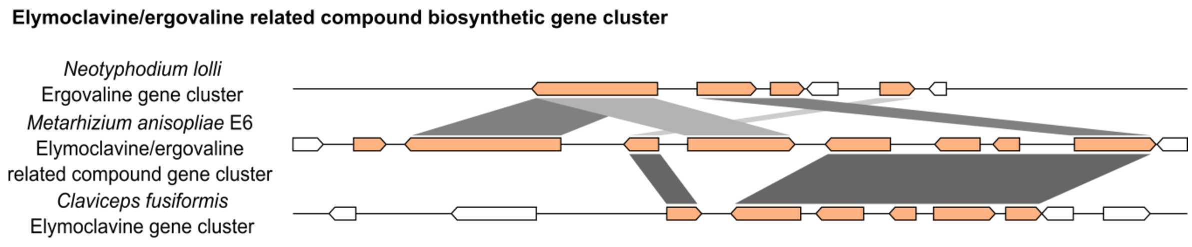

Figure 1: Comparative genomic analysis and synteny for elymoclavine/ergovaline related compound BGC (MaIND-NRPS1).

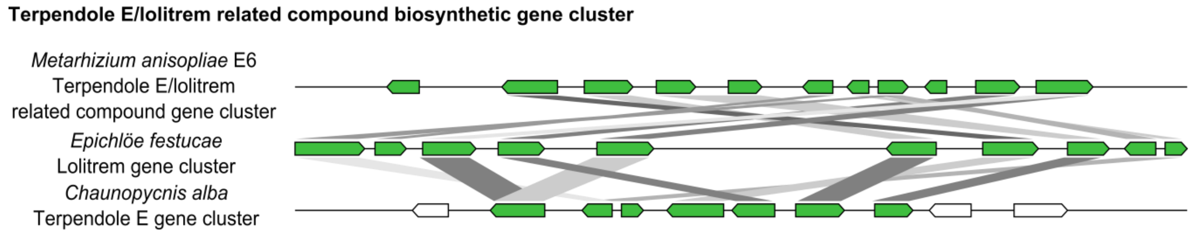

Figure 2: Comparative genomic analysis and synteny for terpendole E/lolitrem related compound BGC (MaIND-TERP1).

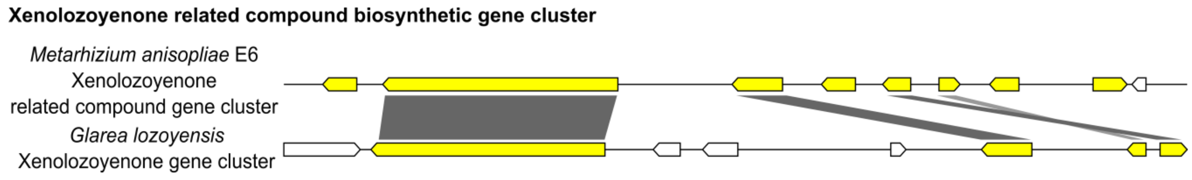

Figure 3: Comparative genomic analysis and synteny for xenolozoyenone related compound BGC (MaNRPS-PKS3).

Table 1*: Identity between aurovertin BGC from *C. arbuscula* and the putative aurovertin BGC (MaPKS2) from *M. anisopliae*.

C. arbuscula **gene**	***M. anisopliae*** **gene**	**Identity**
aurA	MANI_004781	77%
aurB	MANI_004840	76 %
aurC	MANI_026083	77 %
aurD	MANI_026090	71 %
aurE	MANI_026098	53 %
aurF	MANI_120612	40%
aurG	x	x

*No nucleotide sequence found for aurovertin BGC from *C. arbuscula,* not allowing synteny analysis.
