# Supplementary material for: Secondary metabolite gene clusters in the entomopathogen fungus Metarhizium anisopliae: genome identification and patterns of expression in a cuticle infection model
Source: BMC Genomics. 2016 Oct 25;17(Suppl 8):736. doi: 10.1186/s12864-016-3067-6 (PMC5088523; doi:10.1186/s12864-016-3067-6)
Supplement: Additional file 13: — Additional phylogenetic trees. (PDF 463 kb) [file 12864_2016_3067_MOESM13_ESM.pdf]

Additional File 13: Additional phylogenetic trees.

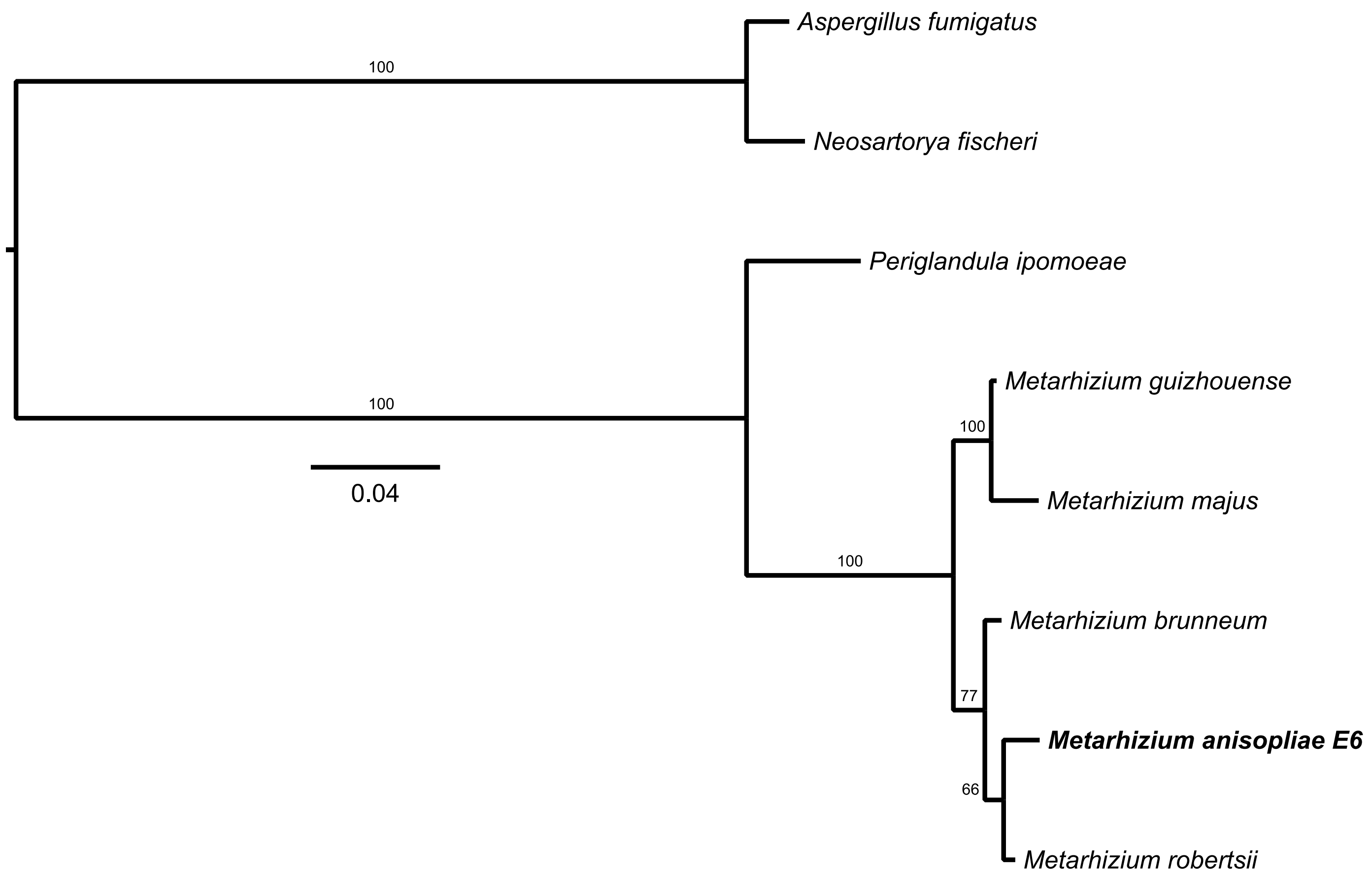

Figure 1: Maximum-likelihood tree for MANI_010495. The gene MANI_010495 assists in helvolic acid biosynthesis. Branches support values (bootstrap proportions) are associated with nodes.

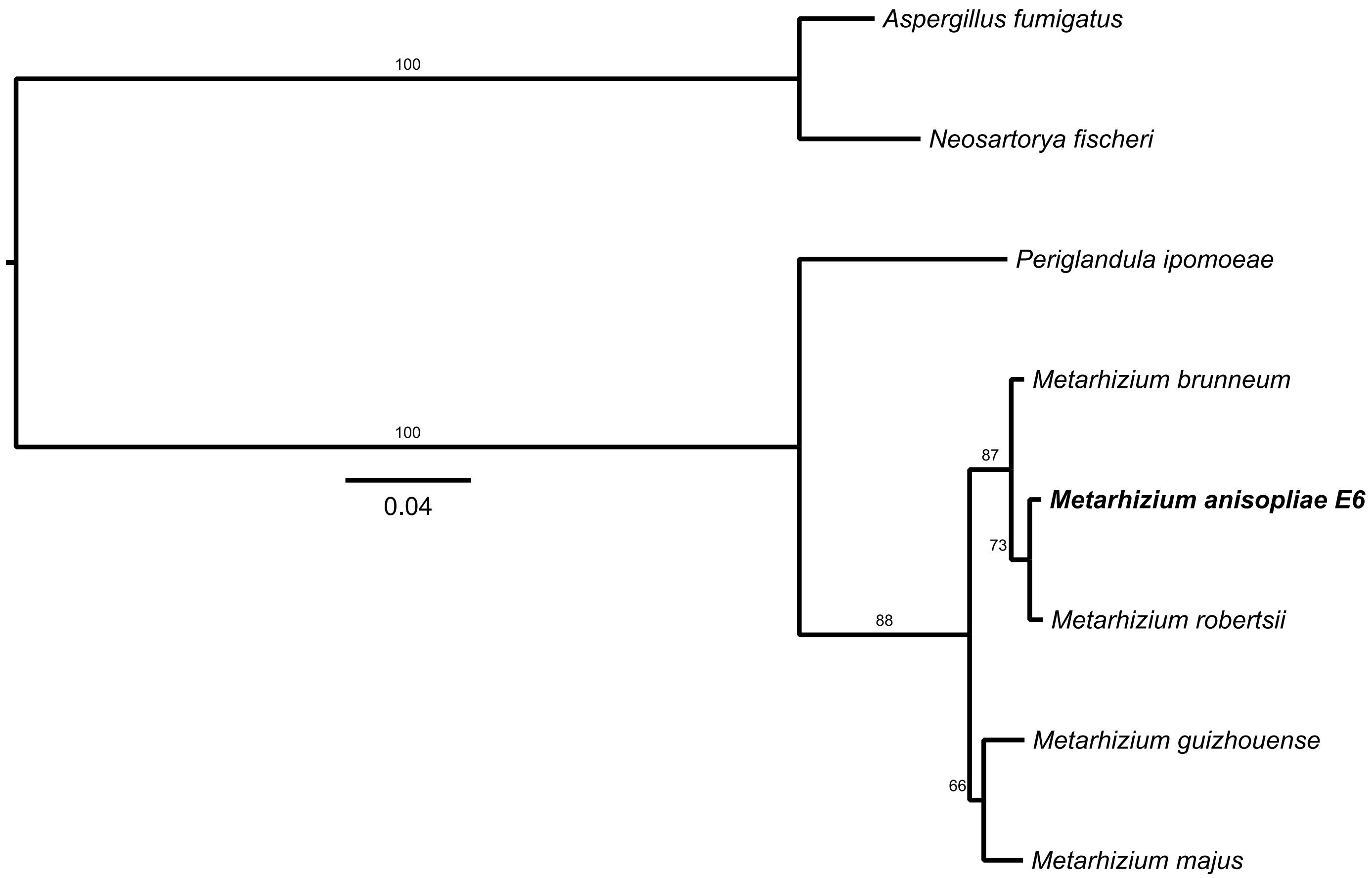

Figure 1: Maximum-likelihood tree for MANI_010512. The gene MANI_010512 assists in helvolic acid biosynthesis. Branches support values (bootstrap proportions) are associated with nodes.

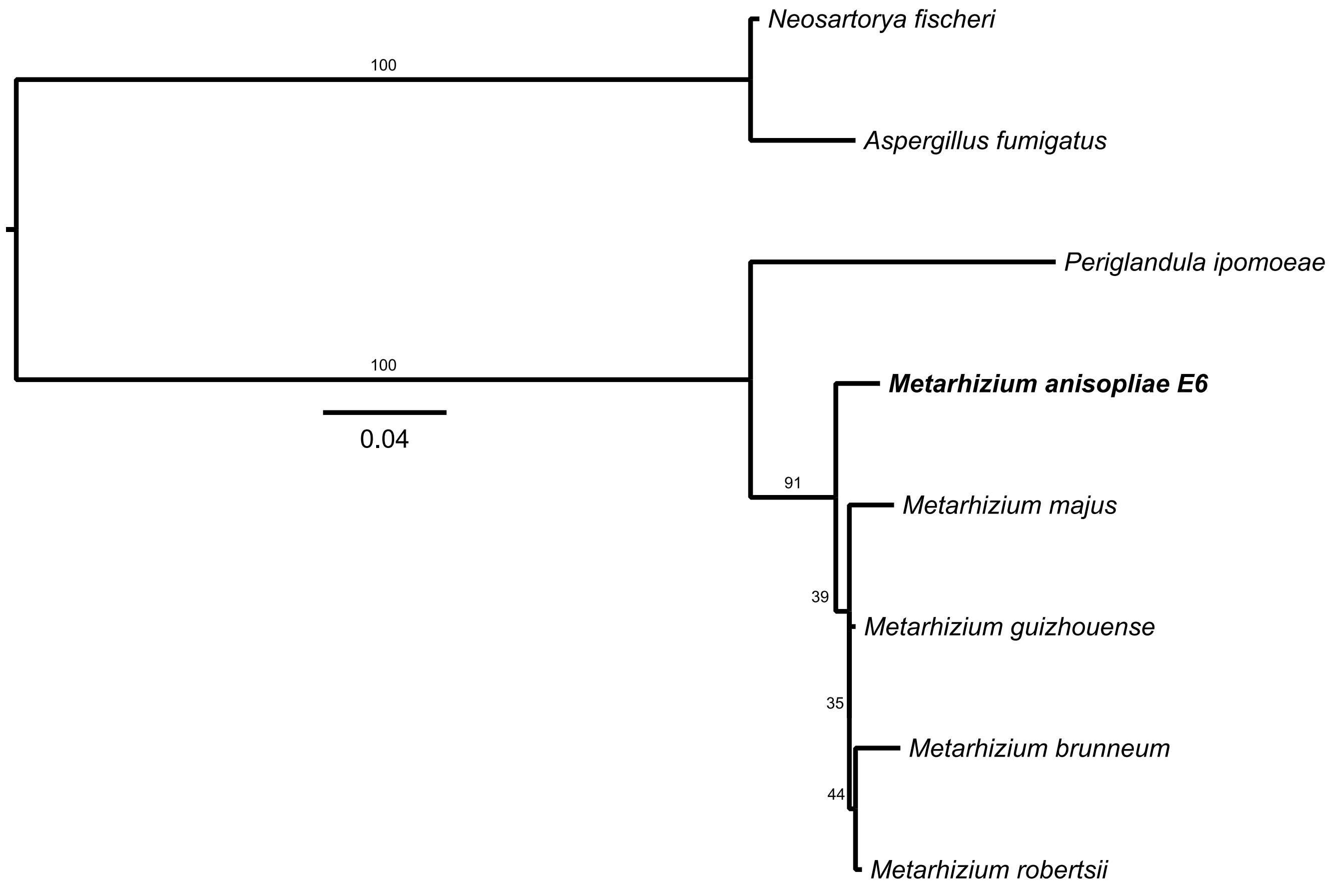

Figure 2: Maximum-likelihood tree for MANI_010527. The gene MANI_010527 assists in helvolic acid biosynthesis. Branches support values (bootstrap proportions) are associated with nodes.

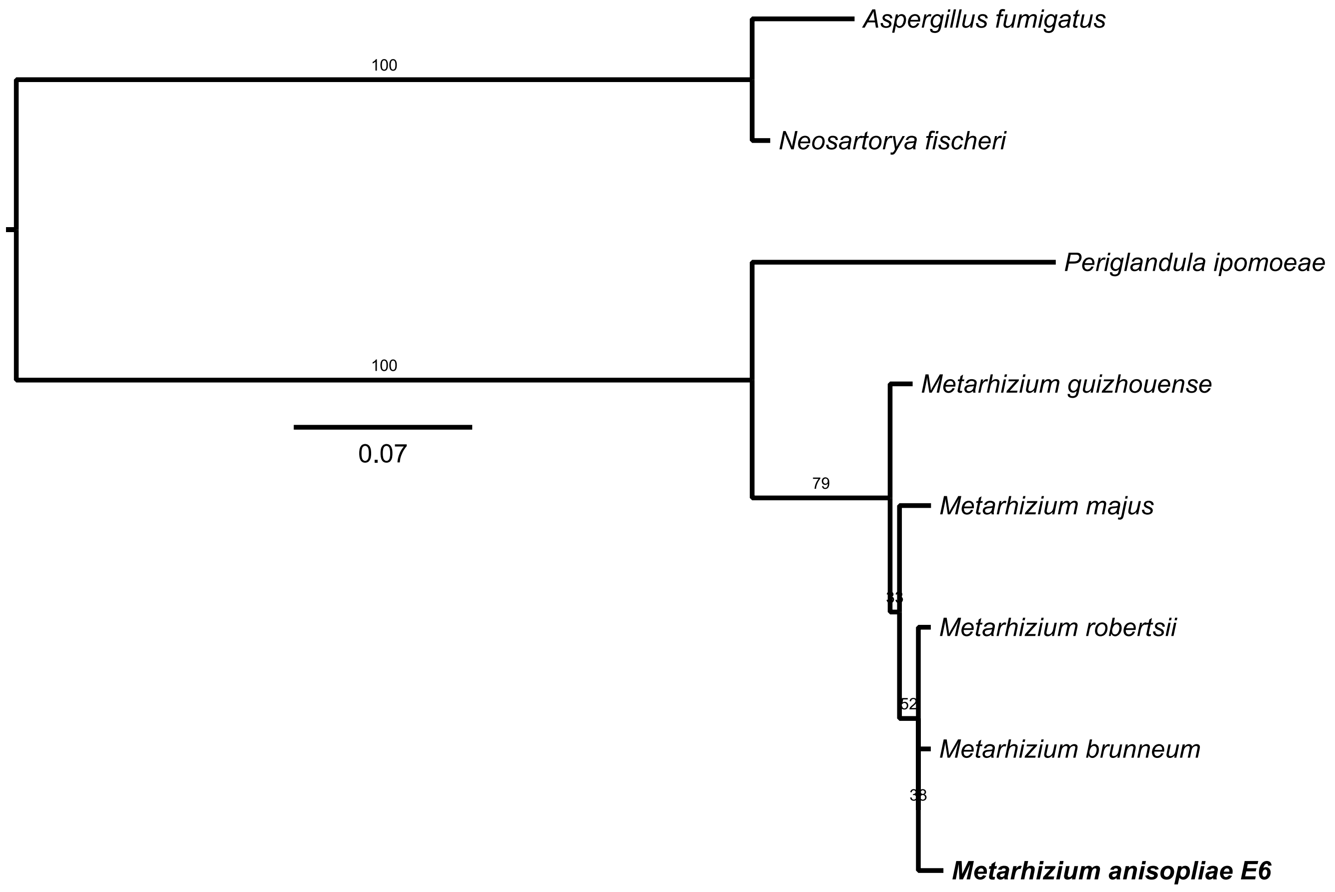

Figure 3: Maximum-likelihood tree for MANI_010530. The gene MANI_010530 assists in helvolic acid biosynthesis. Branches support values (bootstrap proportions) are associated with nodes.

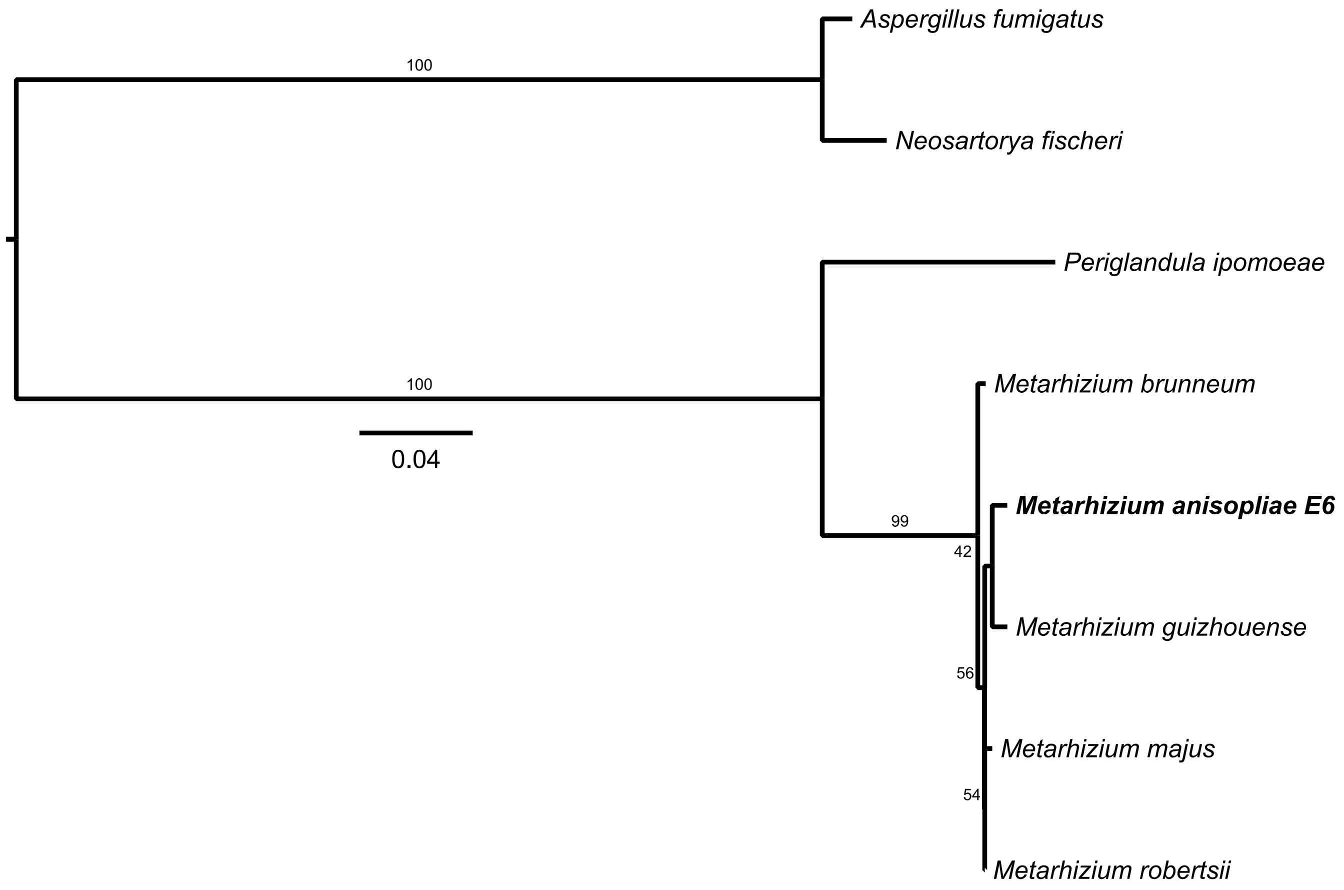

Figure 4: Maximum-likelihood tree for MANI_010531. The gene MANI_010531 assists in helvolic acid biosynthesis. Branches support values (bootstrap proportions) are associated with nodes.

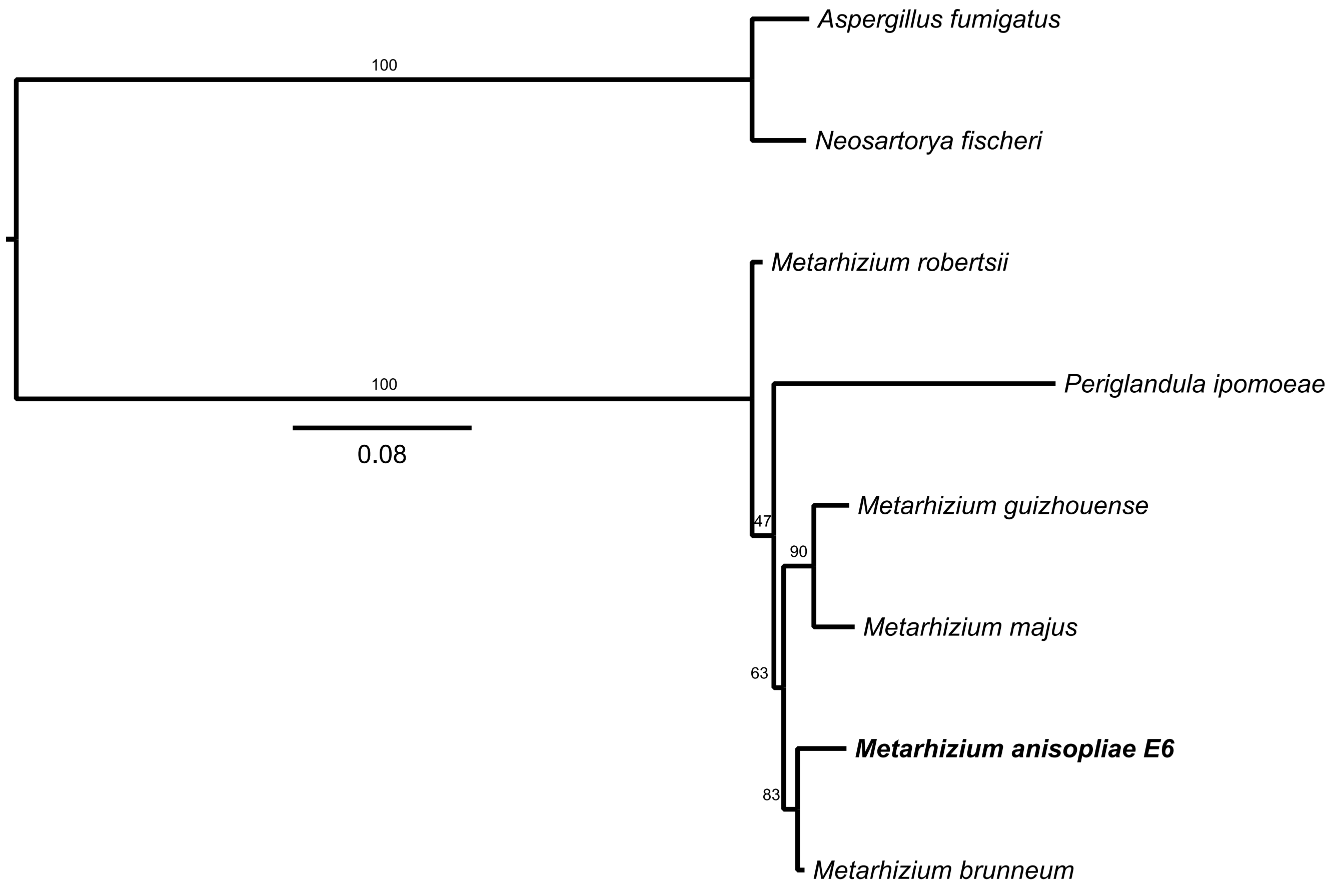

Figure 5: Maximum-likelihood tree for MANI_010532. The gene MANI_010532 assists in helvolic acid biosynthesis. Branches support values (bootstrap proportions) are associated with nodes.

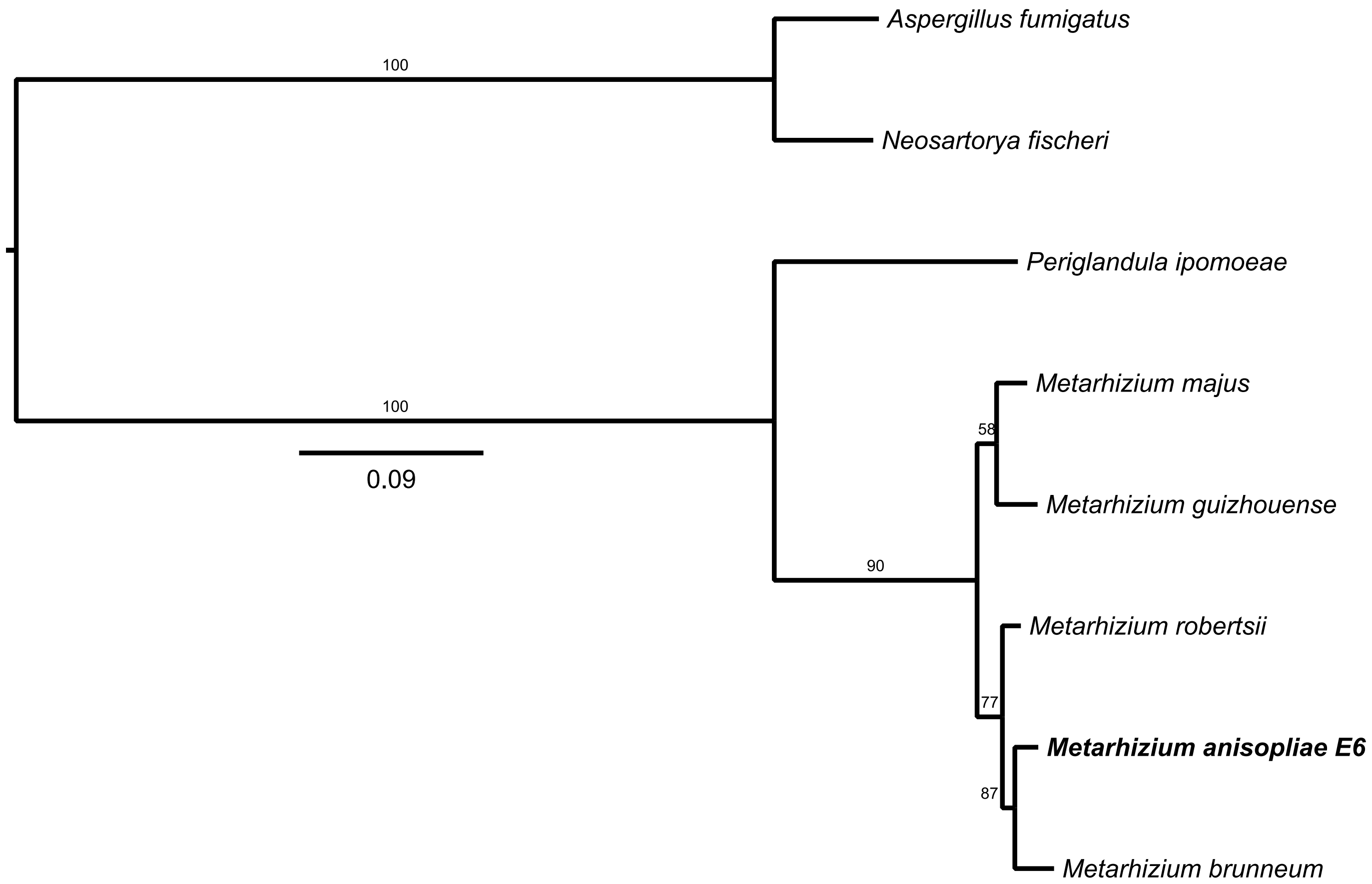

Figure 6: Maximum-likelihood tree for MANI_010536. The gene MANI_010536 assists in helvolic acid biosynthesis. Branches support values (bootstrap proportions) are associated with nodes.

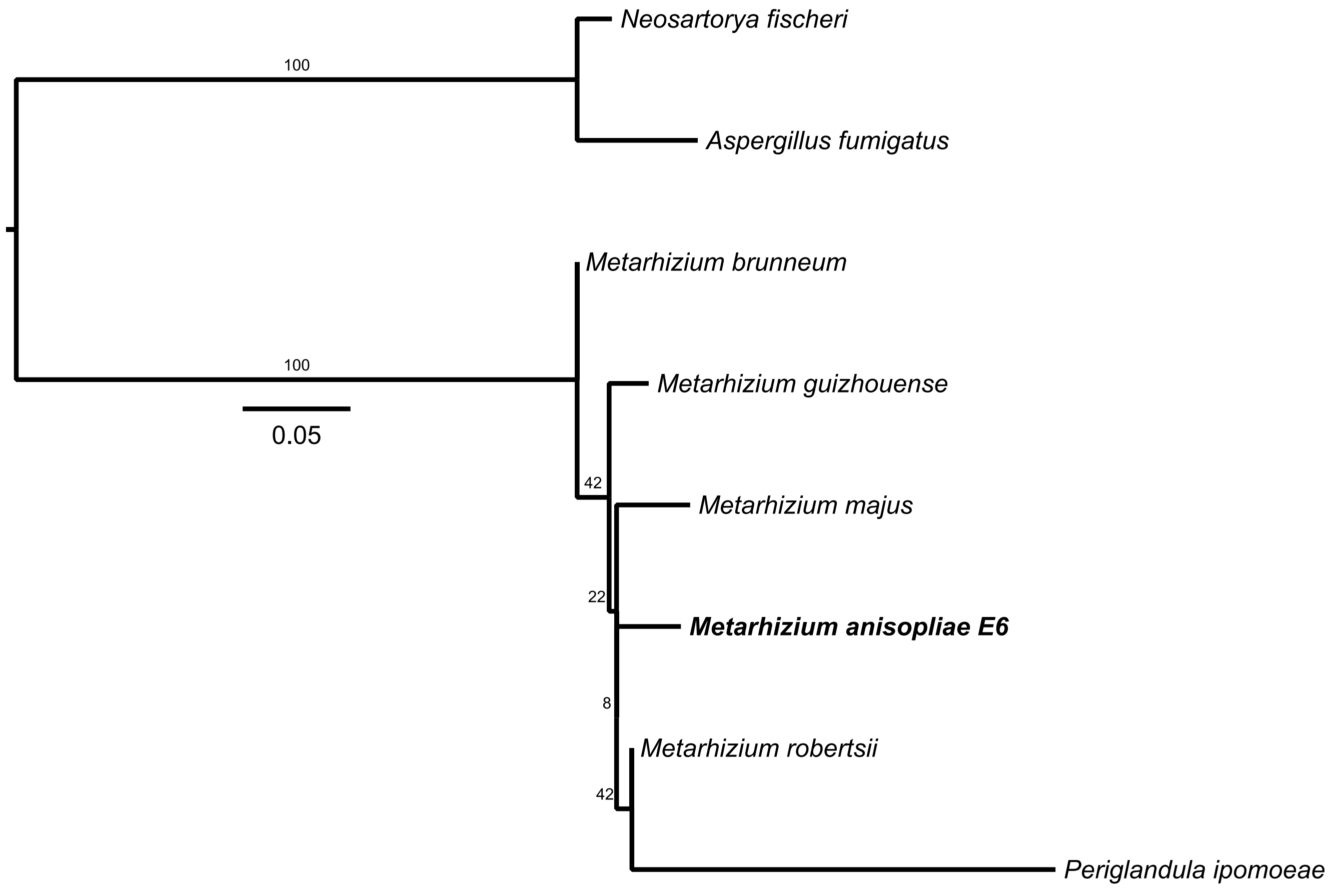

Figure 7: Maximum-likelihood tree for MANI_010537. The gene MANI_010537 assists in helvolic acid biosynthesis. Branches support values (bootstrap proportions) are associated with nodes.

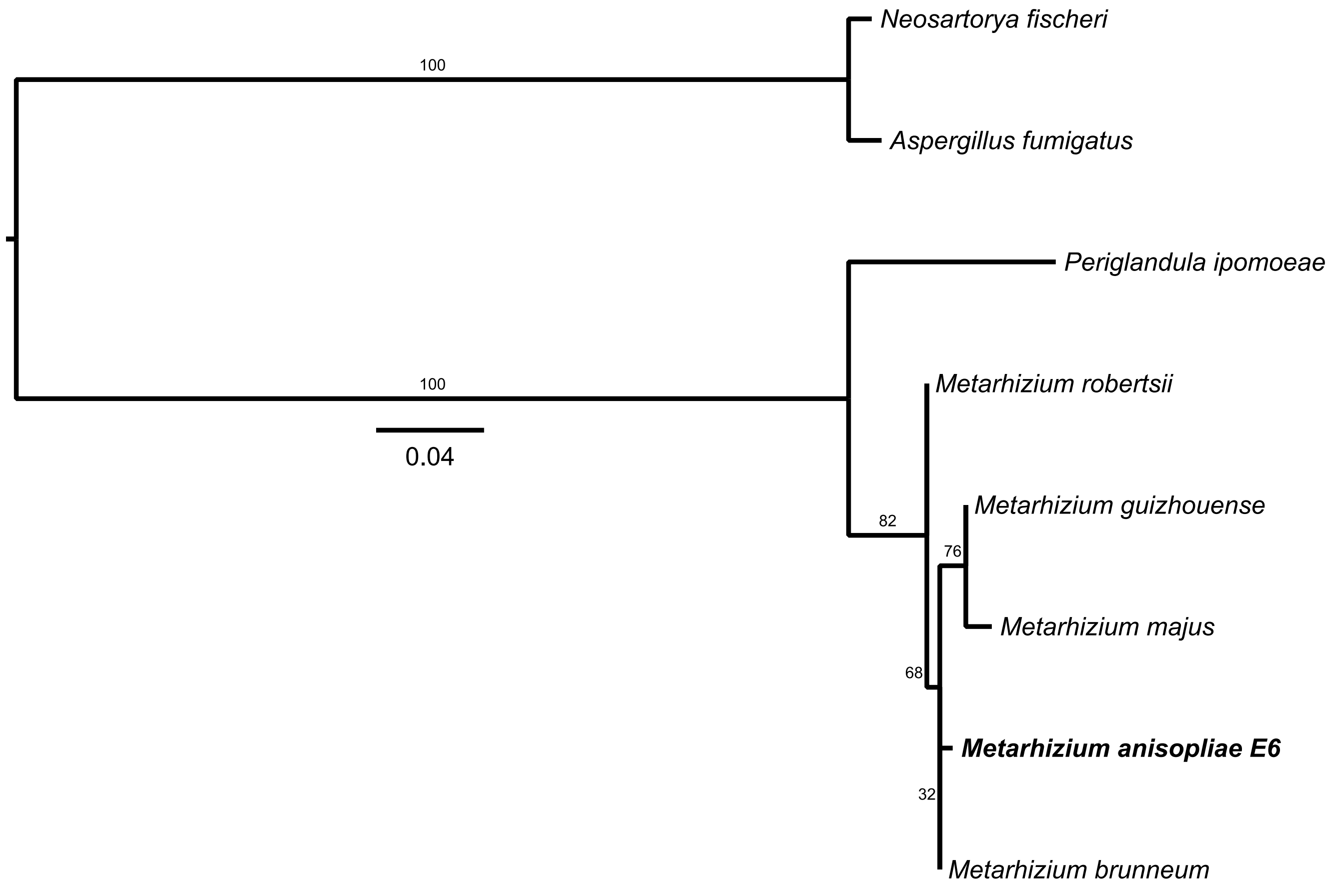

Figure 8: Maximum-likelihood tree for MANI_010594. The gene MANI_010594 assists in helvolic acid biosynthesis. Branches support values (bootstrap proportions) are associated with nodes.

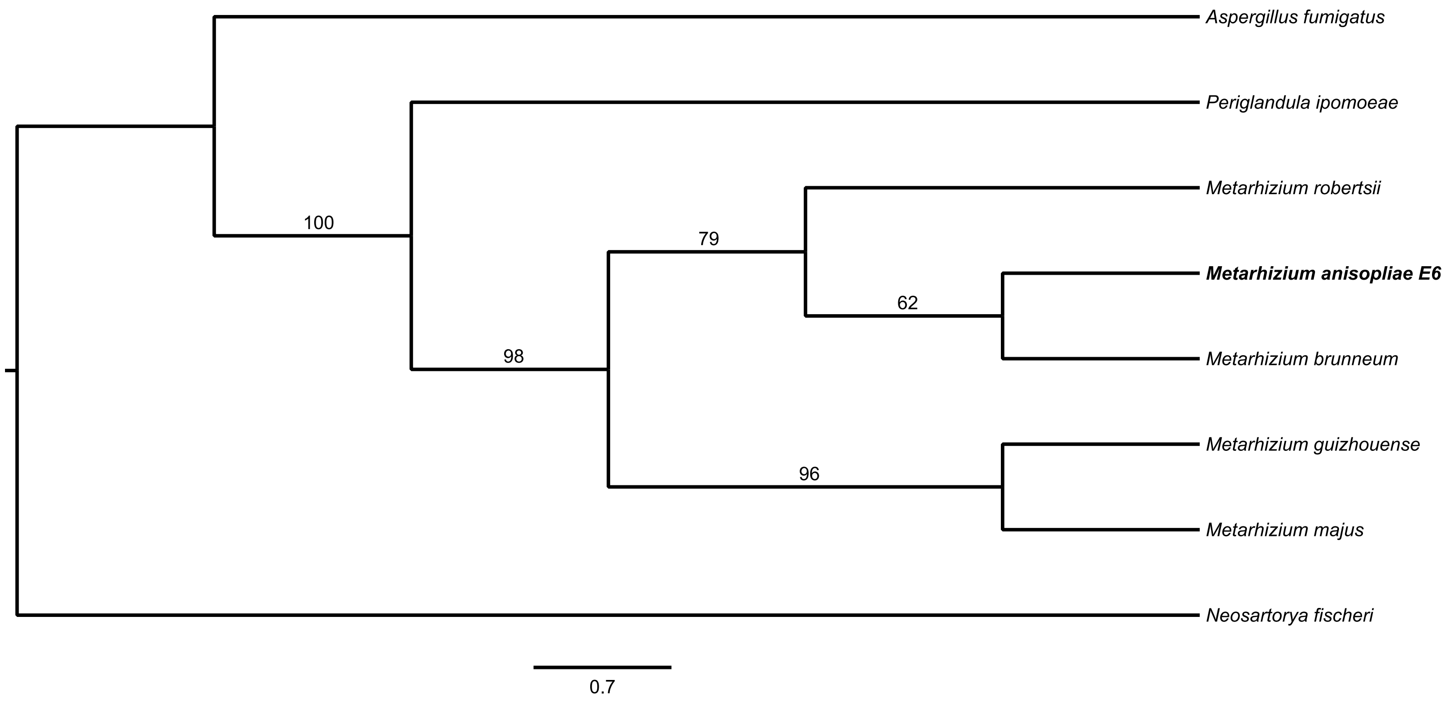

Figure 9: Supertree. MRP supertree constructed based on the inferred trees for helvolic acid cluster with CLANN 3.1.3. Branches support values (bootstrap proportions) are associated with nodes.

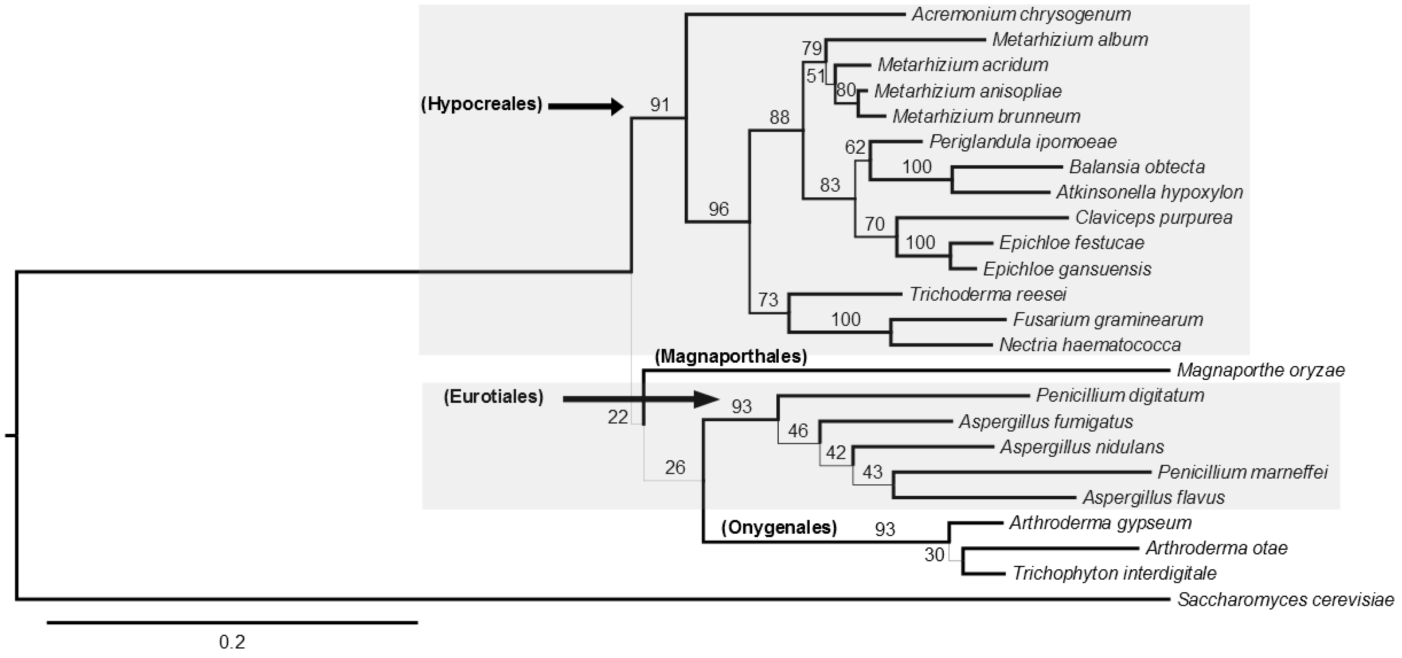

Figure 10: Species tree. Maximum-likelihood phylogeny for *tef1*, a barcode gene, showing the established species relationships. Branches support values (bootstrap proportions) are associated with nodes.
